# Long-range interactions between proximal and distal regulatory regions in maize

**DOI:** 10.1038/s41467-019-10603-4

**Published:** 2019-06-14

**Authors:** En Li, Han Liu, Liangliang Huang, Xiangbo Zhang, Xiaomei Dong, Weibin Song, Haiming Zhao, Jinsheng Lai

**Affiliations:** 1 State Key Laboratory of Agrobiotechnology and National Maize Improvement Center, Department of Plant Genetics and Breeding, China Agricultural University, 100193 Beijing, China; 2 Center for Crop Functional Genomics and Molecular Breeding, China Agricultural University, 100193 Beijing, China

**Keywords:** Epigenomics, Transcriptional regulatory elements, Agricultural genetics, Epigenomics

## Abstract

Long-range chromatin interactions are important for transcriptional regulation of genes, many of which are related to complex agronomics traits. However, the pattern of three-dimensional chromatin interactions remains unclear in plants. Here we report the generation of chromatin interaction analysis by paired-end tag sequencing (ChIA-PET) data and the construction of extensive H3K4me3- and H3K27ac-centered chromatin interaction maps in maize. Results show that the interacting patterns between proximal and distal regulatory regions of genes are highly complex and dynamic. Genes with chromatin interactions have higher expression levels than those without interactions. Genes with proximal-proximal interactions prefer to be transcriptionally coordinated. Tissue-specific proximal–distal interactions are associated with tissue-specific expression of genes. Interactions between proximal and distal regulatory regions further interweave into organized network communities that are enriched in specific biological functions. The high-resolution chromatin interaction maps will help to understand the transcription regulation of genes associated with complex agronomic traits of maize.

## Abstract (web summary)

Chromatin interaction analysis by paired-end tag sequencing (ChIA-PET) can discover specific protein-centered chromatin interactions in high resolution. Here, the authors use ChIA-PET to reveal the complex and dynamic interactions between proximal and distal regulatory regions of genes in maize.

## Introduction

Enhancers, important transcriptional regulatory elements, play important roles in modulating the expressional tissue specificity as well as the level of gene expression. A number of chromatin modifications including H3K4me1, H3K27ac, and histone acetyltransferase p300 have been shown to be closely associated with enhancers in mammals^1,2^. Using chromatin immunoprecipitation and sequencing (ChIP-seq), genome-wide identifications of enhancers were carried out in human and mouse, with thousands of putative enhancers identified. Similar enhancer identification efforts have also recently been done in *Arabidopsis*, rice, and maize^3^. Since enhancers function independently of their location relative to their targeted genes, it is difficult to link enhancers to their corresponding target genes by ChIP-seq alone. Studies on higher-order chromatin architecture indicated that enhancers and promoters can often form DNA loops to exert function^4^, which gives an opportunity to connect the distantly located enhancers and promoters through proximity ligation.

Theoretically, Hi-C or other Hi-C-like chromosome conformation capture (3C)-derived techniques can capture all chromatin interactions, which also include the interaction of promoters and enhancers. However, typical Hi-C experiments need extremely deep-sequencing depth to reach a moderately high resolution (20–50 kb)^5^ and are often accompanied by vast background noises. The limitation of using Hi-C to capture promoter–enhancer interactions become even bigger for species with larger genome size, as the amount of data required for Hi-C to reach the same level of resolution increases exponentially when the size of genome grows. Chromatin interaction analysis by paired-end tag sequencing (ChIA-PET), an approach through enriching particular subset of interactions associated with chromatin modification or protein of interest, has the power of discovering specific protein-centered chromatin interactions at high resolution (<5 kb)^6^ with practical amount of sequencing data. Taking advantage of ChIA-PET, several studies that focus on the interactions between genes and enhancers have been reported in mammals^6–8^. However, there are no reports of ChIA-PET studies in plants so far, albeit several Hi-C experiments had been done in plants very recently and showed that, similar to animals, plants also have highly complex higher-order chromatin architecture^9–11^.

Maize (*Zea mays*), a major crop worldwide, is an excellent model system for plant genetic and genomic research. In previous reports, several examples of long-distance regulatory elements have been genetically identified in maize, such as *TB1*, *UB3*, *ZmRap2*.*7* and *BX1*^12–15^, which are all related to important agronomic traits. In addition, 3C study in maize had revealed that chromatin loops around the paramutagenic *b1* locus were associated with the expression of B1 protein^16^. In this study, we apply ChIA-PET, with antibodies against histone modifications of H3K4me3 and H3K27ac, chromatin modifications enriched at putative promoters and enhancers, respectively^17,18^, to construct H3K4me3-centered and H3K27ac-centered chromatin interaction maps in immature ear and shoot of maize. There are 24,105 and 24,218 long-range intra-chromosomal interactions identified in immature ear and shoot, respectively. Chromatin interactions are organized into functional network communities, which show considerable conservation between tissues. Our analysis provides a three-dimensional perspective to explore the regulatory mechanism of genes related to complex agronomic traits in maize.

## Results

### High-resolution maps of chromatin interaction in maize

To map the three-dimensional chromatin interactions associated with gene expression in maize, we adopted the existing ChIA-PET protocol^7^. Two antibodies of H3K4me3 and H3K27ac, which are histone modifications that are enriched at active promoter^17^ and enhancer^3,18,19^, respectively, were used to capture the associated chromatin interactions in immature ear and shoot. More than one billion read pairs were produced for each sample with two biological replicates. About 100–150 million uniquely mapped read pairs were generated for each ChIA-PET library (Supplementary Table 1). In order to remove self-ligation reads and identify long-distance loops, we filtered PETs (read pair with chromatin interaction) with distance shorter than 10 kb. There was high reproducibility between replicates as indicated by the repeatability of biological replicates at different levels, including library reads and peaks of chromatin interaction PETs (Supplementary Fig. 1). To confirm and explore the binding sites of H3K4me3 and H3K27ac, we also conducted ChIP-seq experiments with the same antibodies used for ChIA-PET (Supplementary Table 2).

**Fig. 1:**
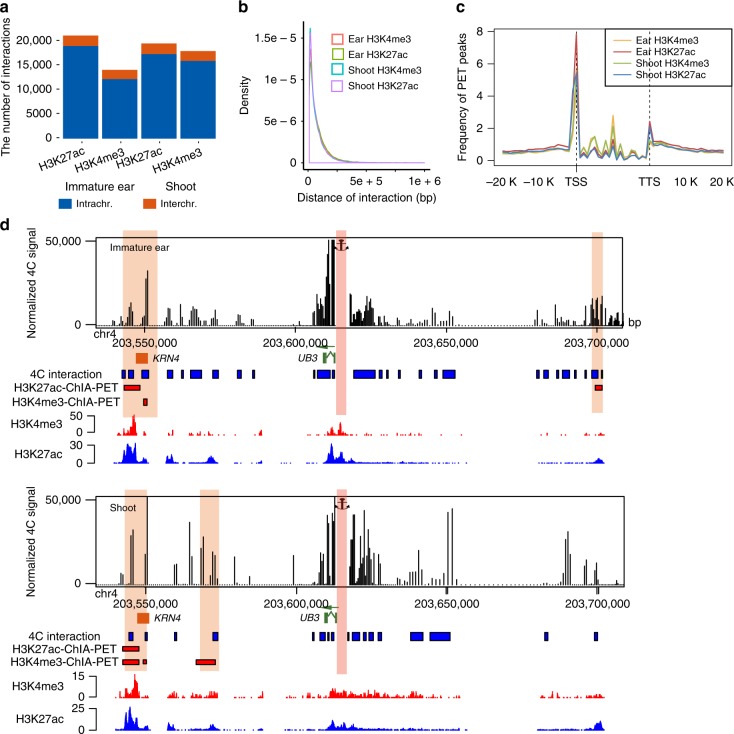
Identification of chromatin interactions in immature ear and shoot. **a** The number of intra-chromosomal interactions (intrachr.) and inter-chromosomal interactions (interchr.) identified in H3K27ac-mediated and H3K4me3-mediate chromatin interaction analysis by paired-end tag sequencing (ChIA-PET) in immature ear and shoot. **b** The density distribution of chromatin interactions along the distance in H3K27ac-mediated and H3K4me3-mediate ChIA-PET in immature ear and shoot. **c** The profile of PET peaks around gene body of H3K27ac-mediated and H3K4me3-mediate ChIA-PET in immature ear and shoot. **d** 4C validation for the promoter of *UB3* as viewpoint in immature ear and shoot. The blue rectangles indicate the regions interacting with viewpoints in 4C experiments. The red rectangles indicate the regions interacting with viewpoints in ChIA-PET datasets. The orange rectangle represents the previously reported regulatory region (*KRN4*) of *UB3*. Source Data of Fig. 1d are provided as a Source Data file

Next, two biological replicates were combined together for an inclusive view of chromatin interactome. We filtered out the technical noise based on statistical analyses and identified high-confidence binding sites and interacting PET peaks (see Methods and Supplementary Fig. 2). A least three PETs were required for high-confidence interaction calling. The full pipeline for interaction identification is available in Supplementary Fig. 2. We identified 13,999 H3K4me3-related and 20,979 H3K27ac-related high-confidence (false discovery rate (FDR) <0.01) interactions in immature ear. Similarly, 17,821 H3K4me3-related and 19,383 H3K27ac-related chromatin interaction were identified in shoot. About 90% high-confidence interactions were intra-chromosomal interactions in all experiments, similar to results of previous ChIA-PET studies in human^6^ (Fig. 1a, Supplementary Table 3, and Supplementary Data 1–4). The majority of intra-chromosomal interactions were within 100 kb, with a median distance of near 45 kb (Fig. 1b). The median length of peaks of intra-chromosomal PETs was about 5 kb in all datasets, displaying the high-resolution interaction data we generated. Both H3K4me3-related and H3K27ac-related interaction PET peaks showed strong enrichments at transcriptional start site (TSS) of genes and relatively weaker enrichments at transcriptional terminal sites (TTS) in both tissues (Fig. 1c), which were consistent with the patterns of H3K4me3 and H3K27ac peaks from ChIP-seq datasets (Supplementary Fig. 3a, Supplementary Data 5–8). About 60% H3K4me3 peaks and nearly 50% H3K27ac peaks were involved in chromatin interactions, which showed higher occupancy than those without interactions in all samples (Supplementary Fig. 3b, c). This result indicated that most highly enriched H3K4me3/H3K27ac binding sites were involved in chromatin interactions.

To further validate our ChIA-PET results, we conducted 4C-seq experiments for three selected genes, including *TB1*, *UB3*, and *Rad51a*, and one randomly selected enhancer region (D5978), in both immature ear and shoot. All information of the primer sequences and viewpoints used in 4C were listed in Supplementary Table 4. As a result, 95% of ChIA-PET interactions were also identified by 4C-seq, indicating the high rate of validation for our ChIA-PET data. For example, we chose *UB3* as the viewpoint. ChIA-PET datasets showed that there were three regions (including *KRN4* site^13^) interacting with *UB3* in both immature ear and shoot. 4C results showed that all these three regions showed high normalized 4C signal (Fig. 1d). For *TB1* viewpoint, we identified seven in immature ear and four regions in shoot, including the reported regulatory region^12^, interacting with *TB1* in ChIA-PET datasets, respectively (Supplementary Fig. 4). There was only one region without high normalized 4C signal in both immature ear and shoot. All the interactions identified in ChIA-PET data associated with *Rad51a* were also identified in 4C experiments in both tissues (Supplementary Fig. 5). Only one out of seven regions interacting with the selected enhancer D5978 region identified in ChIA-PET was without high normalized 4C signal (Supplementary Fig. 6). Overall, our datasets represented a high-resolution genome-wide map of H3K4me3/H3K27ac-mediated long-range interactions of immature ear and shoot in maize.

Considering the functional redundancy of the two histone modification-mediated chromatin interactions, we compared interactions related to these two modifications in both tissues. About 70% intra-chromosomal PET peaks were common between two modification-mediated ChIA-PET datasets (Supplementary Fig. 7a), and nearly 50% intra-chromosomal interactions were identified in the two histone modification-mediated datasets in both immature ear and shoot (Supplementary Fig. 7b). The common intra-chromosomal interactions had lower FDR values and more mapped PETs than unique intra-chromosomal interactions between two histone modification-mediated chromatin interactions (Supplementary Fig. 7c), indicating that these chromatin interactions were of higher confidence and our technologies were of high reliability. Further, we combined the two histone modification-mediated ChIA-PET datasets, and identified 24,105 and 24,218 long-range intra-chromosomal interactions in immature ear and shoot, respectively.

### Identification of distal regulatory regions in maize

**Fig. 2:**
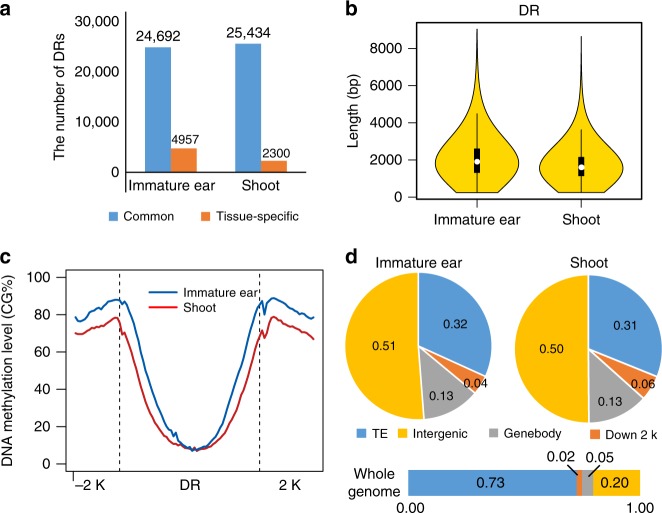
Identification of distal regulatory regions (DRs) in immature ear and shoot. **a** The bar plots show the number of common and tissue-specific DRs in immature ear and shoot. **b** Violin plots for length of DRs in immature ear and shoot. **c** The profile of DNA methylation (CG context) around DR in immature ear and shoot. The DNA methylation level^22,23^ (CG context) of DRs was significantly lower than that of their flanking regions. **d** The distribution of DRs in different genomic regions in two tissues

As H3K27ac is reported to be enriched at active enhancer in animals^18^, we identified genome-wide distal regulatory regions (DRs) by using ChIP-seq data of H3K27ac. H3K27ac peaks that were at least 2 kb away from TSS of gene were annotated as DRs. As a result, 29,649 and 27,734 DRs were identified in immature ear and shoot (Supplementary Data 9–10), respectively. There were 24,692 and 25,434 common DRs identified in immature ear and shoot, respectively, with 4957 only identified in immature ear and 2300 only in shoot (Fig. 2a). The average lengths of DRs were 2215 bp and 1900 bp in immature ear and shoot, respectively (Fig. 2b). Extensive studies in animals suggested that DRs typically had low DNA methylation level and high DNA accessibility^20,21^. The DNA methylation level^22,23^ (CG context) of DRs we identified was around 0.2, which was significantly lower than that of their flanking regions (Fig. 2c). Both ATAC-seq^10^ and DNase-seq results^24^ showed much higher read level around DRs than their flanking regions (Supplementary Fig. 8a, b), implying the high DNA accessibility of these DRs.

To investigate the distribution of DRs in maize genome, we divided the genome into different regions, including transposable elements (TE), downstream 2 kb of gene body, gene body, and the remaining intergenic regions (Fig. 2d). The results showed that about 50% DRs located in intergenic regions and 30% in TE regions, in agreement with previous reports in maize by using H3K9ac ChIP-seq and DNase-seq methods^19,24^. About 56% and 53% of these DR-like TE sequences were Helitron in immature ear and shoot, respectively; and 42% and 45% were long terminal repeat retrotransposon (Supplementary Data 11–12). About 1600 (18%) of all the DR-like TE sequences had chromatin interactions in our ChIA-PET data in both tissues. We found that TE sequences overlapping with DRs also displayed lower DNA methylation level than their flanking regions or TEs not overlapping with DR (Supplementary Fig. 8c, d), suggesting that these TE sequences could probably have DR-related function.

### Annotation of chromatin interactions in maize

**Fig. 3:**
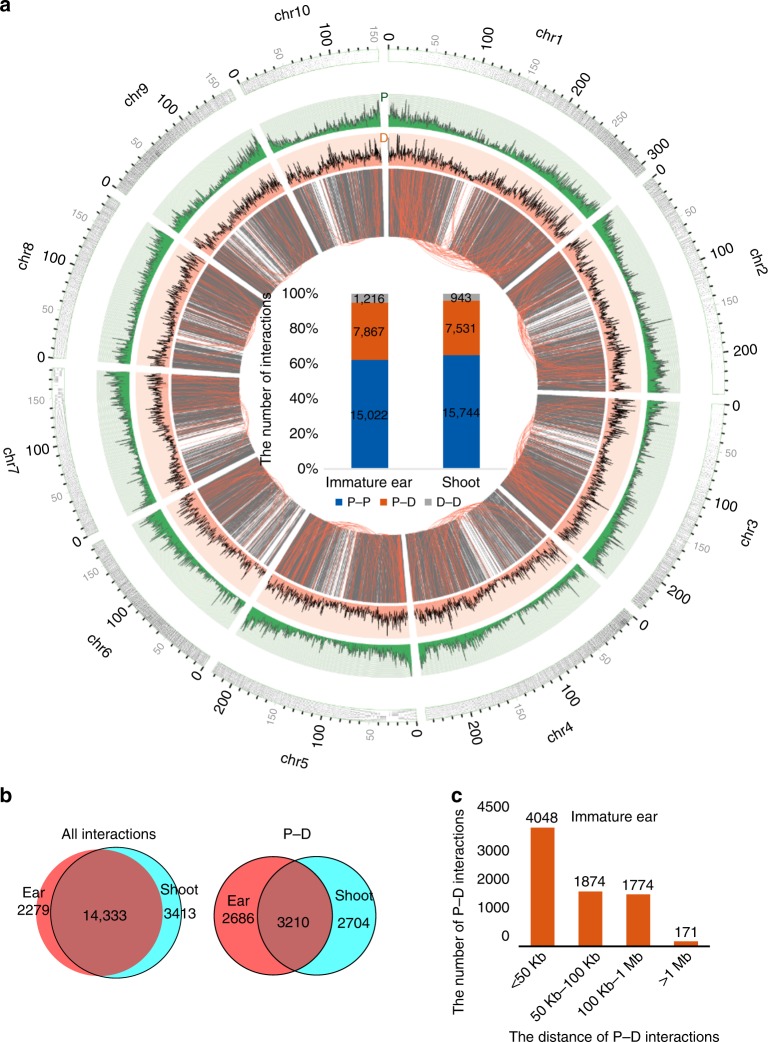
Annotation of chromatin interactions. **a** Circos map of the whole-genome chromatin interactions from chromosome 1 to chromosome 10 in immature ear, generated using the Circos software package (http://circos.ca/). Intra-chromosomal interactions are drawn in the innermost ring, followed by the distal regulatory region (DR) (dark orange) and gene density track (dark green). Chromatin interactions with distance longer than 1 Mb are highlighted by orange arcs. The histogram in center region of Circos map showed the number of proximal–proximal (P–P), proximal–distal (P–D), distal–distal (D–D) chromatin interactions in immature ear and shoot, respectively. **b** Venn diagram of the number of P associated with all intra-chromosomal interactions and P–D interactions in immature ear and shoot. **c** Summary of the number of P–D interaction with variable distances in immature ear

Considering the antibodies used in our experiments, we annotated the PET peaks as proximal region or DR. PET peaks within 2 kb around TSS of genes were annotated as proximal PET peaks (P). PET peaks that were over 2 kb away from TSS of genes were annotated as distal PET peaks (D). There were 16,612 P and 6421 D involved in long-range chromatin interactions in immature ear, and 17,094 P interacting with 6460 D in shoot. All intra-chromosomal interactions were classified into three groups: proximal–proximal (P–P), proximal–distal (P–D), and distal–distal (D–D) (Supplementary Fig. 9a). More than 60% of intra-chromosomal interactions were P–P interactions, followed by P–D (near 35%) and D–D interactions (5%) (Fig. 3a and Supplementary Fig. 9b). This hinted that the communications among genes were predominantly frequent, which was also consistent with results reported in human^6,8,25^.

The median lengths of P and D involved in intra-chromosomal interactions were about 6.5 and 3.5 kb in our dataset (Supplementary Fig. 9c). There were 14,333 P identified with interactions in both immature ear and shoot. While 2279 P with interactions were identified only in immature ear and 3413 P with interactions were identified only in shoot (Fig. 3b). Moreover, there were 5896 and 5914 P interacting with D in immature ear and shoot, respectively. There were 3210 P identified to interact with D in both tissues, and 2686 and 2704 P were identified to interact with D only in immature ear and shoot (Fig. 3b), respectively. The average numbers of promoters in P interacting with D were 1.41 and 1.47 in immature ear and shoot (Supplementary Fig. 9d), which were slightly more than P with interaction, implying that genes regulated by D prefer to cluster together in the genome.

About 50% interactions were within 50 kb in both tissues. There were 595 and 603 chromatin interactions with distance longer than 1 Mb in immature ear and shoot, respectively (Supplementary Fig. 9e). Previous studies in human have showed that enhancers prefer to regulate nearby genes^1,26^. Most (nearly 80%) of the P–D interactions were within 100 kb and no more than 3% were >1 Mb in both tissues (Fig. 3c, Supplementary Fig. 9f). Although many enhancers tend to interact with linearly close-by genes, it is worthy to note that there were still 40% D, a number very similar to the results in human^6^, discarding their adjacent genes and interacting with distant genes. About 65% of these long-range P–D interactions skipped typically expressed genes, and 85% of these skipped genes that were syntenic when compared with sorghum, in both tissues.

### Genome-wide chromatin interaction pattern in maize

**Fig. 4:**
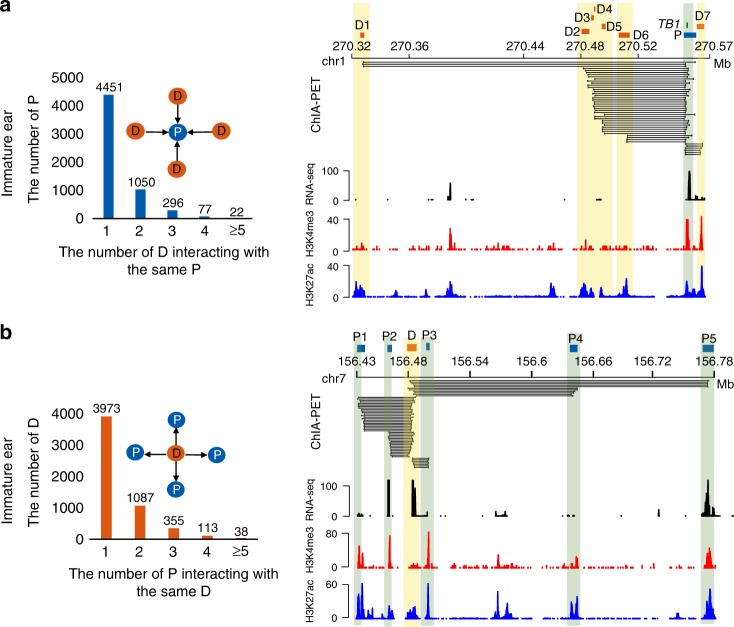
Chromatin interaction mode in immature ear. **a** The left panel is the summary bar chart showed the number of proximal paired-end tag (PET) peaks (P) (*x* axis) interacting with various numbers of distal PET peaks (D) (*y* axis) in immature ear. The corresponding right panel is an example of one promoter (*TB1*) interacting with seven D in immature ear. **b** The left panel is the summary bar chart showed the number of D (*x* axis) interacting with various number of P (*y* axis) in immature ear. The corresponding right panel is an example of one D interacted with five P in immature ear. The green rectangle represents the corresponding gene. The blue and orange rectangles indicate P and D, respectively. P was highlighted in dark green, and D was highlighted in yellow. Source Data of Fig. 4a, b are provided as a Source Data file

Studies in human and mouse indicated that the regulatory relationship between enhancer and promoter are not always one-to-one^8,27^. Our data in maize showed that 75% in immature ear and 78% in shoot P interacted with only one D, while about 25% P interacted with multiple D in both tissues (Fig. 4a and Supplementary Fig. 10a), consistent with the results in human^8^. The average number of D interacting with one P was 1.33 and 1.27 in immature ear and shoot, respectively, which was marginally smaller than that in human^28^. *TB1* gene, a large effect quantitative trait locus existing in modern domesticated maize whose high expression increases apical dominance in maize compared to its wild ancestor, teosinte^29^, has been studied extensively about its regulatory mechanism. We found seven D, including the reported regulatory region ~58 kb upstream of gene, interacting with *TB1* in immature ear^12,30^ (Fig. 4a). *Rough sheath1* (*RS1*) gene, a member of *KNOX* gene family affecting plant architecture^31^, with a high transcription level in meristems including shoot tips, regulates cell fate of leaf in maize^32^. Four D were found interacting with *RS1* in shoot (Supplementary Fig. 10a). *Brassinosteroid-deficient dwarf1* (*BRD1*) gene encodes a brassinosteroid C-6 oxidase that catalyzes the final steps of brassinosteroid synthesis^33^, whose corresponding mutant had a dwarf plant phenotype. We identified five D interacting with *BRD1* in shoot, which may coordinately regulate the expression of *BRD1* (Supplementary Fig. 10c). The possibility of P interacting with multiple D may provide a structure basis of functional redundancy or multiple ways for gene activation.

Subsequently, we explored the number of D interacting with one P. About 71% of immature ear and 74% of shoot D interacted with only one P, while about 30% D interacted with two or more P in both tissues (Fig. 4b and Supplementary Fig. 10b). The average number of P interacting with one D was 2.01 both in immature ear and shoot. *SBP29* gene, a member of SQUAMOSA promoter binding protein-like family, has been found to be associated with flowering time in maize^34^. The promoter region of *SBP29* and four other P (Zm00001d021571/Zm00001d021572, Zm00001d021574, Zm00001d021576/Zm00001d021577, and Zm00001d021579) interacted with one D in immature ear (Fig. 4b) where *SBP29* had much higher expression level than in shoot. In shoot, we found one D interacting with *RAMOSA3* (*RA3*)^35^, as well as three other genes (Supplementary Fig. 10b), including Zm00001d022190, Zm00001d022191, and Zm00001d022194. Genes interacting with the same D were prone to be coordinately expressed (*p* <2.2e − 16, two-sided *t* test) (Supplementary Fig. 10d), implying that these genes may participate in related functional pathways.

### Correlation between interaction and gene expression

**Fig. 5:**
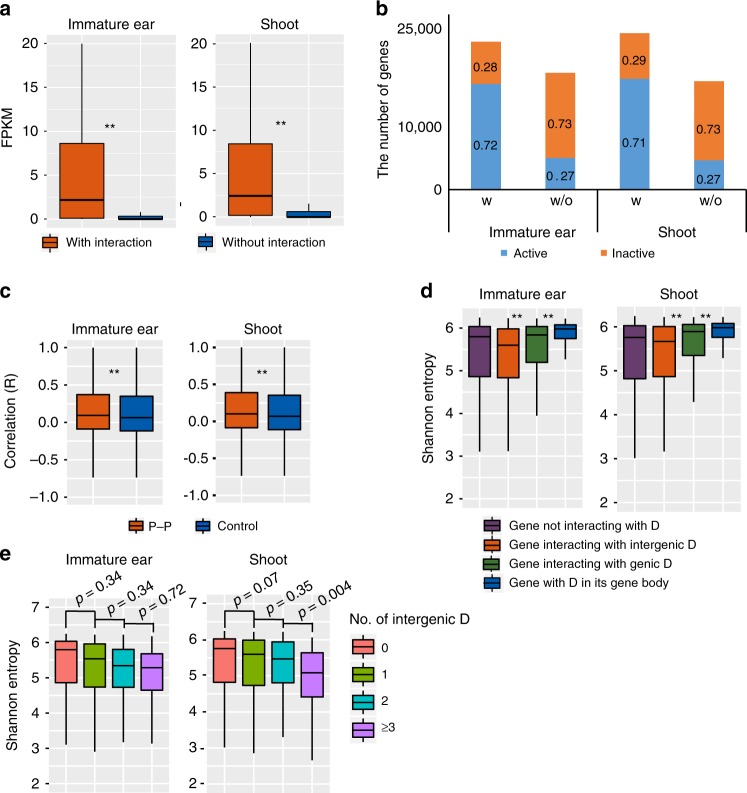
The association of chromatin interaction and gene expression. **a** The comparison of fragments per kilobase of transcript per million (FPKM) for genes interacting or not interacting with chromatin interactions (**p <2.2e − 16, two-sided *t* test). **b** Transcriptional status of genes with (w) or without (w/o) interactions. The bar plot shows the number of corresponding genes with the proportion labeled on the bar. ‘Inactive’ indicates gene with FPKM <1, ‘Active’ indicates gene with FPKM ≥1. ‘w’ represents genes with chromatin interactions; ‘w/o’ represents genes without chromatin interactions. **c** The comparison of expression correlation between gene pairs with proximal–proximal (P–P) interaction and controls in immature ear and shoot (immature ear: **p = 2.521e − 08, shoot: **p = 3.6e − 14, two-sided *t* test). **d** The comparison of Shannon entropy of gene interacting with genic D or intergenic D and gene with genic D in its gene body. (**p <2.2e − 16, two-sided *t* test). **e** The comparison of Shannon entropy of gene interacting with different number of intergenic D. Gene not interacting with intergenic D and enriched with H3K4me3/H3K27ac signal were as control (two-sided *t* test). In the box plot, the central rectangle spans the first quartile to the third quartile (the interquartile range). A segment inside the rectangle shows the median and whiskers above and below the box show the locations of the minimum and maximum. We used the collected RNA-sequencing (RNA-seq) dataset as reported^38^, consisting of 53 different seeds and 25 non-seed samples in maize, and then measured the co-expression level of gene pairs and calculated Shannon entropy of each gene. Summary of the number of gene pairs or genes in this figure can be seen in Supplementary Table 5. Source Data of Fig. 5d, e are provided as a Source Data file

To understand the correlation between chromatin interaction and transcriptional activities of genes in maize, we generated the transcriptome datasets using RNA-sequencing (RNA-seq) of immature ear and shoot. The RNA-seq data we generated showed high degree of correlation (*R* = 0.988) between two biological replicates in both tissues (Supplementary Fig. 11a). Then we compared the expression pattern of genes with or without chromatin interactions. Genes with chromatin interactions had higher expression level than those without interactions (*p* <2.2e − 16, two-sided *t* test) (Fig. 5a). Interestingly, although the chromatin interactions associated with distal and proximal regulatory regions were captured by using active histone modifications (H3K4me3 and H3K27ac in this study), we still found 28% and 29% genes with interactions were not expressed or expressed very lowly (fragments per kilobase of transcript per million (FPKM) <1) in immature ear and shoot, respectively (Fig. 5b). In human and *Drosophila*, researchers have found that chromatin interactions can be established before the activation of genes, which may need other cell-specific transcriptional factors^5,36,37^. There were 27% genes with FPKM  ≥1 that had no chromatin interactions in both tissues (Fig. 5b), which may be due to the regulatory elements located within short distance (<10 kb) to their promoters, the datasets used are not saturated or the plant materials used may have some cell types underrepresented. Compared to those genes without P–P interactions, most genes with P–P interactions were active (Supplementary Fig. 11b). Previous studies in animals had reported that genes with P–P interactions were more likely to be transcriptionally coordinated^6^. To measure the co-expression levels of gene pairs with P–P interactions in maize, a RNA-seq dataset^38^ of 53 different seed and 25 non-seed samples were used to calculate the correlation levels of the expression levels of gene pairs with P–P interactions. The expression levels of gene pairs with P–P interactions had a significant higher correlation level than that of gene pairs in controls by randomly shifting P–P interactions 100 times and with similar H3K4me3/H3K27ac level as genes with P–P interactions (*p* <2.2e − 16, two-sided *t* test) (Fig. 5c, see Methods), which implied that genes involved in P–P interactions also tended to be transcriptionally coordinated and may function in related biological pathways. Besides, gene ontology (GO) term enrichment analysis showed that genes interacting with D were related to many regulation processes, including GO term regulation of biosynthetic process, regulation of RNA metabolic process, regulation of transcription, and so on (Supplementary Fig. 11c).

Previous studies in human have showed that enhancers were strongly correlated to the cell-type-specific gene expression^26^. Whether genes associated with enhancers are prone to be tissue-specific expressed has not been examined before in maize. The tissue specificity of gene expression is usually represented by Shannon entropy^39^. The higher the tissue specificity of gene expression, the smaller the Shannon entropy is. We then compared the Shannon entropy of genes interacting with and not interacting with D. The result showed that genes interacting with D had higher Shannon entropy than those not interacting with D (Supplementary Fig. 12a), contrary to expectation. In view of results in *Drosophila* that enhancers specific for housekeeping gene or developmental gene have different genomic distributions^40^, we divided D into two groups based on their location relative to gene: genic and intergenic D. Here genic D represented the D in gene body, intergenic D was D in intergenic region. Genes interacting with genic D had higher Shannon entropy than those interacting with intergenic D in both tissues (*p* <2.2e − 16; two-sided *t* test) (Fig. 5d), indicating that genes interacting with genic D prefer to be more constitutively expressed, as compared to those interacting with intergenic D. Furthermore, we wanted to check whether gene with D in its own gene body was even more constitutively expressed than gene interacting with genic D. Considering that we excluded the PETs within 10 kb, the interactions with distance shorter than 10 kb were abandoned. So the Shannon entropies of genes with D in their own gene bodies were calculated, regardless having chromatin interactions or not. The result indicated that genes with D in their gene bodies had even higher Shannon entropy than those interacting with genic D (*p* <2.2e − 16, two-sided *t* test), which suggested that these genes with D in gene bodies are more likely to be constitutive genes (Fig. 5d). Moreover, genes with D in gene bodies had much longer length than those interacting with genic or intergenic D (*p* <2.2e − 16, two-sided *t* test) (Supplementary Fig. 12b). Taken together, intergenic D was more likely to be associated with tissue-specific gene expression, while genic D tend to be associated with housekeeping gene expression. Moreover, the degree of tissue-specific expression of genes tended to increase, as the number of interacting intergenic D increased (Fig. 5e).

### The comparison of chromatin interactions between tissues

**Fig. 6:**
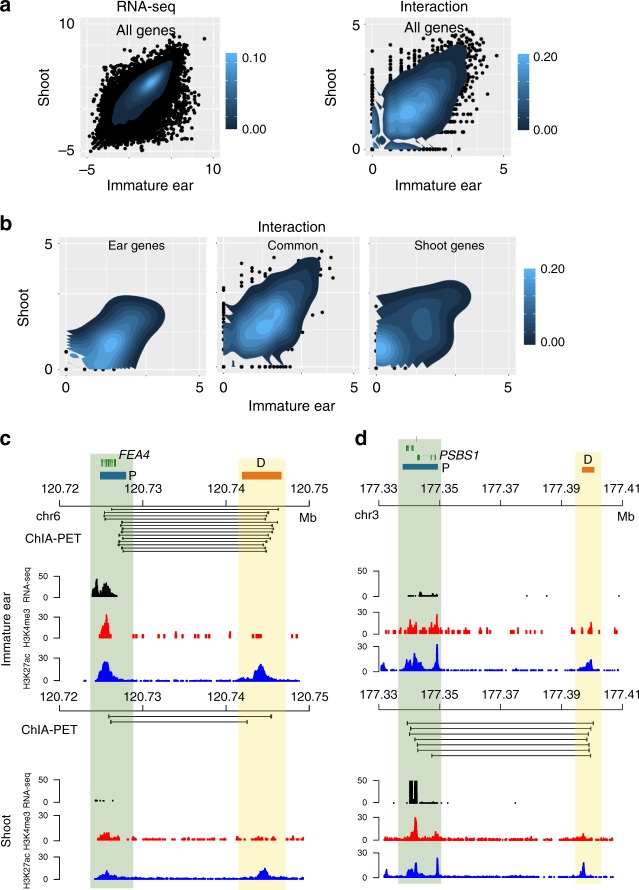
Tissue-specific chromatin interactions in immature ear and shoot. **a** The heat map of fragments per kilobase of transcript per million (FPKM) value (left panel) and chromatin interaction (right panel) of all genes in immature ear and shoot. **b** The heat map of chromatin interactions of immature ear-specific expressed genes (*p* = 0.006, two-sided *t* test), common genes, and shoot-specific expressed genes (*p* = 0.002, two-sided *t* test) in immature ear and shoot, respectively. **c** An example of immature ear-specific chromatin interaction. *FEA4* gene (highlighted in dark green) interacted with a distantly located (∼17 kb) distal PET peaks (D) (highlighted in yellow) in immature ear. In contrast, the *FEA4* gene in shoot was not expressed and had no interaction with that D. **d** An example of shoot-specific chromatin interaction. *PSBS1* gene (highlighted in dark green) interacted with a distantly located (∼58 kb) D (highlighted in yellow) in shoot. In contrast, the *PSBS1* gene in immature ear was not expressed and had no interaction with that D. The green rectangle represents the corresponding gene. The blue and orange rectangles indicate P and D, respectively

To further explore the relationship between chromatin structure and gene transcription, we identified tissue-specific expressed genes and tissue-specific chromatin interactions in immature ear and shoot (Fig. 6a, Methods). There were 286 immature ear-specific expressed genes, which expressed in immature ear but not expressed in shoot, and these genes showed stronger chromatin interactions in immature ear than in shoot. Similarly, 675 shoot-specific expressed genes, which were expressed in shoot but not expressed in immature ear, also showed stronger chromatin interactions in shoot than in immature ear (Fig. 6b). About 44% of chromatin interactions in immature ear and 49% in shoot were tissue specific (Supplementary Fig. 13a), and the remaining were common in both tissues. Moreover, compared to P–P interactions, most of which were shared in both tissues, tissue-specific interactions were enriched for P–D interactions (Supplementary Fig. 13b). Additionally, P–D interaction with distance of 100 kb–1 Mb were more dynamic between tissues than P–D interactions with distance shorter than 100 kb (Supplementary Fig. 13c, d), implying that proximal and distal regions involving these long-distance interactions can have much higher tissue-specificity.

Compared with all identified chromatin interactions, tissue-specific D showed enrichments in tissue-specific P–D interactions (Supplementary Fig. 13e). We identified 4299 ear-specific and 4515 shoot-specific P–D interactions (Supplementary Data 13–14). There were 3467 and 3804 D participating in immature ear-specific and shoot-specific P–D interactions, respectively. About 71% and 82% of these D were tissue-specific. Among the tissue-specific interactions identified, we captured many genes associated with tissue-specific functions. *FEA4*, one gene responsible for meristem size, is specifically expressed in the meristem in maize^41^. An immature ear-specific P–D interaction associated with *FEA4* was identified in our study (Fig. 6c). Another interesting example was an immature ear-specific expressed gene, *ZAG1*, the maize homolog of *Arabidopsis* floral identity gene *AGAMOUS*^42^. We found an immature ear-specific P–D interaction for *ZAG1*, with D ~220 kb downstream of *ZAG1* (Supplementary Fig. 13f). *PSBS1* encodes a subunit of photosystem II^43^, which had an extremely high transcription level in shoot, but very low expression in immature ear. This shoot-specific expressed gene was identified to interact with a shoot-specific P–D interaction, which may promote its expression in shoot (Fig. 6d).

### Chromatin interactions form functional networks

In maize, six well-characterized putative enhancers have been reported, namely the enhancers of the *B1*^44^, *TB1*^12^, and* P1*^45^, the putative enhancers DICE^15^ (for regulating the expression of *BX1*), *Vgt1*^14^ (for regulating the expression of *ZmRAP2*.*7*), and *KRN4*^13^ (for regulating the expression of *UB3*). In this study, we identified all the previously reported putative enhancers of *ZmRAP2*.*7*, *UB3*, *BX1*, and *TB1* (Supplementary Fig. 14, Fig. 1d, and Supplementary Fig. 4), and found many other interacting regions for these genes. The putative enhancer of *P1* was not detected due to the distance of the proximal enhancer to P-rr promoter (−1252 to +326)^45^ being shorter than 10 kb. Since *B1* gene was not assembled into the pseudochromosome in B73 RefGen_v4 reference, so it was excluded in our analysis.

**Fig. 7:**
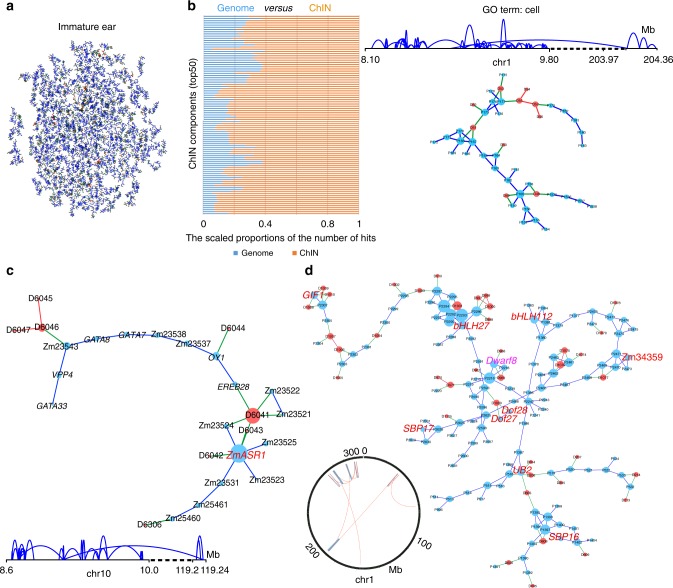
Functional communities of chromatin interactions in chromatin interaction network (ChINs). **a** All the ChINs identified in immature ear. Here only showed the ChINs with more than 30 nodes. **b** Enrichment of gene ontology (GO) terms in top 50 ChINs in immature ear and shoot. Only significant GO terms (false discovery rate (FDR) <0.05) are presented in the left panel. Enrichment of GO terms is represented as scaled proportions of the observed number of hits in a ChIN component (orange) vs. the expected number of hits in the genome (blue). The right panel showed a ChIN in immature ear, where all genes were enriched in cell function (GO term). **c** The ChIN for *ZmASR1* (in red) in shoot. The arc map in left bottom showed the region span of all chromatin interactions in this ChIN on genome. **d** The ChIN for *Dwarf8* (in pink), which regulates plant height and flowering time, in immature ear. There were other nine genes (*GIF1*, *UB2*, *bHLH27*, *bHLH212*, *DOF27*, *DOF28*, *SBP16*, *SBP17*, and Zm34359; in red) potentially associated with flowering in this ChIN. Circos map in the left bottom showed the region span of all chromatin interactions in this ChIN. Chromatin interactions with distance longer than 1 Mb are highlighted by orange arcs, others in blue. Ellipses in blue and red indicated proximal PET peaks (P) and distal PET peaks (D), respectively, in ChIA-PET datasets. P–P, P–D, and D–D interaction are presented in blue, green, and red edges in network. Source Data of Fig. 7d are provided as a Source Data file

Furthermore, interactions between P and D can interweave into complex networks. We identified 1655 and 1797 chromatin interaction networks (ChINs) with at least three chromatin interactions in immature ear and shoot, respectively (Fig. 7a, Supplementary Fig. 15a, and Supplementary Data 15–16). Moreover, ChINs, especially only considering P–P interactions, were conserved between two tissues. Out of 1085 P–P network components with at least 3 P–P interactions, 730 (67%) showed >65% overlap between immature ear and shoot (Supplementary Fig. 15b). Moreover, compared to genes with low interaction degree, genes with high interaction degree in ChINs had slight enrichment with GO term cell (GO:0005623) and cell part (GO:0044464) (Supplementary Fig. 15c, d).

Genes connected in the ChINs may be functionally related. To validate this, we analyzed the enrichment of GO terms for the top 50 network components. Among them, 23 showed enrichments in one or more GO terms (FDR <0.05) (Fig. 7b, Supplementary Data 17), suggesting that genes in the same network components were prone to function in related biological processes. Our results indicated that genes with the same network components could form functional communities, consistent with results reported in human^46^. For example, as for a ChIN on chromosome 1 in immature ear, genes in this ChIN showed GO enrichment in cell process (Fig. 7b). *Abscisic acid stress ripening1* (*ZmASR1*) is one of the highly expressed *ASR* gene family in maize, which affecting kernel yield under water-limited conditions and associating with leaf senescence^47,48^. As shown in Fig. 7c, the network component involving *ZmASR1* identified in our study consisted of 18 P and 8 D, a total of 28 chromatin interactions. Among all the 16 annotated genes in this network community, six genes (including *ZmASR1*, *EREB28*, Zm00001d023537, Zm0001d023538, Zm00001d023543, and Zm00001d025461) were related to abiotic or biotic stress responses. There were six genes (including *OY1*, *GATA7*, *GATA8*, *GATA33*, Zm00001d023524, and Zm00001d025459) that could respond to light stimulus or participate in chlorophyll biosynthetic process. The genomic span of functional communities with enriched GO terms on genome was 1 Mb in average, consisting with the size of TADs in maize genome^10^.

Genes related to complex agronomic traits are controlled by complex regulatory mechanism and can sometimes have pleiotropic effect. *Dwarf8*, an ortholog of *Rht* (*Reduced height*), *Slr1* (*Slender rice 1*), and *Arabidopsis GAI* (*Gibberellin Insensitive*), regulates the plant height in maize^49^. Many studies also showed that *Dwarf8* was associated with flowering time in maize^50,51^. We found that other nine genes in the ChIN of *Dwarf8* (Fig. 7d), including *GIF1*, *UB2*, *Dof27*, *Dof28*, *SBP16*, *SBP17*, *bHLH27*, *bHLH112*, and Zm00001d034359, were annotated to be associated with flower development and flowering time^34,52^. This ChIN component of *Dwarf8* may participate in genetic regulation of both plant height and flowering time. Additionally, the ChIN component of *Dwarf8* was the fourth biggest one of all the network components we identified, consisting of 204 chromatin interactions involving 115 P and 39 D, implying the complexity of regulation related to flowering and plant height in maize (Fig. 7d).

## Discussion

As well demonstrated in animal systems, enhancers have played crucial roles in the transcriptional regulation of genes. The identification and function inference of enhancers are of importance for understanding the genetic regulation of genes related to human diseases as well as complex agronomics traits of crop plants. Having adopted the ChIA-PET methodology, we obtained genome-wide interaction map of DRs and their corresponding proximal regulatory regions in maize. Similar to what was estimated in human and mouse, we found that about 40% DRs chose to skip individual adjacent genes to regulate genes in a long-range manner. There are about 2–3% of genes that have interacting DRs 1 Mb away. The information resulted from this study will undoubtedly be useful for our further understanding the regulation of gene expression in a three-dimensional context in maize and other plants.

Our results showed that about 50% DRs were located in the intergenic regions of genome, confirming that these intergenic regions are an important source of regulatory elements. More interestingly, we found that about 30% of DRs were located in TE regions, a number that was consistent with two other reports in maize^19,24^. Similar results were also found in human, as there were 20–30% TEs that had tissue-specific enhancer activity. These TEs displayed hypo-methylation and were occupied by p300 and H3K4me1^53^. Our ChIA-PET data showed that many of these DRs with TE-like sequences were indeed able to interact with promoters, indicating that these TE-like DRs are functionally active. As pointed in a previous study^24^, these TE sequences were highly decayed. Since we only used uniquely mapped reads for analysis, the number of TE sequences with DR function must be underestimated. In maize, about 80% of genome belongs to sequences of various TEs. For a long time, these TE elements were considered as junk sequences. How these TE-like sequences are recruited to be functional DRs and how they are targeted for demethylation are interesting questions for future studies. In addition, a study in humans showed that 2–3% of coding-gene promoters functioned as bona fide enhancers in a given cell line^54^. We also founded strong signals of H3K27ac in many proximal regulatory regions, implying that many regulatory elements could be in short distance to their corresponding promoters.

Our results showed that gene interacting with intergenic D was prone to be tissue-specific gene, while gene interacting with genic D (especially D in its own gene body) was likely to be a constitutive gene. This implied that a constitutive gene may often form relatively stable self-loop between its proximal regions and DRs, which ensured the high expression level needed all the time. In human, enhancers of housekeeping genes were active across different cell types, while developmental enhancers showed strong cell-type specificity^40^. Tissue-specific genes only need to be expressed in certain tissues or time points, and they were more dynamic. To gain the dynamics, they may need to change the combination of regulatory elements, which were located far away from them, and the corresponding dynamic chromatin interactions may provide the structure basis to achieve this goal. The chromatin connectivity pattern between enhancers and promoters is not always in monogamy, indicating the existence of highly complex and cooperative regulation mechanism. Studies in humans and mouse showed that 60–70% of promoters interacted with only one enhancer and about 90% of putative enhancers interacted with only one gene. In maize, we found that 75–80% of P interacted with only one D. A previous study in mouse showed that cell-type-specific genes were more likely to be monogamous than constitutive genes. This phenomenon of P interacting with multiple D may provide the structural basis of functional redundancy or multiple ways for gene activation. At the same time, 70–75% of D interacted with only one P in our current study. Genes interacting with the same D tended to be co-expressed, which implied that they may contribute to related pathways or agronomic traits. Taken together, the complex interaction pattern between D and P could be a general mechanism throughout the genome, forming synergistic and dynamic regulatory networks.

Genes interacting with D did not absolutely mean to have high expression levels. A previous study in human fibroblasts showed that enhancer–promoter interactions already existed in each cell type before the binding of transcription factors, and this kind of interaction underwent relatively few alterations during transient transcriptional activation^5^. Further research indicated that the construction of chromatin interaction and gene expression was not always synchronous during cell differentiation in *Drosophila* and humans^36,37^. There were two classes of enhancer–promoter contacts, gained and stable type, during human epidermal differentiation^37^.

The chromatin interactions between proximal regions and DRs can further interweave into complex networks. The putative enhancers of *TB1*, *UB3*, *BX1*, and *ZmRAP2*.*7* were all validated in our datasets. These genetically identified enhancers have important regulatory roles for their corresponding agronomic traits. A large number of DRs and their interacting genes identified in our study provide a sound resource to be used to understand the underlying regulatory mechanism of these genes. ChINs identified in our study is therefore of importance for understanding the genetic regulation of genes related to complex agronomic traits.

The network information from our study can help to find relevant candidate genes for certain agronomic traits. Genes from the same ChIN showed enrichments in one or more GO terms, consistent with the results in human^46^. Genome-wide association studies have revealed many loci for complex traits, which can sometimes locate in non-coding regions. Using the chromatin interaction data^55,56^, candidate target genes interacting with those regulatory elements can possibly be identified. In addition, causal genes for important traits can be identified through a network-based gene finding strategy using our ChIN data^57^.

Besides, genes were systematically distributed in the organized chromatin networks. In our current study, genes with high interaction degree, which means that they serve as hubs of network, showed enrichments for genes related to key cell functions. Studies in human showed that genes in network hubs were often lethal^46^. The disease-associated SNPs showed enrichments at nodes of lower interaction degree, while node genes with high interaction degrees were not found to associate with such SNPs^46^. Moreover, the hub nodes were often hyperacetylated for efficient DNA repair, which generally located to early replicating domains to reduce the possibility of genetic mutation upon replication^46^. It is reasonable to assume that candidate genes that are responsible for small quantitative changes of agronomic traits are more likely those of low degree of interactions. High-resolution chromatin interaction maps are thus useful to explore transcriptional regulatory mechanisms and its implication for agricultural applications.

## Methods

### Material preparation of shoot and immature ear

The seeds of maize inbred line B73 were planted in our in-house growth chamber in the condition of 25 °C for 15 h under light and 20 °C for 9 h in the dark. After 14 days, the aerial tissues were harvested. As for immature ear, B73 inbred lines were planted in the field in Beijing in May 2016. Immature ears of ~5 cm in length were harvested. The two tissues were cut into small species and then crosslinked with EGS (ethylene glycol bis(succinimidyl succinate)) and formaldehyde sequentially as reported^58^. Procedures for collecting and crosslinking these two tissues were described in details in Supplementary Methods.

### ChIP-seq library construction

Crosslinked shoot and immature ear were subjected to nuclei extraction, chromatin sonication, and immunoprecipitation with H3K4me3 antibody (1 μg/μl, Abcam, cat. no. ab8580) and H3K27ac antibody (1 μg/μl, Abcam, cat. no. ab4729), respectively, as reported^59^. Detailed procedures for constructing ChIP-seq libraries were described in Supplementary Methods.

### ChIA-PET library construction

Following the ChIP procedure, the ChIPed chromatins were subjected to end repair, dA tailing, and proximity ligation with the given bridge linkers, followed by lambda and Exo I treatment according to the long-read ChIA-PET method^7,58^. Detailed procedures for constructing ChIA-PET libraries were described in Supplementary Methods.

### 4C-seq library construction and data analysis

We modified an earlier 4C-seq process^60^ with in-nucleus proximity ligation and a 4 bp cutter (*Dpn*II). All primer sequences and viewpoints information used in 4C-seq were listed in Supplementary Table 4. Detailed procedures for constructing 4C-seq libraries were described in Supplementary Methods. The 4C-ker software^61^ with default parameters was used to identify chromatin interactions with viewpoints in 4C experiments. Figure 1d and Supplementary Fig. 4 showed the average of normalized 4C signal between two replicates. Interactions identified in both replicates in 4C data were reserved.

### RNA-seq library construction and gene expression analysis

Total RNA of immature ear were extracted by TRIzol method. RNA-seq libraries were constructed following the VAHTS mRNA-seq v2 Library Prep Kit for Illumina® (NR601) of Vazyme Company. The raw reads of RNA-seq data were aligned to maize B73 reference (RefGen_v4) using TopHat^62^, and only uniquely mapped reads were used to calculate the FPKM for each gene by Cufflinks software^63^. The RNA-seq data of shoot were used published data^64^.

### Identification of H3K4me3 and H3K27ac peaks

The raw reads of ChIP-seq data were aligned to the B73 reference genome (RefGen_v4) using the bowtie2 software^65^. The H3K4me3 and H3K27ac peaks in immature ear and shoot were identified using MACS^66^ with the parameter: *p* ≤ 1 × 10^−5^. The statistical summary of ChIP-seq datasets was in Supplementary Table 2.

### Identification of chromatin interactions

The pipeline for identification of chromatin interaction was shown in Supplementary Fig. 2, which was similar to previous reports^7,67^ with modifications. The Bowtie software^68^, with parameters -m 1 -k 1 -n 2 -S -p 6, was used in mapping reads to B73 reference genome (RefGen_v4). Only reads with quality bigger than 30 in bam files were saved. The reads of unique PETs were used to call PET peaks by the MACS^66^ software with parameters: –*p*value 1e − 09, –nolambda, –nomodel, –keep-dup = 2. To identify long-range chromatin interactions, we filtered PETs with genomic span <10 kb, which were classified as self-ligation PETs. To identify high-confidence chromatin interactions, a statistical model based on the hypergeometric distribution was used to calculate the probability of seeing at least the observed number of PETs between two PET peaks (*P* [*X* ≥ *x*]). For each candidate chromatin interaction, we calculated (1) the number of PETs overlapping with each PET peak, (2) the number of PETs linking the two PETs peaks, and (3) the total number of PETs for all candidate chromatin interactions. The probability was calculated by the *Phyper* function in R software. Then, the *p* value was adjusted by the Benjamini–Hochberg method for multiple comparisons. Finally, chromatin interactions with at least three PETs and FDR <0.01 were defined as high-confidence interactions in each sample.

### Calculation of gene expression correlation

To calculate co-expression level for gene pairs with chromatin interactions, we investigated the expression patterns of gene pairs by calculating the Pearson’s correlation coefficient in the collected RNA-seq dataset as reported^38^, consisting of 53 different seeds and 25 non-seed samples in maize. Only proximal PET peaks with only one gene promoter were used. To eliminate the effect of distance for gene pairs in control, we randomly shift the chromatin interaction 100 times in the same chromosome to generate lots of random loops with the same distance. Random loops linking two different gene promoter regions were reserved. Then, we filtered random loops without H3K4me3/H3K27ac peaks or with low H3K4me3/H3K27ac ChIP-seq signal.

### Calculation of Shannon entropy

The formula for the calculation of Shannon entropy was adapted from previous reports^39^. Given that there are *N* tissues, we defined *p*_*t*|*g*_ as the relative expression level of gene *g* in tissue *t*:

1 $$p_{t|g} = {w}_{g,t}/\sum _{{\mathbf{1}} \le t \le N}{w}_{g,t}$$

Here, *w*_*g*,*t*_ represents the expression level of gene *g* in the tissue *t*.

We defined *H*_*g*_ as Shannon entropy:

2 $$H_{g} = \sum _{{\bf{1}} \le t \le N} - {p}_{t|g}{\mathrm{log}}_2\left( {p_{t|g}} \right)$$

*H*_*g*_ ranges from 0 to log_2_(*N*).

The higher the Shannon entropy is, the more likely the gene is to be constitutively expressed, and vice versa. To obtain the expression level of genes in different tissues, we used the reported RNA-seq dataset^38^, which consists of 53 different seeds and 25 non-seed samples in maize, for calculation of the Shannon entropy of each gene.

### The analysis of tissue-specific chromatin interactions

To analyze the association between gene expression and chromatin interaction in different tissues, we identified genes with changed transcriptional state between immature ear and shoot, which were named as tissue-specific expressed gene between two tissues. A stringent criterion was applied to reduce noise. If one gene with FPKM = 0 in one tissue, and had FPKM ≥ 2 in another tissue, then the gene was identified as a tissue-specific expressed gene in the second tissue. We identified 286 immature ear-specific expressed genes and 675 shoot-specific expressed genes. For each tissue-specific expressed gene, we got the number of PETs interacting with its promoter region in two tissues. Figure 6b showed the smoothed scatterplot of the log-transformed PET counts at the same genes from two tissues. Genes with no interacting PETs in both tissues were removed. To identify tissue-specific chromatin interactions, we combined chromatin interactions identified in H3K4me3-ChIA-PET and H3K27ac-ChIA-PET libraries in the same tissue. If one chromatin interaction existed in one tissue and did not exist in another tissue, we identified this interaction as the first tissue-specific chromatin interaction. Moreover, 99% of tissue-specific chromatin interactions we identified had H3K4me3 or H3K27ac anchor sites in both tissues.

### ChIN analysis and visualization

The igraph library in R software was used for the construction of chromatin network components. The nodes in ChINs present PET peaks in chromatin interactions. Each edge in ChINs presents corresponding chromatin interaction. All chromatin interactions in each tissue were used to construct corresponding ChINs. Nodes that interacted directly or indirectly consist of a chromatin network component. We used the Cytoscape^69^ software to visualize ChIN (https://cytoscape.org/).

### GO analysis

GO analysis was performed using Agrigo^70^ (http://bioinfo.cau.edu.cn/agriGO/index.php).

### Statistical analyses

All the statistical tests were performed using R software (https://www.r-project.org/).

### Reporting summary

Further information on research design is available in the Nature Research Reporting Summary linked to this article.

## Supplementary information

Supplementary Information

Peer Review

Reporting Summary

Description of Additional Supplementary Files

Supplementary Data 1

Supplementary Data 2

Supplementary Data 3

Supplementary Data 4

Supplementary Data 5

Supplementary Data 6

Supplementary Data 7

Supplementary Data 8

Supplementary Data 9

Supplementary Data 10

Supplementary Data 11

Supplementary Data 12

Supplementary Data 13

Supplementary Data 14

Supplementary Data 15

Supplementary Data 16

Supplementary Data 17

Source data

## Data Availability

The authors declare that all data supporting the findings of this study are available within the article and its Supplementary Information files or from the corresponding author upon reasonable request. A reporting summary for this Article is available as a Supplementary Information file. All ChIA-PET, ChIP-seq, 4C, and RNA-seq data were deposited into NCBI SRA with accession number SRP162618, SRP158640, SRP154106, SRP158820, respectively. All published accessions used in the data analyses are listed in Supplementary Table 6. The source data underlying Figs 1d, 4a, b, 5d, e, and 7d and Supplementary Figs 4–6, 10a and 10b are provided as a Source Data file.

## Code availability

Source code is available in the GitHub repository https://github.com/liencau/ChIA-PET-analysis.
