# Supplementary material for: Long-range interactions between proximal and distal regulatory regions in maize
Source: Nat Commun. 2019 Jun 14;10:2633. doi: 10.1038/s41467-019-10603-4 (PMC6572780; doi:10.1038/s41467-019-10603-4)
Supplement: Supplementary file 1 — Supplementary Information [file 41467_2019_10603_MOESM1_ESM.pdf]

# Long-range interactions between proximal and distal regulatory regions in maize

Li *et al.*

# Supplementary Methods

## Material preparation of shoot and immature ear

The seeds of maize inbred line B73 were planted in our in-house growth chamber in the condition of 25 °C for15h under light and 20 °C for 9h in the dark. After 14 days, the tissues above ground were harvested and cut into small species of 1cm$^2$ around, then immersed in 1.5 mM EGS solution (Thermo, cat. No. 21565) and subjected to vacuum infiltration for 20min,followed by 10min vacuum infiltration of 1% formaldehyde (Merck, cat. No. F8775-500ML). To quench the remaining EGS and formaldehyde, glycine (Merck, cat. No. V900144) with a final concentration of 0.15 mM was added and vacuum infiltrated for 5min. Shoots were washed thorough with double distilled water five times, and the remaining water removed. The crosslinked shoot was frozen with liquid nitrogen and stored at -80 °C. As for immature ear, seeds of B73 inbred line were planted in the Shangzhuang Experimental Station affiliated to China Agricultural University in May, 2016. Immature ears of ~5cm in length were harvested and cut into small packs, and then crosslinked as mentioned above.

## ChIP-seq library construction

The ChIP-seq libraries for shoot and immature ear were constructed as described[1] with a few modifications. Crosslinked shoot (4g) or immature ear (0.2g) was ground into fine powder for each experiment. The powder was suspended in 40 ml Nuclear Extraction Buffer I (10 mM Tris pH 8.0, 400 mM Sucrose, 10 mM Na-butyrate, 0.1 mM PMSF, 5 mM Beta-Mercaptoethanol) with newly added 1×roteinase Inhibitors (Roche, cat. No. 04693159001), vortexed briefly, and added 500ul 20% Triton for removal of chloroplast or other cell debris as more as possible, and then incubated in ice for 15min on a rotator with a gentle speed of 110 rpm. Subsequently, the suspended buffer was infiltrated into a new 50 ml BD tube with four-layer Mira cloth (Millipore, cat. No. 475855-1R) and centrifuged at 2880 g and 4 °C for 20min. The supernatant was carefully poured and the eluate was

resuspended again with another 10 ml Nuclear Extraction Buffer I, centrifuged at 2880g and 4 °C for another 10min. The pellets were suspended with 1 ml Nuclear Extraction Buffer II (10 mM Tris pH 8.0, 250 mM Sucrose, 10 mM Na-butyrate, 10 mM $MgCl_2$, 1% v/v Triton X-100, 0.1 mM PMSF, 5 mM Beta-Mercaptoethanol, 1×Proteinase Inhibitors) and centrifuged at 12,000g and 4 °C for 10min. The supernatant was carefully decanted, and suspended the pellet in 300 μl Nuclear Extraction Buffer III (10 mM Tris pH 8.0, 1.7M Sucrose, 10 mM Na-butyrate, 2 mM $MgCl_2$, 0.15% v/v Triton X-100, 0.1 mM PMSF, 5 mM Beta-Mercaptoethanol, 1×Proteinase Inhibitors) and transferred into a 2 ml tube with 1.5 ml Nuclear Extraction Buffer III as a cushion, followed by centrifugation at 16,100g and 4 °C for 1h. The resulting pellets were suspended in 300 μl Nuclear Lysis Buffer (50 mM Tris pH 8.0, 10 mM EDTA, 0.4% w/v SDS, 0.1 mM PMSF, 1×Proteinase Inhibitors), and subjected to our in-house Bioruptor sonicator with about 5-cycle of 30s-on and 30s-off at the HIGH level, to obtain chromatin fragments with its bound DNA length of 200~700bp. After sonication, we took 10 μl sonicated product into another new 1.5 ml tube and centrifuged it at top speed for 5min. Supernatant was transferred into another tube, then added 2 μl RNase A and 2 μl Proteinase K, mixed well and incubated at 55 °C for 20min. We checked the DNA length distribution on 1.5% agarose gel. The remaining sonicated chromatins were centrifuged at top speed for 10min at 4 °C, and the supernatant was transferred into a new 1.5 ml tube for another 15min centrifugation at top speed at 4 °C. Supernatant was transferred into a 2 ml tube with 1,660 μl ChIP Dilution Buffer (50 mM Tris pH 8.0, 1 mM EDTA, 0.1% v/v Triton X-100, 150 mM NaCl, 10 μg/ml BSA) and 40 μl pre-washed ChIP-grade Protein A/G beads (Thermo, cat. No. 26162), and pre-cleared on an IntelliMixer with the setting of F1 and12rpm for 2h at 4 °C. After pre-clearing, beads were retrieved with a magnetic stand. All the solution was divided into three aliquots, added additional 400 μl ChIP Dilution Buffer each, 40 μl ChIP-grade Protein A/G beads and 5 μg H3K4me3 (Abcam, 1 μg/μl, cat. No. ab8580) or 5 μg H3K27ac (Abcam, 1 μg/μl, cat. No. ab4729) antibody for each aliquot. After overnight incubation, the chromatin-antibody-bead complex was washed sequentially once with 1 ml Low Salt Washing Buffer (20 mM Tris pH 8.0, 2 mM EDTA, 0.1% w/v SDS, 1% v/v Triton X-100, 150 mM NaCl), 1 ml High Salt

Washing Buffer (20 mM Tris pH 8.0, 2 mM EDTA, 0.1% w/v SDS, 1% v/v Triton X-100, 500 mM NaCl) and 1 ml LiCl Washing Buffer (20 mM Tris pH 8.0, 1 mM EDTA, 1% v/v NP-40, 1% w/v Na-deoxycholate, 250 mM LiCl), followed by washing using 1×TE pH8.0 (10 mM Tris pH 8.0, 1 mM EDTA) twice. We resuspended the complex in 1 ml 1×TE pH8.0 and took 100 μl for real-time PCR in the in-house ABI 7500 Real Time PCR System to check the enrichment. Only delta-delta Ct values larger than three were retained. Two sets of primers of positive control were selected for the real-time PCR enrichment validation for each histone modification. For H3K4me3, two sets of primers of positive control were chosen for enrichment validation, *actin*_exon2[1]and in-house designed GAPDH_upstream_1kb. For H3K27ac, two sets of primers of positive control were used as reported[2]. The primers of negative control, *Copia*[1], were selected for both histone modifications. For ChIP-seq library construction, we subjected the remaining 900 μl ChIPed complex to a magnetic stand, removed the solution and eluted the beads with 200 μl ChIP Elution Buffer (10 mM Tris pH 8.0, 1 mM EDTA, 1% SDS) in a ThermoMixer for 15min at 65 °C and 900 rpm. We retrieved the beads, pooled the 200 μl eluate with 400 μl 1×TE pH8.0, and mixed it with 10 μl Proteinase K (Thermo, cat. No. EO6491) for overnight digestion at 55 °C. Fragments released from the ChIPed protein-DNA complex were precipitated with phenol:chloroform:isopropanol (25:24:1), washed with 75% ethanol and dried with in-house Eppendorf Concentrator. The resulting ChIPed DNA were resolved in ddH$_2$O and subjected to conventional Illumina library construction. The ChIP-seq libraries for both histone modifications were subjected to the X-ten sequencer with 2×150bp strategy in the Beijing AnnoRoad Company. Primers used for qPCR in ChIP and ChIA-PET were listed in **Supplementary Table 4**.

**ChIA-PET library construction**

The ChIA-PET libraries for shoot and immature ear were performed followed publications[3,4]. After proper rounds of the ChIP process described above, the ChIPed DNA

should be 500~1000ng as the starting quantity for each ChIA-PET. First, the ChIPed chromatin on-bead was subjected to end-repair with 70 µl 10×T4 DNA polymerase buffer, 7 µl 10 mM dNTP, 615.8 µl double distilled water and 7.2 µl T4 DNA Polymerase, incubated at 37 °C for 1h on the IntelliMixer with the setting at F8 and 30rpm. The beads were washed with 1 ml pre-cold ChIA-PET Washing Buffer (10 mM Tris pH 8.0, 1 mM EDTA, 500 mM NaCl) thrice. Second, dA-tailing was performed following end-repaired chromatin with 70 µl 10×NEBuffer 2.0 (NEB,cat. No. B7002S), 7 µl 10 mM dATP, 617 µl double distilled water and 7 µl Klenow, 3'-5' exo-(NEB,cat. No. M0212L). We incubated it at 37 °C for 1h on the IntelliMixer with the setting at F8 and 30 rpm. The beads were washed with 1 ml pre-cold ChIA-PET Washing Buffer thrice. Third, proximity ligations were performed toward the dA-tailing chromatin, with 4 µl 200ng/µl bridge-linker (forward strand: 5'-/5Phos/CGCGATATC/iBIOdT/TATCTGACT-3', and reverse strand: 5'-/5Phos/GTCAGATAAGATATCGCGT-3′), 280 µl 5×T4 DNA Ligase Buffer with PEG (Invitrogen, cat. No. 46300018), and 1,110 µl double-distilled water. When mixed well with the on-bead chromatin, the mixture above was then added 6 µl high-concentration 2,000U/µl T4 DNA Ligase (NEB, cat. No. M2200S) and inverted quickly. The reaction was incubated at 16 °C overnight on the IntelliMixer with the setting at F8 and 30 rpm. On the next day, fourth, the beads were washed with 1 ml pre-cold ChIA-PET Washing Buffer thrice. The beads were mixed with 70 µl 10×Lambda Exonuclease Buffer, 618 µl double distilled water, 6 µl Lambda Exonuclease (NEB, cat. No. M0262L) and 6 µl Exonuclease I (NEB, cat. No. M0293S), to remove unligated fragments. The reaction was incubated at 37 °C for 1h on the IntelliMixer with the setting at F8 and 50rpm. Then the beads were retrieved on magnetic rack, and the Exonuclease Mixture removed. We added 200 µl ChIP Elution Buffer, and incubated it at 65 °C for 15min with the ThermoMixer at900rpm. After that, the reaction was put on the magnetic rack and the supernatant was transferred to a new 1.5 ml tube, added another 200 µl ChIP Elution Buffer, mixed with beads and incubated at 65 °C for 15min further. The beads were retrieved with magnetic rack and transferred and mixed with previous 200 µl eluate, now 400 µl together. Another 200 µl double-distilled

water was added as well as 10 µl 20 mg/mL Proteinase K, mixed well and incubated at 55 °C overnight or at least 8h. For DNA extraction and precipitation, 600 µl PCI (phenol:chloroform:isopropanol, 25:24:1) were added at RT (room temperature) and vortexed thoroughly for at least 10 seconds, transferred to a Phase-lock Gel tube (China TIANGEN Company, cat. No. WM5-2302830) and centrifuged at 13,000 g for 5min at RT. The supernatant was transferred into a new 1.5 ml tube with 2 µl Glycoblue, 60 µl 3M Sodium Acetate pH 5.5 and 600 µl isopropanol. The mixture was vortexed completely and frozen in liquid nitrogen. The tube was centrifuged at top speed for 30min at 4 °C and the pellets were washed with newly prepared 75% ethanol twice. After take-out the majority of ethanol in the tube, we centrifuged briefly to collect the remaining ethanol at tube lid and tube wall and further removed them, incubated at 60 °C for 3min with the tube lid open and dissolved the pellet with 20 µl 1×TE, pH 8.0. Qubit 2.0 was used to accurately determine the pellet concentration for subsequent Tn5 treatment.

To fragment and capture interacting sequences, we used the Tn5 transposase (Nanjing Vazyme Company of China, cat. No. TD501). For each reaction, 50ng DNA was aliquoted with 10 µl 5×TTBL Buffer, 5 µl Tn5. And appropriate double-distilled water were mixed well and incubated at 55 °C for exact 10min. Multiple reactions could be performed in parallel for the total DNA from each ChIA-PET to enable maximum library complexity. After Tn5 treatment, we used Zymo (cat. No. D4013) or Qiaquick PCR Purification Kit (cat. No. 28106) for DNA purification. The resulting DNA was dissolved in 50 µl double-distilled water.

To immobilize biotin-embedded Tn5-treated DNA to Streptavidin beads, we took 30 µl pre-suspended M280 Dynabeads (Thermo, cat. No. 11205D) and placed at RT for at least half an hour. The beads were washed twice with 150 µl 2×Binding&Washing Buffer (2×BW, including 10 mM Tris pH 8.0, 1 mM EDTA and 2M NaCl). The iBlock Buffer (in 10 ml solution, add 3 ml 20×SSC, 0.05g skimmed milk powder and appropriate water) was prepared at this step. We mixed the beads with 100 µl iBlock Buffer and incubated at RT for 45min on the IntelliMixer with the setting at UU and 50 rpm. During the incubation, we

prepared 500ng sonicated-sheared salmon sperm DNA in 50 μl double-distilled water with 50 μl 2×BW and mixed well. After iBlock Buffer incubation, we collected the beads and washed twice with 200 μl 1×Binding &Washing Buffer (1×BW, including 5 mM Tris pH 8.0, 0.5 mM EDTA and 1M NaCl), then added 100 μl salmon DNA/B&W mixture, mixed well and incubated at RT for 30 min with UU and50rpm. After this blocking step, the beads were washed twice with 200 μl 1×BW. The beads were mixed with the 50 μl Tn5-treated DNA and 50 μl 1×BW, incubated at RT for 45min on the IntelliMixer at UU and 50 rpm. Subsequently, we retrieved the beads and discarded the supernatant, washed beads five times with 500 μl 2×SSC/0.5%SDS (in 10 ml solution, add 1 ml 20×SSC, 0.5 ml 10% SDS and 8.5 ml water) pre-warmed at 42 °C, followed by two rounds of washing with 500 μl 1×BW. Finally, the beads were resuspended in 25 μl double-distilled water.

For PCR amplification of limited cycles, we adopted the 50 μl PCR condition of Vazyme Company. We mixed24 μl on-bead DNA, 10 μl 5×TAB Buffer, 5 μl PPM,5 μl N5 primer and 5 μl N7 primer, pipetted up and down well, finally added 1 μl TAE amplification enzyme and mixed well. The PCR was performed on the Thermal Cycler with the program of (1) 72 °C for 3min, (2) 98 °C for 30s, (3)10-cycle of 98 °C for 10s, 60 °C for 30s, 72 °C for 1min, (4) 72 °C for 5min and hold at 4 °C. We used 5 μl of the PCR products to check the smear size on the 1.5% agarose gel. We added 5 μl water to the remaining product and mixed well, purified it with two round uses of VAHTS DNA Clean Beads (Nanjing Vazyme Company of China, cat. No. N411-03-AA), first round with 0.6×(30 μl) and second round 0.15×(7.5 μl) to collect the 300~700bp PCR products. The beads were eluted in 20 μl 1×TE, pH 8.0. Finally, the ChIA-PET libraries were quality checked and subjected to the X-ten sequencer with 2×150bp strategy in the Beijing AnnoRoad Company.

**4C-seq**

The 4C library were constructed as reported[5,6] and we improved it with modifications[7,8] via 4bp-cutter digestion and proximity ligation, to enable relatively complete chromatin digestion for better resolution and cost reduction. We chose the combination of restriction enzymes, DpnII and NlaIII both as 4bp-cutters, for the two maize tissues. Briefly, B73 shoot (4 g) or immature ear (0.2 g) was taken as started for nuclei extraction. The resulting pellet was rinsed with 600 µl 1.2×NEBuffer3.1 (NEB, cat. No. B7203S) and centrifuged for 5min at 1,900g. The supernatant was discarded and the pellet was dissolved in 500 µl 1.2×NEBuffer 3.1 with the addition of 15 µl 10% SDS (0.3% at final). We incubated for 10min at 62 °C with no shake or vortex so as to premeabilize the nuclei membrane and inactivate the endogenous enzymes. When it cooled down to room temperature, 50 µl 20% Triton X-100 was added (2% at final) and mixed gently to quench the remaining SDS for 15min at 37 °C, inverted gently up and down every five minutes. Subsequently, 7.5 µl 50U/µl DpnII (NEB, cat. No. R0543M) were added and incubated at 37 °C and 900rpm in a ThermoMixer for 2h. After that, DpnII was inactivated for 20min at 62 °C. For proximity ligation after DpnII inactivation, the ligation mixture was prepared with 710 µl double-distilled water, 150 µl 10×T4 DNA Ligase Buffer (NEB, cat. No. B0202S), 40 µl 20% Triton X-100, 15 µl 10 mg/ml BSA and 10 µl 400 U/µl T4 DNA ligase (NEB, cat. No. M0202L). This mixture was added and mixed gently, incubated at room temperature for 4h. When the reaction was over, we centrifuged for 5min at 1,900g and discarded the supernatant. The pellet was dissolved with 400 µl ChIP Elution Buffer (10 mM Tris pH 8.0, 1 mM EDTA, 1% SDS) and added 10 µl 20 mg/ml Proteinase K, incubated at 55 °C overnight. Subsequently, the proximity ligated DNA was retrieved by the phenol:chloroform:isopropanol (25:24:1) precipitation and 75% ethanol washing, and eluted in 100 µl 1×TE pH 8.0. The DNA concentration was quantified with Invitrogen Qubit 2.0 and aliquoted about 20 µg for subsequent second digestion by NlaIII with 20 µl 10×CutSmart Buffer (NEB, cat. No. B7204S) and 8 µl 10 U/µl NlaIII (NEB, cat. No. R0125L), and appropriate double-distilled water to a final volume of 200 µl. We incubated the mixture for at least 1h at 37 °C and proceeded to inactivate the enzyme for 20min at

65 °C. We checked the digested smear on 1.5% agarose gel to ensure that the majority of the smear was shorter than 700 bp. We directly transferred all the remaining reaction volume to a 50 ml BD tube with 1.4 ml 10×T4 DNA Ligase Buffer and 10 µl 400U/µl T4 DNA Ligase with appropriate water to a total volume of 14 ml. The mixture was incubated for at least 4h at 16 °C followed by DNA precipitation and 75% ethanol washing. The resulting DNA was eluted in 100 µl 1×TE pH 8.0. The 4C primers for individual viewpoints were listed in the **Supplementary Table 4**.

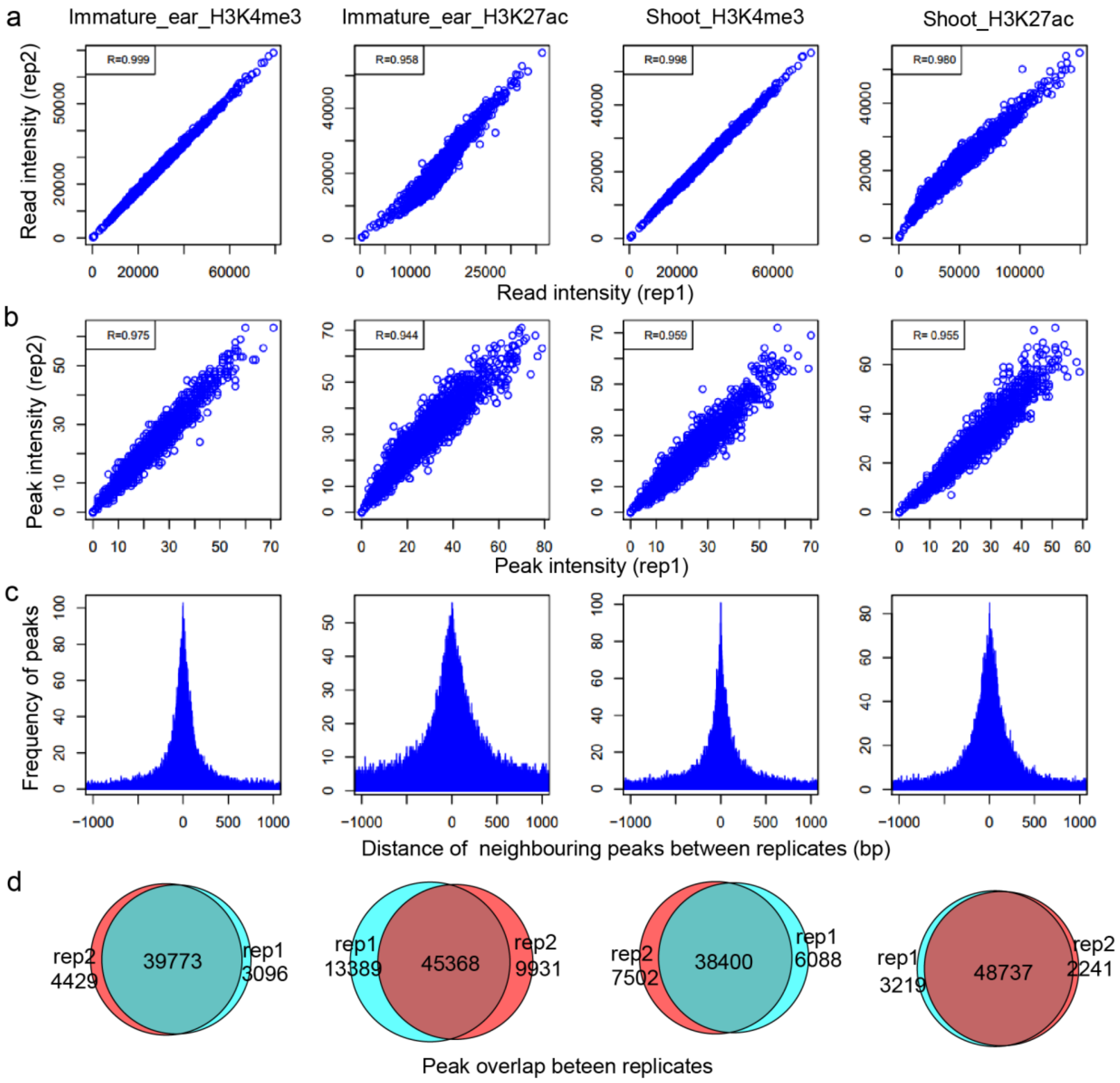


**Supplementary Figure 1. Reproducibility analysis between replicates.**

(a and b) Correlation analysis of read intensity and peak intensity between replicates in each sample. Read intensity or peak intensity was calculated against the number of reads or peaks in bin of 1Mb. *Pearson's correlation coefficients* (R) are shown at the left top corner.

(c) Histogram of distances between neighboring peaks from replicates in each sample.

(d) Venn diagram of PET peaks overlap between replicates in each sample.

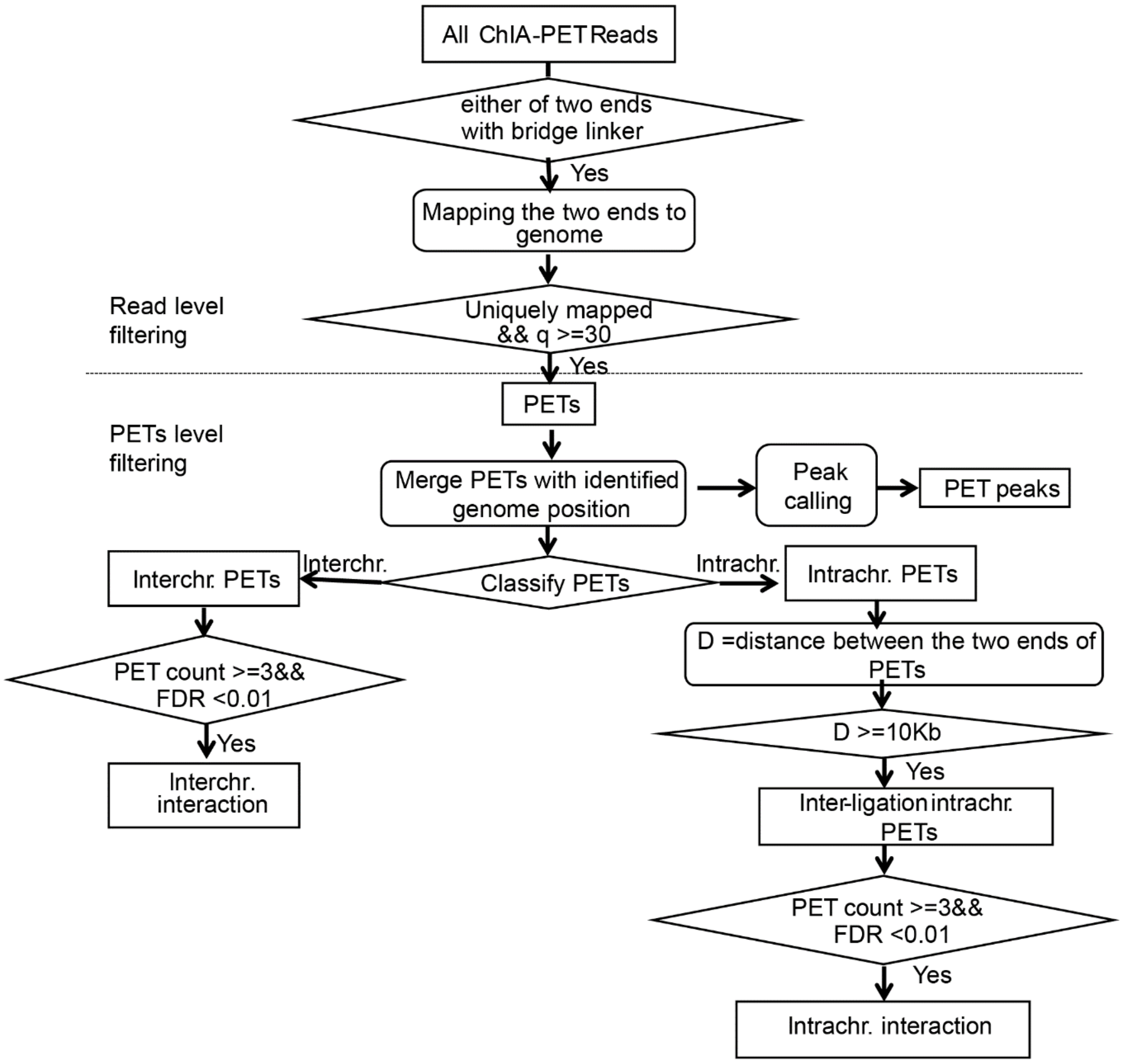


**Supplementary Figure 2. The workflow for identification of chromatin interactions.**

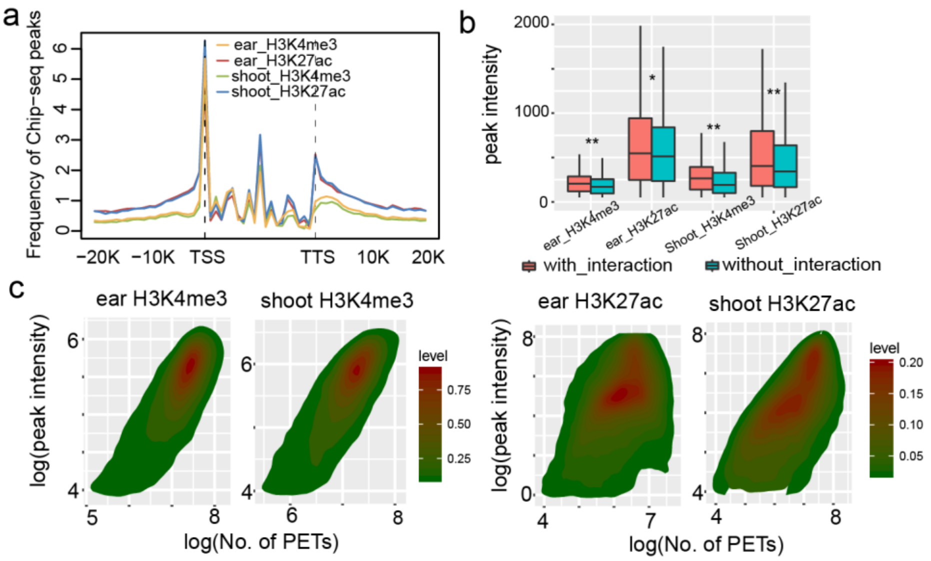


**Supplementary Figure 3. Identification of H3K4me3/H3K27ac peaks and chromatin interactions.**

(a) H3K27ac and H3K4me3 peak profile from ChIP-Seq datasets aroundgene body.

(b) The comparison of peak intensity for H3K27ac and H3K4me3 peaks with/without interactions in each sample. Here interactions used both intrachr. and interchr. interactions (***p* -value<0.01,**p*-value< 0.05, two-sided t-test).In the box plot, the central rectangle spans the first quartile to the third quartile (the interquartile range). A segment inside the rectangle shows the median and whiskers above and below the box show the locations of the minimum and maximum.

(c) The heatmaps of H3K4me3 or H3K27ac signal in ChIP-seq against the number of PETs.

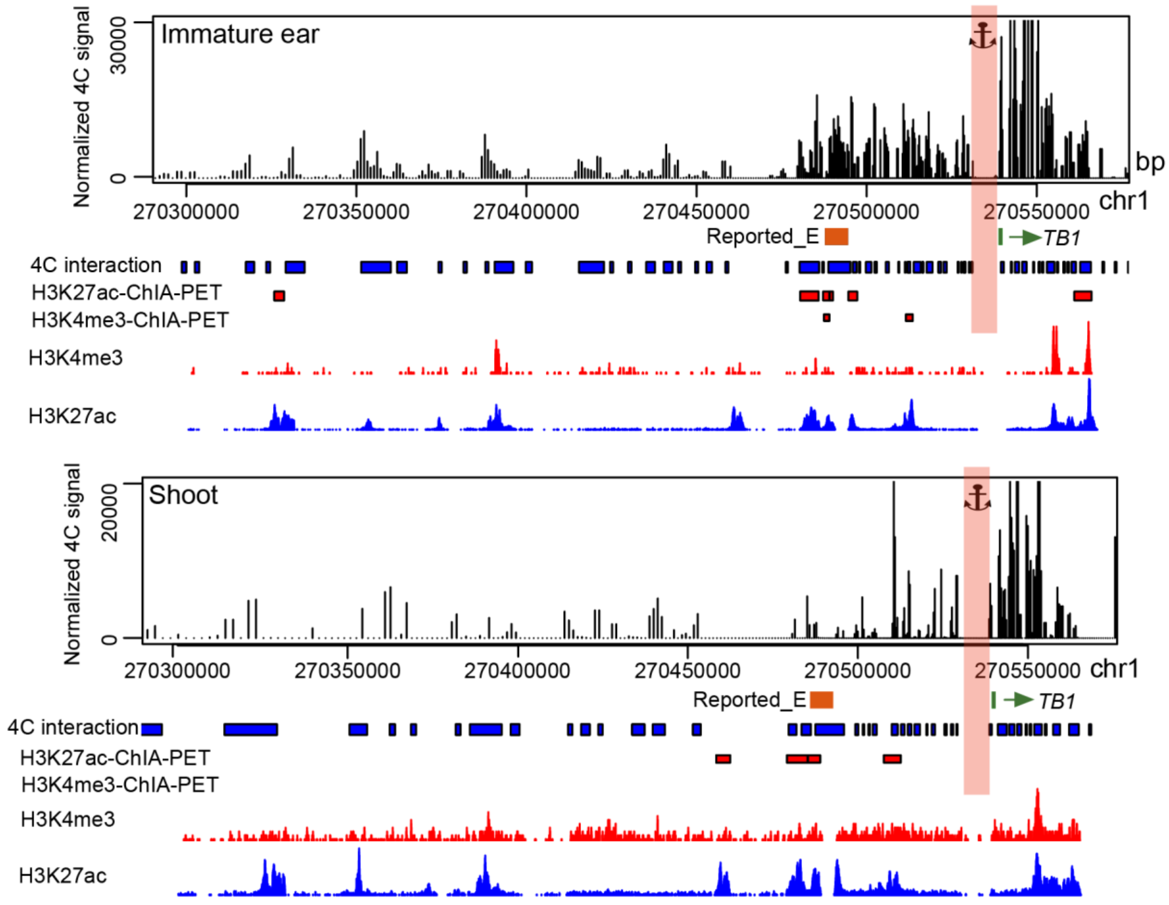


**Supplementary Figure 4. Validation of*TB1* in the ChIA-PET interaction by 4C-seq.** The green rectangle represents the corresponding gene. The orange rectange indicates the reported regions regulating the corresponding genes.The blue rectangle indicates the regions interacting with viewpoint in 4C-seq. The red rectangle indicates the regions interacting with viewpoint in ChIA-PET data. Source Data are provided as a Source Data file.

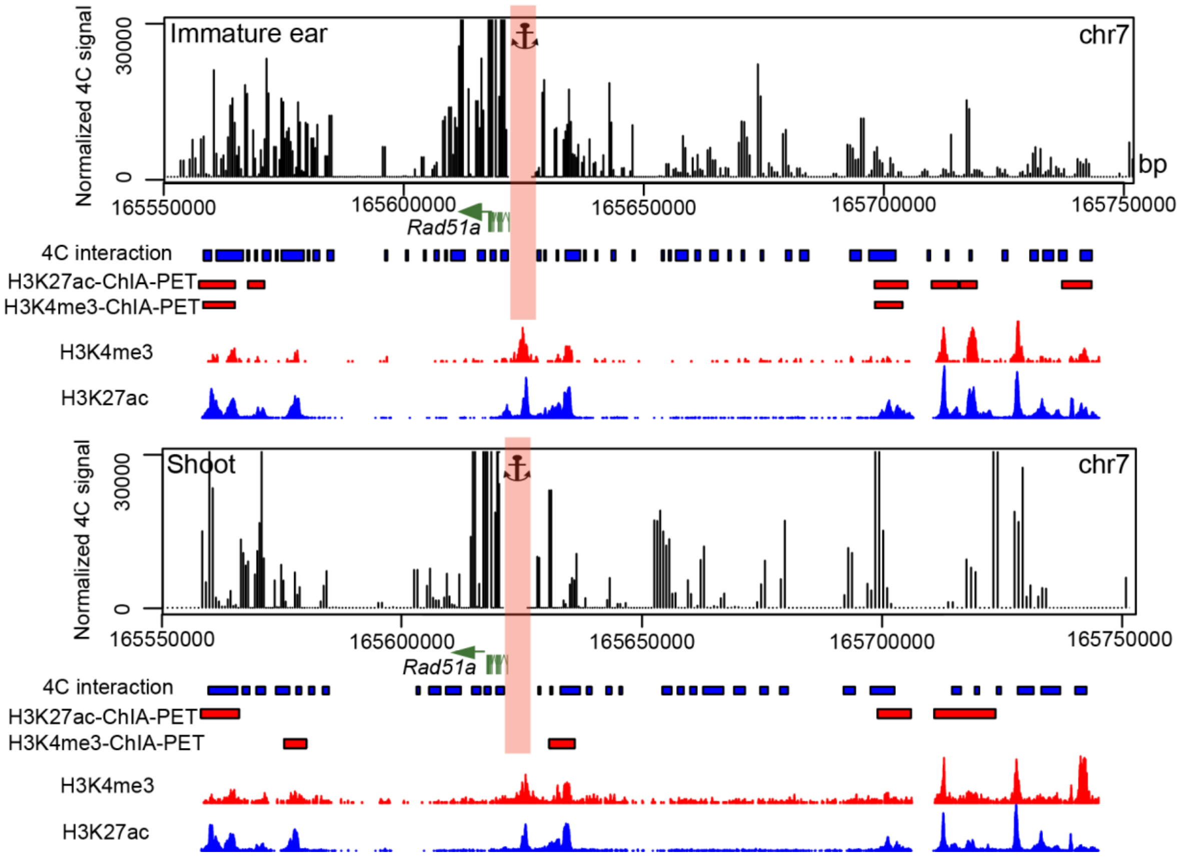


**Supplementary Figure 5. Validation of *Rad51a* in the ChIA-PET interaction by 4C-seq.**

The green rectangle represents the corresponding gene. The blue rectangle indicates the regions interacting with viewpoint in 4C-seq. The red rectangle indicates the regions interacting with viewpoint in ChIA-PET data. Source Data are provided as a Source Data file.

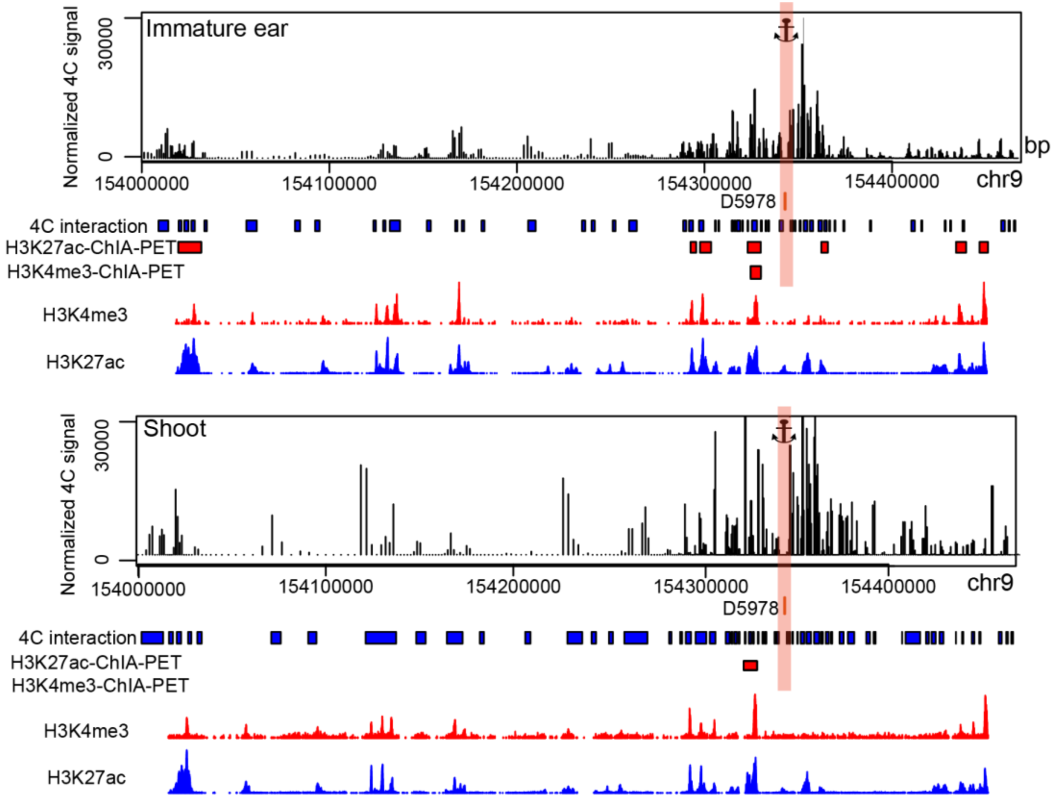


**Supplementary Figure 6. Validation of D5978 in the ChIA-PET interaction by 4C-seq.** The orange rectange representsthe corresponding D.The blue rectangle indicates the regions interacting with viewpoint in 4C-seq. The red rectangle indicates the regions interacting with viewpoint in ChIA-PET data. Source Data are provided as a Source Data file.

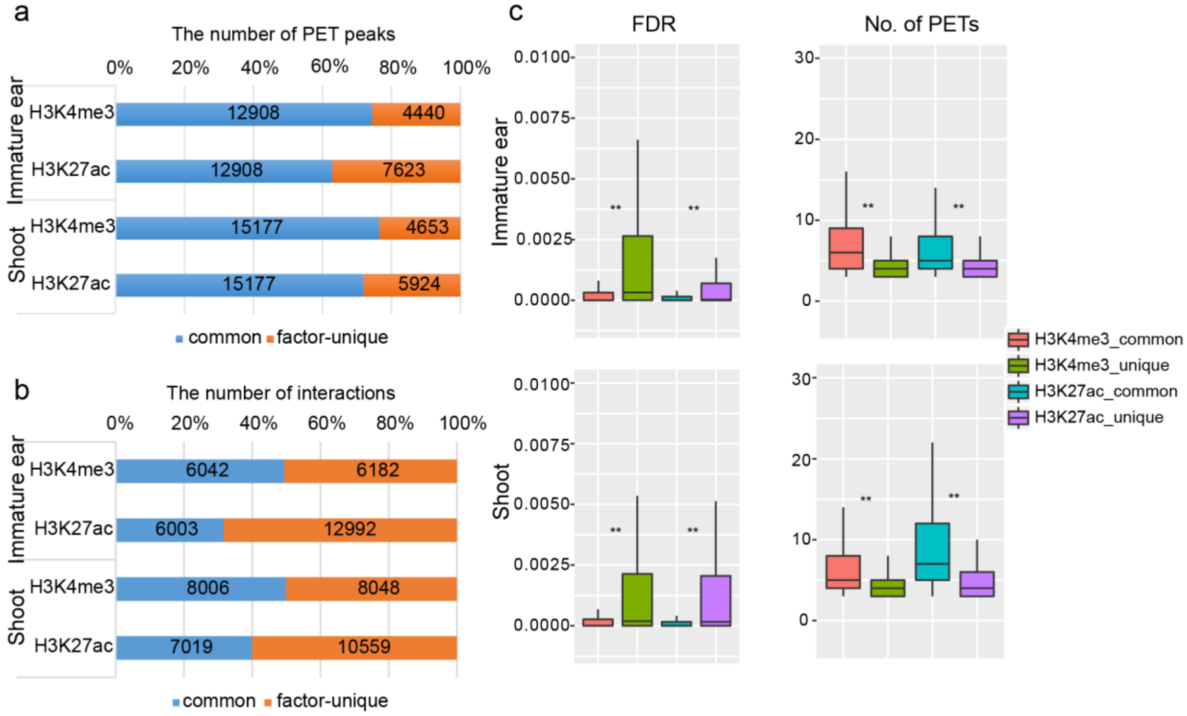


**Supplementary Figure 7. Repeatability analysis between two histone modifications-mediated ChIA-PET datasets.**

(a) The bar plot shows the number of common and factor-unique intra-chromosomal (intrachr.) PET peaks between H3K27ac- and H3K4me3-mediated ChIA-PET datasets in immature ear and shoot, respectively.

(b) The bar plot shows the number of common and factor-unique intrachr. chromatin interactions between H3K27ac- and H3K4me3-mediated ChIA-PET datasets in immature ear and shoot, respectively.

(c) Comparison of FDR and the number of PETs of common and factor-specific chromatin interactions between H3K27ac- and H3K4me3-mediated ChIA-PET datasets in immature ear and shoot, respectively. The left panel shows the comparison of FDR between common and factor-specific interactions. And the right panel shows the comparison of the number of PETs between those two types of interactions (***p* -value<0.01,**p*-value< 0.05, two-sided t-test).In the box plot, the central rectangle spans the first quartile to the third quartile (the interquartile range). A segment inside the rectangle shows the median and whiskers above and below the box show the locations of the minimum and maximum.

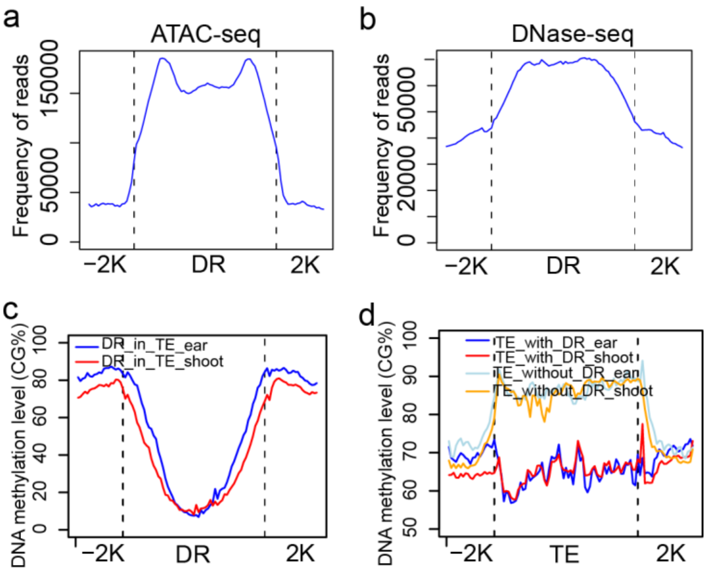


**Supplementary Figure 8**. **Characterization of distal regulatory regions (DRs) and relationship between DRs and transposable elements (TEs) in immature ear and shoot.**

(a) The profile of ATAC-seq reads around DRs in shoot.

(b) The profile of DNase-seq reads around DRs in shoot.

(c) The DNA methylation profile (CG) of DR overlapped with TE in immature ear and shoot.

(d) Comparison of DNA methylation (CG) between TE overlapped and non-overlapped with DRs in immature ear and shoot.

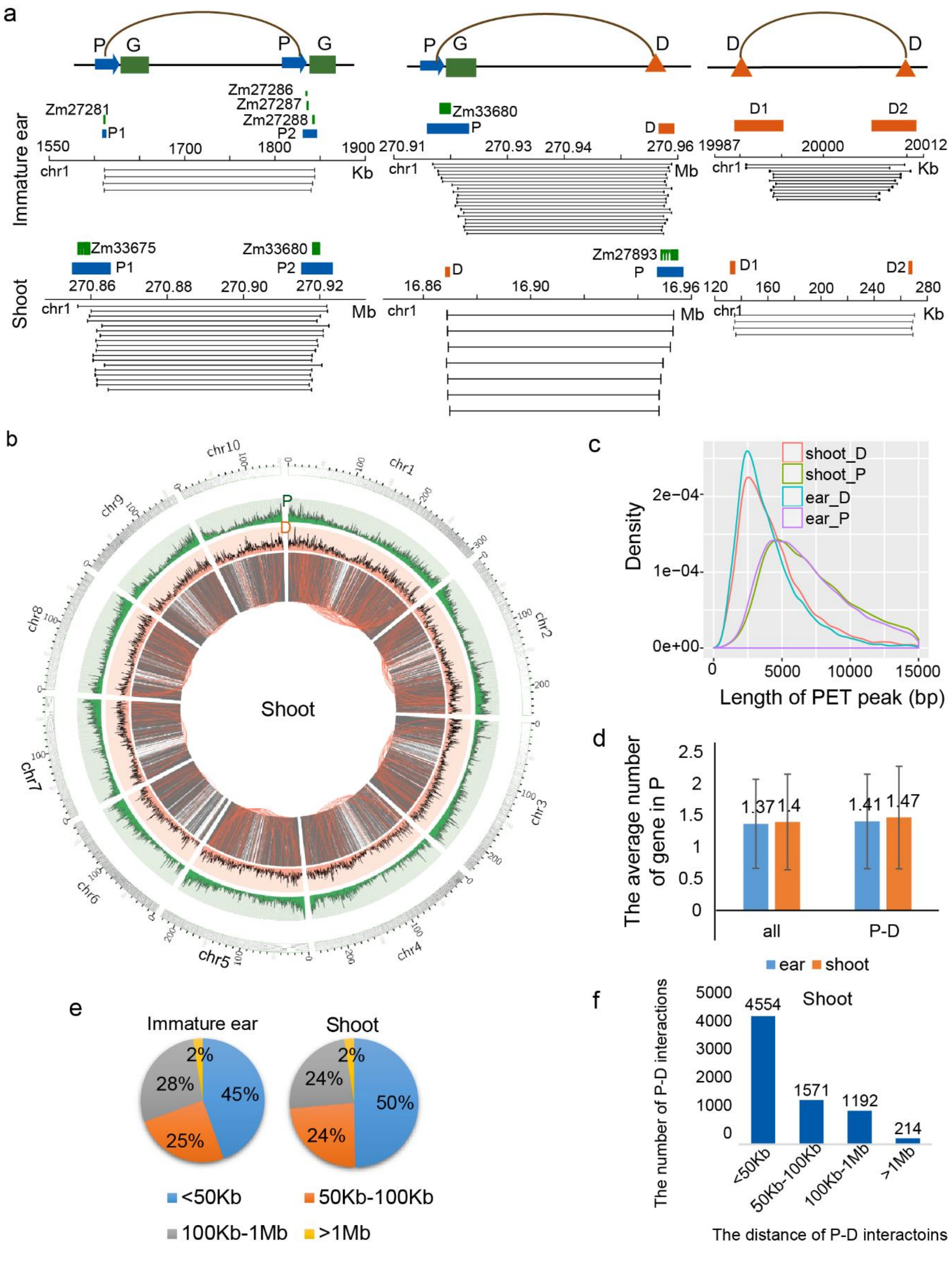


**Supplementary Figure 9**. **Annotation and characterization of chromatin interactions in immature ear and shoot.**

(a) The model of three types of chromatin interactions: proximal-proximal (P-P),

proximal-distal (P-D) and distal-distal (D-D) (the upper panel). Examples for each case are displayed in immature ear (middle panel) and shoot (bottom panel). The distances of interactions shown were not in virtual proportional. The rectangles in blue and orange represent P and D, respectively.

(b) Circos map of the whole-genome chromatin interactions from chromosome 1 to chromosome 10 in shoot. Intra-chromosomal interactions are drawn in the innermost ring, followed by the DR (dark orange) and gene density track (dark green). Chromatin interactions with distance longer than 1Mb are highlighted by orange arcs.

(c) The density for length of all distal and proximal PET peaks in immature ear and shoot.

(d) The average number of gene in P associated with all intrachr. interactions and P-D interactions in immature ear and shoot.

(e)The distribution of distance of all chromatin interactions in immature ear and shoot, respectively.

(f) Summary of the number of P-D interaction with variable distances in shoot.

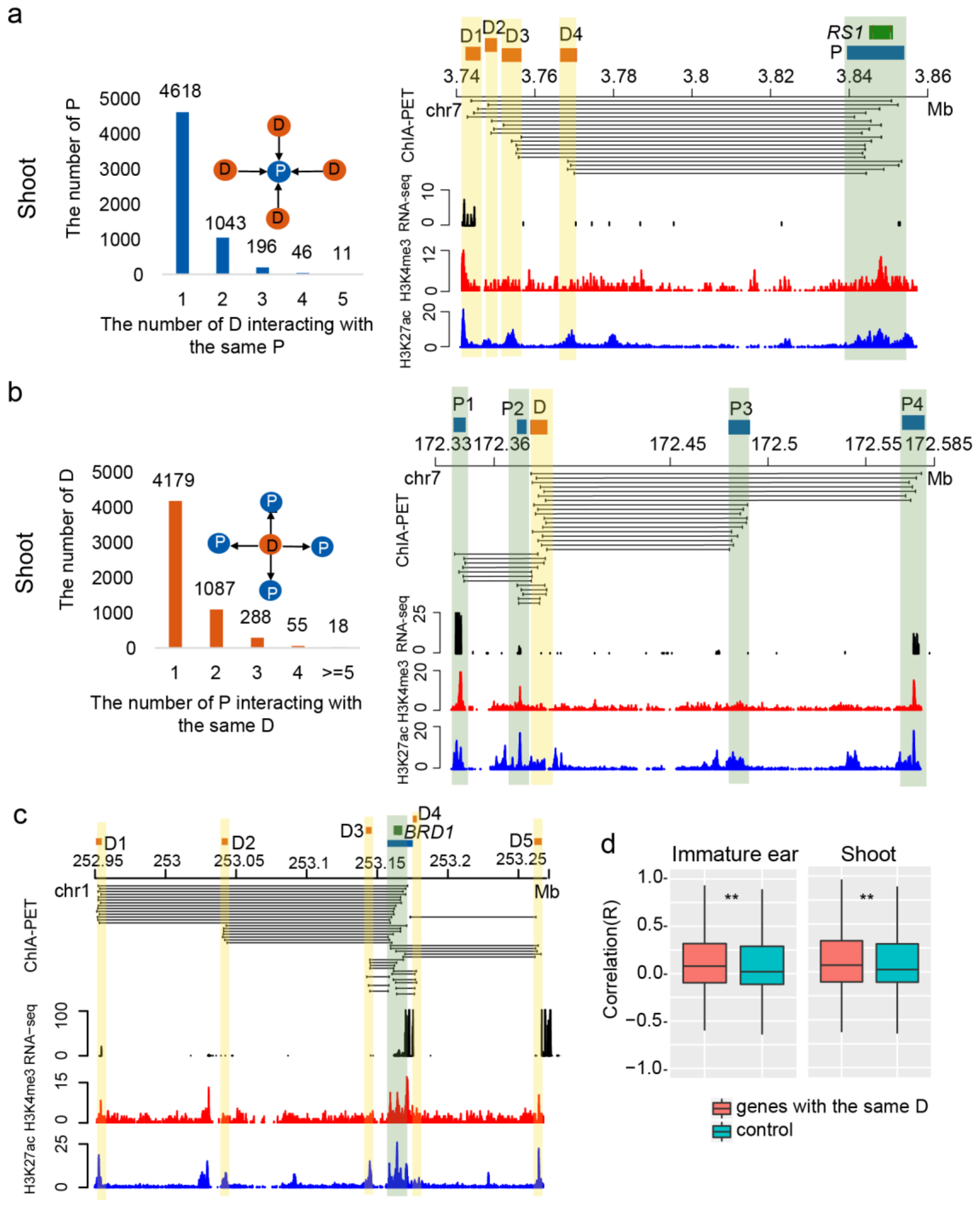

**Supplementary Figure 10. Interaction degree between proximal and distal regulatory regions in shoot.**

(a) The left panel is the summary bar chart showing the number of P(x axis) interacting with various number of D(y axis) in shoot. The corresponding right panel is an example of one P (*RS1*) interacting with four D in shoot.

(b) The left panel is the summary bar chart showing the number of D(x axis) interacting

with various number of P (y axis) in shoot. The corresponding right panel is an example of a D interacting with four different P in shoot.

(c) An example of one P (*BRD1*) interacted with five different D in shoot.

(d) The comparison of expression correlation between genes interacting with the same Dwith control in immature ear and shoot (ear: ***p* -value =1.251e-08, shoot: ***p*-value =0.001, two-sided t-test).To get gene pairs in control, we randomly shifted gene pairs interacting with the same D 1000 times, and filtered those without H3K4me3 / H3K27ac signal.

In the box plot, the central rectangle spans the first quartile to the third quartile (the interquartile range). A segment inside the rectangle shows the median and whiskers above and below the box show the locations of the minimum and maximum. We used the collected RNA-seq dataset asreported[9], consisting of 53 different seed and 25 non-seed samples in maize, then calculated the Shannon entropy of each gene. Source Data of Supplementary Fig. 10a and10b are provided as a Source Data file.

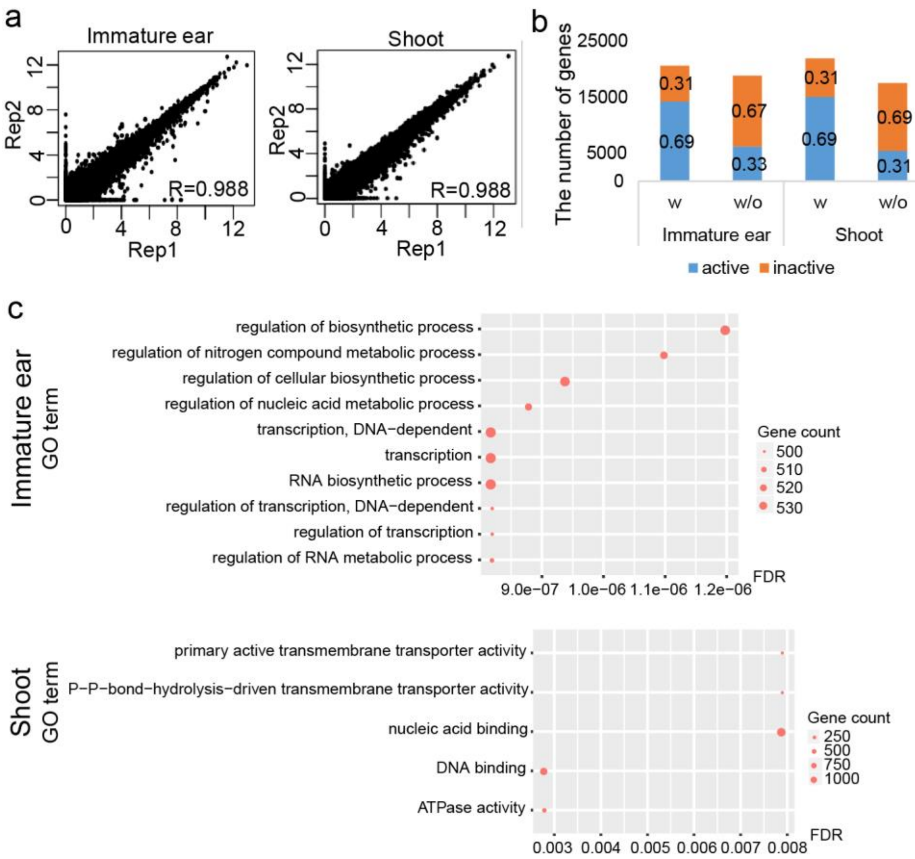


**Supplementary Figure 11.Repeatability analysis between replicates of RNA-seq datasets andcharacterization of genes interacting with D.**

(a) Correlation between biological replicates of immature ear and shoot for RNA-seq. The normalized data of $\log_2$ (FPKM +1) was used to calculate the correlation coefficient.

(b) Transcriptional status of genes with (w) or without (w/o) P-P interactions. The bar plot shows the number of corresponding genes with the proportion labeled on the bar. ‘inactive’ indicates gene with FPKM <1, ‘active’ indicates gene with FPKM >=1. ‘w’ represents genes with P-P interactions, ‘w/o’ represents genes without P-P interactions.

(c) Enrichment of top10and top5 GO terms for genes interacting with D in immature ear and shoot, respectively. Genes enriched with H3K4me3/H3K27ac were used as control.

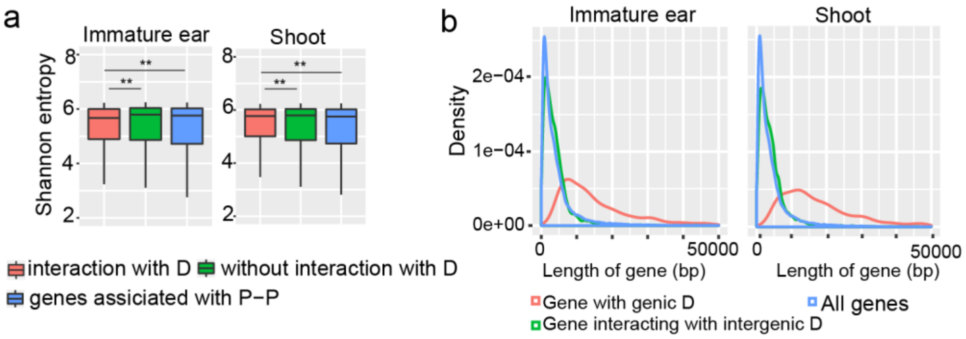


**Supplementary Figure 12.Comparison of length of genes interacting with genic or intergenic D.**

(a) Comparison of Shannon entropy of genes interacting with interactions, gene not interacting with interactions and enriched with H3K4me3 / H3K27ac, and genes with P-P (***p*-value < 0.01, two-sided t-test).In the box plot, the central rectangle spans the first quartile to the third quartile (the interquartile range). A segment inside the rectangle shows the median and whiskers above and below the box show the locations of the minimum and maximum.

(b) Comparison of length of gene with genic or intergenic D (both *p*-value < 2.2e-16, two-sided t-test).

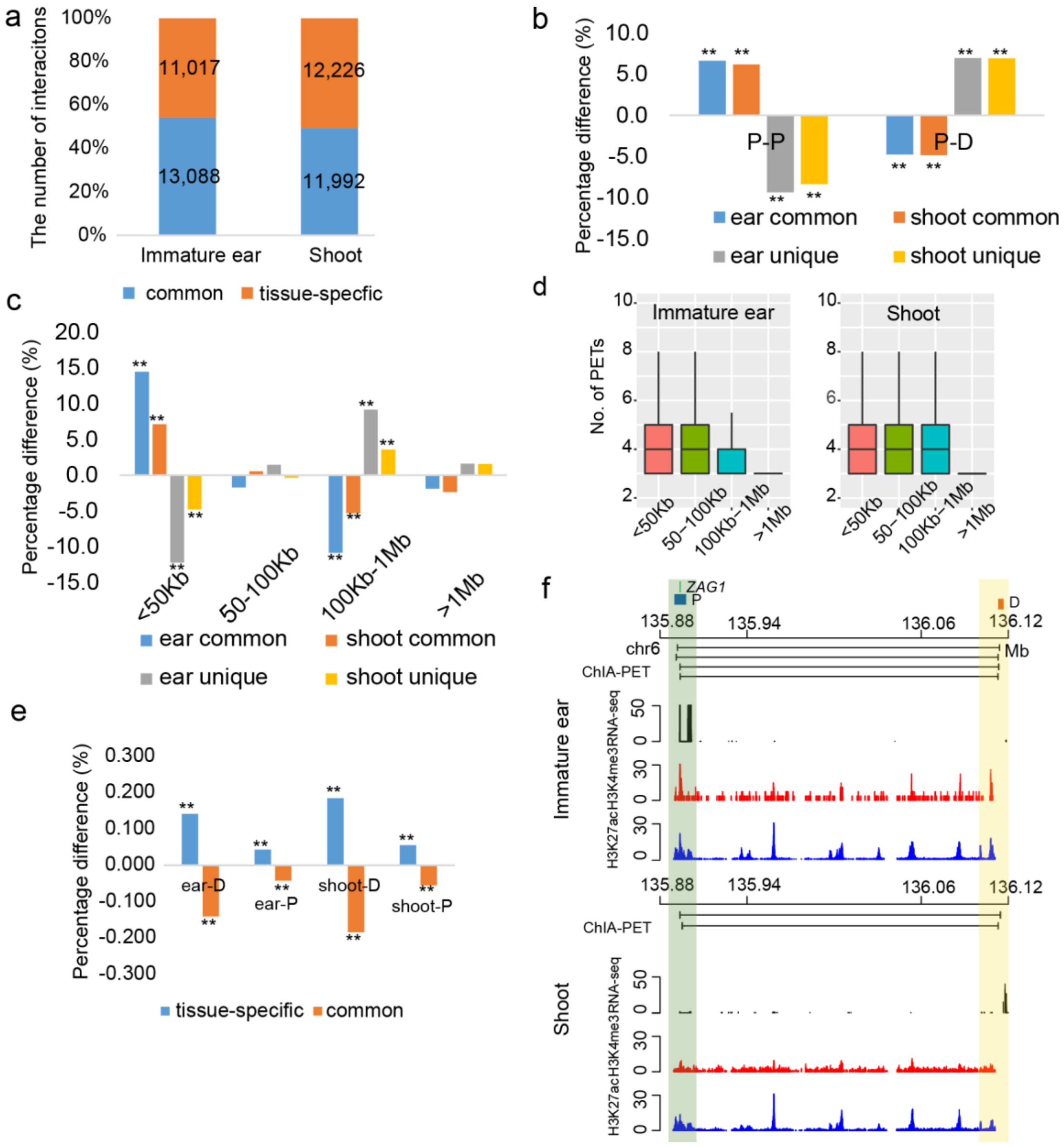


**Supplementary Figure 13. Identification and characterizationof tissue-specific chromatin interaction in immature ear and shoot.**

(a) Identification of common and tissue-specific chromatin interactions in maize.

(b) Comparison of the enrichment of P-P and P-D in common and tissue-specific interactions between immature ear and shoot (***p*-value<0.01,**p*-value< 0.05, Fisher's Exact Test).

(c) Comparison of the distance enrichment of P-D in common and tissue-specific interactions between immature ear and shoot (***p*-value<0.01,**p*-value< 0.05, Fisher's Exact Test).

(d) Comparison of the number of PETs for interactions with variant distance. In the box plot, the central rectangle spans the first quartile to the third quartile (the interquartile range). A segment inside the rectangle shows the median and whiskers above and below the box show the locations of the minimum and maximum.

(e)Comparison of the enrichment of tissue-specific D in tissue-specific P-D and all interactions between immature ear and shoot (**$p$-value$<0.01$, *$p$-value$< 0.05$, Fisher's Exact Test).

(f) An example of immature ear-specific chromatin interaction. *ZAG1* gene (highlighted in green) interacted with distantly located D (highlighted in yellow) in immature ear. In contrast, the *ZAG1* gene in shoot is not expressed and has no interactions with that D.

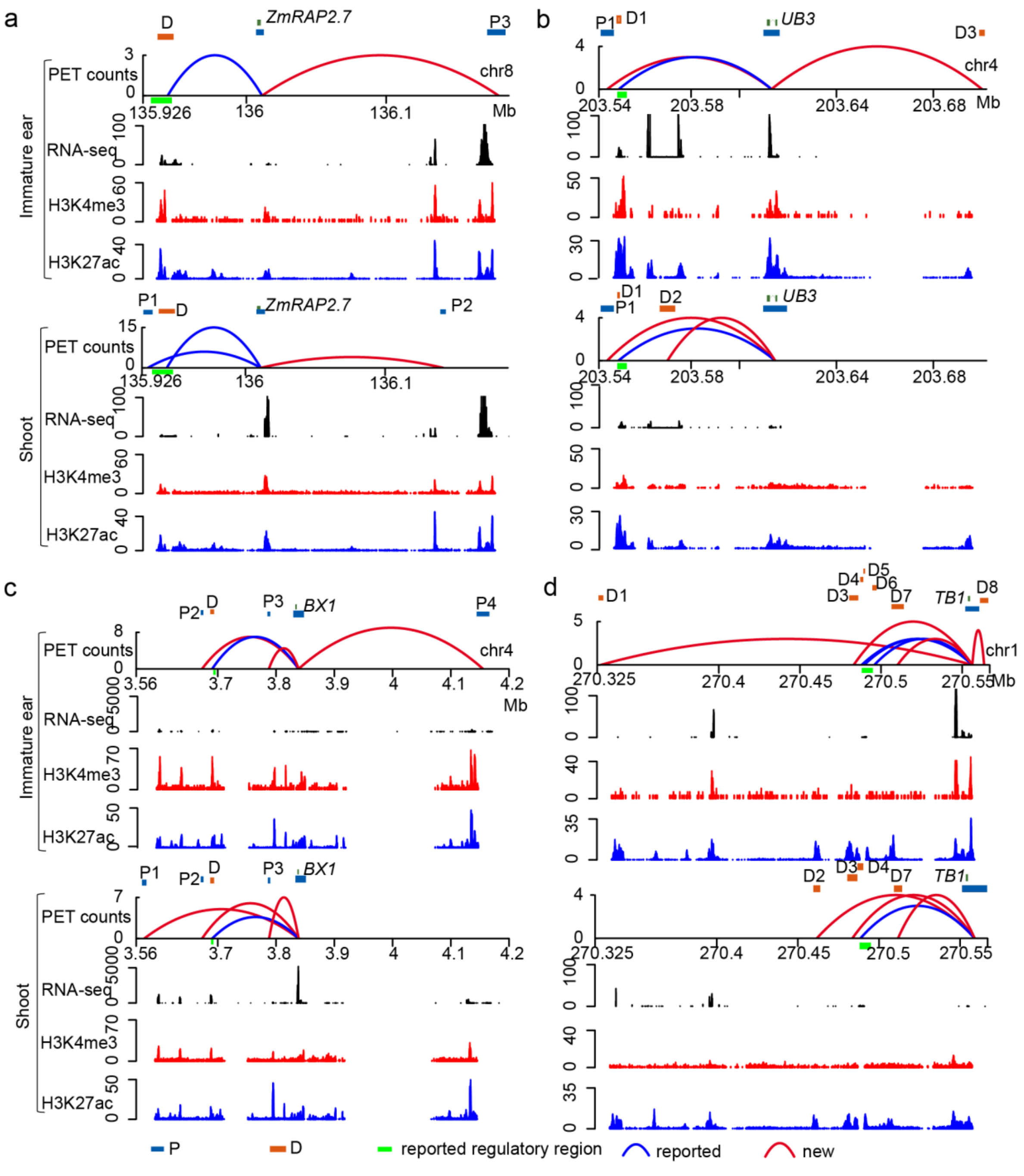


**Supplementary Figure 14. Chromatin interactions identified for genes with reported long-range regulation regions related with agronomic traits**

Long-range chromatin interaction for *ZmRAP2.7* (a), *UB3* (b), *BX1*(c), *TB1* (d) in immature ear and shoot.

(a) Long-range chromatin interaction for *ZmRAP2.7* in immature ear and shoot.

D1: overlapped with Vgt1; P1: Zm00001d010985\Zm00001d010986; P2: Zm00001d010995\Zm00001d010997; P3: Zm00001d010993\Zm00001d010994;

(b) Long-range chromatin interaction for *UB3* in immature ear and shoot.

D1: chr4:203549552-203550620 (overlapped with KRN4); D2: chr4:203567114-203573471; D3: chr4:203698942-203701233; P1: Zm00001d052889;

(c) Long-range chromatin interaction for *BX1* in immature ear and shoot.

D1: chr4:3687181-3693685; P1: Zm00001d048702; P2: Zm00001d048705; P3: Zm00001d048707; P4: Zm00001d048717\Zm00001d048716\Zm00001d048718;

(d) Long-range chromatin interaction for *TB1*in immature ear and shoot.

D1: chr1:270325870-270328735; D2: chr1:270459596-270463715; D3: chr1: 270480473-270485884; D4: chr1:270487195-270489024; D5: chr1:270489171-270490112; D6: chr1:270494627-270497240; D7: chr1:270506447-270514057; D8: chr1:270561051-270566120;

The green rectangle indicated the reported regulatory region of the corresponding gene. The rectangles in blue and orange represent P and D, respectively.

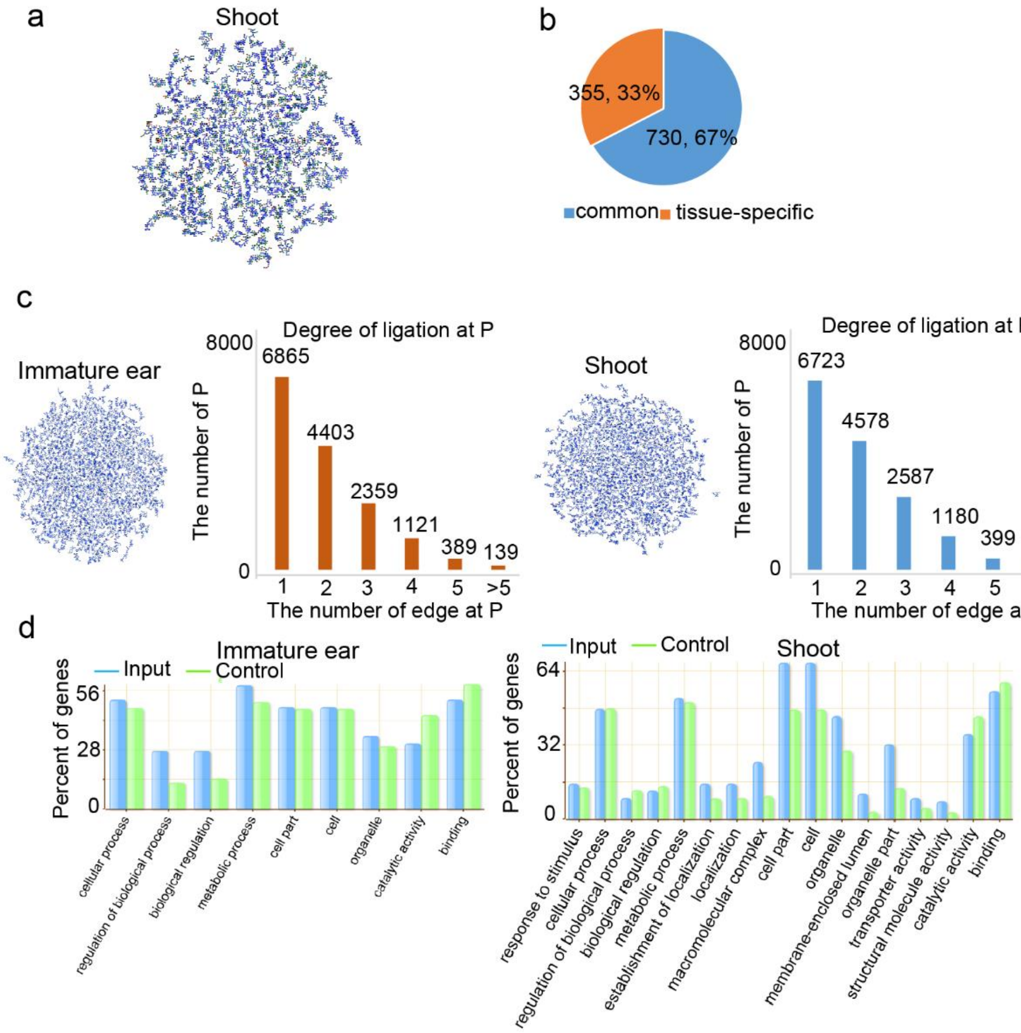


**Supplementary Figure 15. Characterization of proximal-proximal interactions in network.**

(a) All the chromatin interaction networks identified in shoot. Here showed alone the networks with more than 30 nodes

(b) Proportion of tissue-specific (shoot, for illustration) communities and the communities common (>65% overlap) to immature ear and shoot. Here communities only contain proximal-proximal interaction.

(c) The network of gene and the ligation degree of gene in the network of chromatin interaction in immature ear and shoot, respectively.

(d) AgriGo analysis for genes with top50 highest ligation degree in network in immature ear and shoot, respectively. Genes with lowest ligation degree in network were as control.

**Supplementary Table 1. Statistical summary of ChIA-PET libraries in immature ear and shoot.**

| Tissue | Factor | Replicate | Raw read | Reads with bridge linker | Uniquely mapped reads | Uniquely mapped PETs |
|---|---|---|---|---|---|---|
| Immature ear | H3K4me3 | Rep1 | 945,831,154×2 | 658,623,638×2 | 576,592,875 | 118,396,408 |
| | | Rep2 | 448,555,837×2 | 310,087,684×2 | 267,037,099 | 54,230,937 |
| | H3K27ac | Rep1 | 760,124,767×2 | 543,573,117×2 | 345,556,618 | 51,671,423 |
| | | Rep2 | 318,858,124×2 | 234,376,576×2 | 219,222,488 | 50,380,701 |
| Shoot | H3K4me3 | Rep1 | 837,949,721×2 | 497,583,676×2 | 471,481,036 | 91,526,325 |
| | | Rep2 | 373,916,852×2 | 265,597,506×2 | 236,963,342 | 53,089,394 |
| | H3K27ac | Rep1 | 763,064,872×2 | 492,473,829×2 | 488,443,148 | 117,220,237 |
| | | Rep2 | 723,908,023×2 | 448,962,331×2 | 331,521,680 | 54,415,906 |

**Supplementary Table 2. Statistical summary of ChIP-seq libraries in immature ear and shoot.**

| Tissue | Factor | Replicate | Raw read | Uniquely mapped reads | No. of peaks |
|---|---|---|---|---|---|
| Immature ear | H3K4me3 | Rep1 | 41,307,461×2 | 59,593,796 | 26,312 |
| | | Rep2 | 41,967,630×2 | 50,938,786 | |
| | H3K27ac | Rep1 | 50,097,917×2 | 82,544,513 | 56,766 |
| | | Rep2 | 49,620,675×2 | 82,729,079 | |
| Shoot | H3K4me3 | Rep1 | 44,404,978×2 | 52,551,650 | 27,164 |
| | | Rep2 | 50,302,675×2 | 65,332,774 | |
| | H3K27ac | Rep1 | 44,450,065×2 | 66,012,019 | 56,707 |
| | | Rep2 | 49,989,168×2 | 71,870,900 | |

**Supplementary Table 3. Percentage of intra-chromosomal interaction from each ChIA-PET libraryin immature ear and shoot.**

| Tissue | Factor | Intrachr. interaction | Interchr. interaction | Intrachr. % |
|---|---|---|---|---|
| Immature ear | H3K4me3 | 12,120 | 1,879 | 87 |
| | H3K27ac | 18,864 | 2,115 | 90 |
| shoot | H3K4me3 | 15,826 | 1,995 | 89 |
| | H3K27ac | 17,211 | 2,172 | 89 |

Note: Intrachr. Interaction: intra-chromosomal interaction; Interchr. Interaction: inter-chromosomal interaction.

**Supplementary Table 4. Summary the primer sequences used in ChIP-seq, ChIA-PET and 4C-seq experiments**.

| | Loci | (5'-3') | Sequence of primer |
|---|---|---|---|
| ChIP-seq and ChIA-PET | *actin*_exon2[1] | Forward | GATGATGCGCCAAGAGCTG |
| | | Reverse | GCCTCATCACCTACGTAGGCAT |
| | *GAPDH*_upstream_1kb | Forward | AGCTATCAGGTCTGTGCAAGC |
| | | Reverse | AGCTTTTCCATCCCTTTACCA |
| | H3K27ac_1[2] | Forward | GTGCCTATGTCTCCTCTTCTTG |
| | | Reverse | TCCAAGTGACCGTGAGAATTT |
| | H3K27ac_2[2] | Forward | CGTTTCCCGATTCGGTAGTT |
| | | Reverse | GATCTGCTCCATGTCTACCAAG |
| | *Copia*[1] | Forward | CGATGTGAAGACAGCATTCCT |
| | | Reverse | CTCAAGTGACATCCCATGTGT |
| 4C-seq | Viewpoints | (5'-3') | Sequence of primer |
| | *Rad51a* | Forward | GTTTATATAATGCCTCGATC |
| | | Reverse | GGCACTTACAACTATATCCA |
| | *UB3* | Forward | CGGCGTCCTCGAAGTAGATC |
| | | Reverse | ATGCTTGGTTTGAATGGTGC |
| | *TB1* | Forward | CGAAGTCTCTGAGTATGATC |
| | | Reverse | GGGTTCAAAGCACCAACAGT |
| | D5978 | Forward | CTTTTCTCCGGCCGGTGATC |
| | | Reverse | AGTCACAGACAAATTGGAGA |

**Supplementary Table 5. Summary of the numbers of gene pairs or genes in Figure 5.**

| | | Immature ear | Shoot |
|---|---|---|---|
| Fig 5a. | with interaction | 21,989 | 23,246 |
| | without interaction | 17,367 | 16,125 |
| Fig 5c. | P-P | 6,333 | 6,182 |
| | control | 16,179 | 16,803 |
| Fig 5d. | Gene not interacting with D | 17,894 | 17,595 |
| | Gene interacting with intergenic D | 8,283 | 7,287 |
| | Gene interacting with genic D | 2,598 | 3,451 |
| | Gene with D in its gene body | 1,933 | 2,724 |
| Fig 5e. | No. of intergenic D | Immature ear | Shoot |
| | 1 | 2,209 | 2,033 |
| | 2 | 507 | 314 |
| | >=3 | 198 | 85 |

**Supplementary Table 6. Summary of published data used in this study.**

| Tissue | Data type | SRA accession |
|---|---|---|
| Shoot | RNA-seq | SRR1765337[https://sra-download.ncbi.nlm.nih.gov/traces/sra24/SRR/001723/SRR1765337] |
| | | SRR1765338[https://sra-download.ncbi.nlm.nih.gov/traces/sra24/SRR/001723/SRR1765338] |
| Shoot | MethyC-seq | SRR448335[https://sra-download.ncbi.nlm.nih.gov/traces/sra3/SRR/000437/SRR448335] |
| | | SRR448339[https://sra-download.ncbi.nlm.nih.gov/traces/sra3/SRR/000437/SRR448339 ] |
| Immatureear | MethyC-seq | SRR1583945[https://sra-download.ncbi.nlm.nih.gov/traces/sra21/SRR/001546/SRR1583945] |
| | | SRR1583946[https://sra-download.ncbi.nlm.nih.gov/traces/sra21/SRR/001546/SRR1583946] |
| | | SRR1583947[https://sra-download.ncbi.nlm.nih.gov/traces/sra21/SRR/001546/SRR1583947] |
| Shoot | ATAC-seq | SRR5748809[https://sra-download.ncbi.nlm.nih.gov/traces/sra49/SRR/005614/SRR5748809] |
| | | SRR5748810[https://sra-download.ncbi.nlm.nih.gov/traces/sra49/SRR/005614/SRR5748810] |
| Shoot | DNase-seq | SRR5436230[https://sra-download.ncbi.nlm.nih.gov/traces/sra47/SRR/005308/SRR5436230] |

## Supplementary References


1. Haring, M. et al. Chromatin immunoprecipitation: optimization, quantitative analysis and data normalization. *Plant Methods* **3**, 11 (2007).
2. Zhang, W., Garcia, N., Feng, Y., Zhao, H. & Messing, J. Genome-wide histone acetylation correlates with active transcription in maize. *Genomics* **106**, 214-20 (2015).
3. Fullwood, M.J. et al. An oestrogen-receptor-alpha-bound human chromatin interactome. *Nature* **462**, 58-64 (2009).
4. Tang, Z. et al. CTCF-Mediated Human 3D Genome Architecture Reveals Chromatin Topology for Transcription. *Cell* **163**, 1611-27 (2015).
5. Hovel, I., Louwers, M. & Stam, M. 3C Technologies in plants. *Methods* **58**, 204-11 (2012).
6. Splinter, E., de Wit, E., van de Werken, H.J., Klous, P. & de Laat, W. Determining long-range chromatin interactions for selected genomic sites using 4C-seq technology: from fixation to computation. *Methods* **58**, 221-30 (2012).
7. Mumbach, M.R. et al. HiChIP: efficient and sensitive analysis of protein-directed genome architecture. *Nat Methods* **13**, 919-922 (2016).
8. Rao, S.S. et al. A 3D map of the human genome at kilobase resolution reveals principles of chromatin looping. *Cell* **159**, 1665-80 (2014).
9. Chen, J. et al. Dynamic transcriptome landscape of maize embryo and endosperm development. *Plant Physiol* **166**, 252-64 (2014).
