# Supplementary material for: Long-range interactions between proximal and distal regulatory regions in maize
Source: Nat Commun. 2019 Jun 14;10:2633. doi: 10.1038/s41467-019-10603-4 (PMC6572780; doi:10.1038/s41467-019-10603-4)
Supplement: Supplementary file 3 — Reporting Summary [file 41467_2019_10603_MOESM3_ESM.pdf]

nature research

Corresponding author(s): En Li

Last updated by author(s): May 6, 2019

Reporting Summary

Nature Research wishes to improve the reproducibility of the work that we publish. This form provides structure for consistency and transparency in reporting. For further information on Nature Research policies, see Authors & Referees and the Editorial Policy Checklist.

Statistics

For all statistical analyses, confirm that the following items are present in the figure legend, table legend, main text, or Methods section.

n/a	Confirmed	
☐	☒	The exact sample size (*n*) for each experimental group/condition, given as a discrete number and unit of measurement
☐	☒	A statement on whether measurements were taken from distinct samples or whether the same sample was measured repeatedly
☐	☒	The statistical test(s) used AND whether they are one- or two-sided<br>*Only common tests should be described solely by name; describe more complex techniques in the Methods section.*
☐	☒	A description of all covariates tested
☐	☒	A description of any assumptions or corrections, such as tests of normality and adjustment for multiple comparisons
☐	☒	A full description of the statistical parameters including central tendency (e.g. means) or other basic estimates (e.g. regression coefficient) AND variation (e.g. standard deviation) or associated estimates of uncertainty (e.g. confidence intervals)
☐	☒	For null hypothesis testing, the test statistic (e.g. *F*, *t*, *r*) with confidence intervals, effect sizes, degrees of freedom and *P* value noted<br>*Give P values as exact values whenever suitable.*
☒	☐	For Bayesian analysis, information on the choice of priors and Markov chain Monte Carlo settings
☒	☐	For hierarchical and complex designs, identification of the appropriate level for tests and full reporting of outcomes
☐	☒	Estimates of effect sizes (e.g. Cohen's *d*, Pearson's *r*), indicating how they were calculated

Our web collection on statistics for biologists contains articles on many of the points above.

Software and code

Policy information about availability of computer code

Data collection	NA
Data analysis	All softwares used in our data analysis:bowtie-0.12.9; bowtie2-2.1.0; MACS-1.4.2; tophat-2.0.9, cufflinks-2.2.1; R-3.2.3, http://www.r-project.org/; Circos, http://circos.ca/; Agrigo, http://systemsbiology.cau.edu.cn/agriGOv2/index.php; Cytoscape, http://www.cytoscape.org/.<br>Source code is publicly available at available in the GitHub repository (https://github.com/liencau/ChIA-PET-analysis).

For manuscripts utilizing custom algorithms or software that are central to the research but not yet described in published literature, software must be made available to editors/reviewers. We strongly encourage code deposition in a community repository (e.g. GitHub). See the Nature Research guidelines for submitting code & software for further information.

Data

Policy information about availability of data

All manuscripts must include a data availability statement. This statement should provide the following information, where applicable:

- Accession codes, unique identifiers, or web links for publicly available datasets
- A list of figures that have associated raw data
- A description of any restrictions on data availability

All ChIA-PET, ChIP-seq, 4C and RNA-seq Data are accessible at SRP162618, SRP158640, SRP154106, SRP158820. RNA-seq, MethyC-seq, ATAC-seq and DNase-seq of shoot and MethyC-seq of immature ear were published previously, and have been deposited in the NCBI Sequence Read Archive under accession number SRP052520 , SRP011991, PRJNA391551, PRJNA382414, SRP047420, respectively.

Field-specific reporting

Please select the one below that is the best fit for your research. If you are not sure, read the appropriate sections before making your selection.

☒ Life sciences ☐ Behavioural & social sciences ☐ Ecological, evolutionary & environmental sciences

For a reference copy of the document with all sections, see nature.com/documents/nr-reporting-summary-flat.pdf

Life sciences study design

All studies must disclose on these points even when the disclosure is negative.

Sample size	All ChIA-PET, ChIP-seq, 4C and RNA-seq Data are accessible at SRP162618, SRP158640, SRP154106, SRP158820. RNA-seq, MethyC-seq, ATAC-seq and DNase-seq of shoot and MethyC-seq of immature ear were published previously, and have been deposited in the NCBI Sequence Read Archive under accession number SRP052520 , SRP011991, PRJNA391551, PRJNA382414, SRP047420, respectively.
Data exclusions	NA
Replication	There was high reproducibility between replicates as indicated by the repeatability of biological replicates at different levels, including library reads and peaks of chromatin interaction PETs.
Randomization	NA
Blinding	NA

Reporting for specific materials, systems and methods

We require information from authors about some types of materials, experimental systems and methods used in many studies. Here, indicate whether each material, system or method listed is relevant to your study. If you are not sure if a list item applies to your research, read the appropriate section before selecting a response.

Materials & experimental systems

n/a	Involved in the study	
☐	☒	Antibodies
☒	☐	Eukaryotic cell lines
☒	☐	Palaeontology
☒	☐	Animals and other organisms
☒	☐	Human research participants
☒	☐	Clinical data

Methods

n/a	Involved in the study	
☐	☒	ChIP-seq
☒	☐	Flow cytometry
☒	☐	MRI-based neuroimaging

Antibodies

Antibodies used	H3K4me3 (1 μg/μl, Abcam, cat.No.ab8580), H3K27ac (1 μg/μl, Abcam,cat.No.ab4729)
Validation	The specificity of antibodies was validated by dot blot. For H3K27ac antibody, peptides array including 13 different acetylated peptides was used: H4K5ac,H4K18ac,H4K12ac,H4K16ac,H4K5/8/12ac,H4K91ac,H3K9ac,H3K14ac,H3K9/14ac,H3K18ac,H3K23ac,H3K27ac and H3K56ac. For H3K4me3 antibody, peptides array including 30 different methylated peptides was used: H4R3me1, H4R3me2s, H4R3me2a, H4K20me1, H4K20me2, H4K20me3, H3R2me1, H3R2me2s, H3R2me2a, H3K4me1, H3K4me2, H3K4me3, H3R8me2a, H3K9me1, H3K9me2, H3K9me3,H3R17me2s, H3R17me2a, H3R26me1, H3R26me2a, H3K27me1, H3K27me2, H3K27me3, H3K36me1, H3K36me2, H3K36me3,H3K56me1, H3K79me1, H3K79me2, H3K79me3. Antibodies passed by dot blot validation were futher tested by western blot. For western blot, nuclear extracts of maize endosperm and shoot, and mouse embryonic stem cells and fibroblast cells, were used.

ChIP-seq

Data deposition

☒ Confirm that both raw and final processed data have been deposited in a public database such as GEO.

☒ Confirm that you have deposited or provided access to graph files (e.g. BED files) for the called peaks.

Data access links<br>*May remain private before publication.*	The raw data of ChIP-seq data are accessible at SRP158640. We provided graph files for the called peaks in Supplementary Data 5-8 in this manuscript.

Files in database submission	EM-2_R1.fq.gz,EM-2_R2.fq.gz;EM-3_R1.fq.gz,EM-3_R2.fq.gz;EK-1_R1.fq.gz,EK-1_R2.fq.gz;EK-2_R1.fq.gz,EK-2_R2.fq.gz;SM-1_R1.fq.gz,SM-1_R2.fq.gz;SM-2_R1.fq.gz,SM-2_R2.fq.gz;SK-1_R1.fq.gz,SK-1_R2.fq.gz;SK-2_R1.fq.gz,SK-2_R2.fq.gz;<br>Supplementary Data 5. H3K27ac peaks from ChIP-seq library in immature ear.<br>Supplementary Data 6. H3K4me3 peaks from ChIP-seq library in immature ear.<br>Supplementary Data 7. H3K27ac peaks from ChIP-seq library in shoot.<br>Supplementary Data 8. H3K4me3 peaks from ChIP-seq library in shoot.
Genome browser session (e.g. UCSC)	NA

Methodology

Replicates	Each sample with two biological replicates,with high overlap for peaks between replicates in each sample.
Sequencing depth	The total number of reads: about 85,000,000*2~95,000,000 *2 for each sample;uniquely mapped reads: about 110,000,000~160,000,000 uniquely mapped reads for each sample; length of reads: 150bp; paired-end. The detailed information are accessible in Supplementary Table 2(Statistical summary of ChIP-seq libraries in immature ear and shoot) in this manuscript.
Antibodies	H3K4me3 (1 μg/μl, Abcam, cat.No.ab8580), H3K27ac (1 μg/μl, Abcam,cat.No.ab4729)
Peak calling parameters	read mapping : bowtie2 -t -q -N 0 -L 25 -X 2000 -p 5 -x /NAS1/lien/data/maize/B73_AGPV4/Zea_mays.AGPv4.dna.toplevel -1 ./EK-1/EK-1_R1.fq.gz -2 ./EK-1/EK-1_R2.fq.gz |samtools view -bS -q 20 - > *.bam<br>peak calling: macs14 -t *.bam -f BAM -g 2.06e+9 -n Ear_H3K4me3_map2AGPv4_peak_p5 --pvalue 1e-05 --nomodel --shiftsize 73
Data quality	Only reads with mapping quality >=20 were used for calling peaks. We used a strict criterion(p-value <=1 × 10–5) for calling peaks. The distributions of each histone mark around genes were constant with previous reports.26,029(99%) of H3K4me3 peaks, 39,664(70%) of H3K27ac peaks in immature ear, 25,448(94%) of H3K4me3 peaks, 39,071(69%) of H3K27ac peaks in shoot were above 5-fold enrichment.
Software	MACS-1.4.2
