# Supplementary material for: Long-range interactions between proximal and distal regulatory regions in maize
Source: Nat Commun. 2019 Jun 14;10:2633. doi: 10.1038/s41467-019-10603-4 (PMC6572780; doi:10.1038/s41467-019-10603-4)
Supplement: Supplementary file 4 — Description of Additional Supplementary Files [file 41467_2019_10603_MOESM4_ESM.docx]

**Description of Additional Supplementary Files**

File Name: Supplementary Data 1

Description: High-confidence chromatin interactions from H3K27ac-ChIA-PET in immature ear.

File Name: Supplementary Data 2

Description: High-confidence chromatin interactions from H3K4me3-ChIA-PET in immature ear.

File Name: Supplementary Data 3

Description: High-confidence chromatin interactions from H3K27ac-ChIA-PET in shoot.

File Name: Supplementary Data 4

Description: High-confidence chromatin interactions from H3K4me3-ChIA-PET in shoot.

File Name: Supplementary Data 5

Description: H3K27ac peaks from ChIP-seq library in immature ear.

File Name: Supplementary Data 6

Description: H3K4me3 peaks from ChIP-seq library in immature ear.

File Name: Supplementary Data 7

Description: H3K27ac peaks from ChIP-seq library in shoot.

File Name: Supplementary Data 8

Description: H3K4me3 peaks from ChIP-seq library in shoot.

File Name: Supplementary Data 9

Description: Candidate distal regulatory regions in immature ear.

File Name: Supplementary Data 10

Description: Candidate distal regulatory regions in shoot.

File Name: Supplementary Data 11

Description: Candidate distal regulatory regions in TE regions in immature ear.

File Name: Supplementary Data 12

Description: Candidate distal regulatory regions in TE regions in shoot.

File Name: Supplementary Data 13

Description: Tissue specific P-D interactions in immature ear.

File Name: Supplementary Data 14

Description: Tissue specific P-D interactions in shoot.

File Name: Supplementary Data 15

Description: Chromatin interaction network components in immature ear.

File Name: Supplementary Data 16

Description: Chromatin interaction network components in shoot.

File Name: Supplementary Data 17

Description: Enrichment of GO term in top50 ChIN components.
