# Supplementary material for: Long-range interactions between proximal and distal regulatory regions in maize
Source: Nat Commun. 2019 Jun 14;10:2633. doi: 10.1038/s41467-019-10603-4 (PMC6572780; doi:10.1038/s41467-019-10603-4)
Supplement: Supplementary file 2 — Peer Review [file 41467_2019_10603_MOESM2_ESM.pdf]

Reviewers' comments:

Reviewer #1 (Remarks to the Author):

Li et al describe a ChIA-PET dataset describing interactions between regions containing H3K4me3 or H3K27ac in maize. The primary goal of this work, documenting interaction among promoters or promoters and distal regulatory regions, is very important and will likely yield major insights into important variation for influencing agronomic traits. However, I have some concerns about the interpretations of this dataset.

One major concern is the interpretation of the meaning of the presence of the two chromatin marks used in the study. The authors claim that H3K4me3 and H3K27ac are "well known histone marks of promoter and enhancer, respectively". I am not convinced that this is true and the cited works are from mammalian systems. There are numerous differences in chromatin between plants and animals and we can not simply assume that chromatin marks that specify regions in mammals will have the same meaning in plants. Work in maize and Arabidopsis suggests that both of these marks are actually enriched at both proximal and distal regulatory regions in plants. Neither of them are specific for regions. Indeed – the authors themselves report that 70% of intrachromosomal marks are in common for the two modifications. I would encourage the authors to reduce the use of promoter and enhancer terms in this work. Instead, they can simply focus on the interactions they find that are between regions proximal (such as <2kb) to TSSs and distal region. Alternatively they could simply refer to their interacting regions as H3K4me3 or H3K27ac interacting regions.
A positive feature of this study is the use of two different tissue types. This allowed for both estimation of reproducibility of the interactions as well as identification of some tissue-specific changes. The authors go through several broad characterizations of peak number and interaction frequency within versus between chromosomes. I also appreciated the use of 4C to validate interactions. I do have a slight issue with the presentation of this data. Figures 1d and S4 are attempting to show the correspondence of the ChIA-PET data and the 4C data. However, the specific interactions are not shown in this view (there is simply PET peaks and 4C signal), the specific location of the gene is not shown well (what is the orientation and boundaries of the gene?) and it is difficult to assess the validation. I would encourage the authors to find a better visualization to represent this data and the assertions they are making in the text.

The authors focus on the H3K27ac peaks for the study of "enhancers". They assess other chromatin marks and note increased accessibility and decreased DNA methylation for these regions. It is not unexpected for regions decorated with histone acetylation to be accessible and have reduced DNA methylation. They assessed the presence of TEs in these regions and find many examples of H3K27ac regions within TEs. They argue that these are enriched for LTR and Helitron elements. This assertion would need statistical support as these are the most common TEs in the maize genome. They need to provide statistical support for the enrichment beyond expected values to make the claim of enrichment. In addition, I am curious about the power to detect TEs in the approaches the authors are using. Given the repetitive nature of these sequences and the reliance upon unique mapping reads what are the biases in identifying TEs with enhancers?
The authors then search for "super-enhancers", regions with multiple enhancers over 10kb. They identify several hundred of these and then attempt to use GO terms to support function of these. The use of GO enrichment analysis was not helpful for this section and not convincing.
The authors then attempt to define P-P, P-E and E-E interactions based on the antibodies (see my concern above about this). I would like to know how the annotations of "promoter" agree with genome annotation. How often are H3K4me3 peaks (or PET peaks) within 1kb of annotated TSSs and how often are they further away?

The authors report several intriguing observations but these are primarily descriptive and they do not seem to probe these in detail. For example, the finding that 40% of H3K27ac peaks interact with a gene that is not the closest gene (ie skipping of genes). How often do these skip syntenic genes?

Expressed genes? The finding of single H3K27ac regions sometimes interact with multiple H3K4me3 regions (and vice versa) is interesting. I was not convinced by the authors approach of simply naming several well-characterized maize genes with multiple H3K27ac interacting regions. I would be interested to know whether genes with multiple H3K27ac interacting regions have higher levels of tissue specificity than other genes. Similarly, for instances in which a single H3K27ac region interacts with multiple H3K4me3 peaks I would be interested in knowing whether the correlation of expression values across development is higher than for non-interacting genes. This may be shown in figure S8d but the source of data for this was not explained in the legend.

The authors then proceed to focus on links between interactions and expression. The use the term co-expressed to reflect correlations in expression level within a single tissue. I am much less interested in this concept and more interested in coordinate regulation. Are these genes co-expression in existing B73 developmental co-expression networks? The authors do investigate Shannon entropy as a proxy for tissue-specificity but I am less interested in whether the simple presence of H3K27ac indicates tissue-specificity than the question of whether interacting sites are increased for co-expression correlation. The authors express some surprise at the concept that some interactions occur at silent genes. Wouldn't we expect some regulatory regions to allow for the binding of repressors? In addition, the authors are basing all conclusions upon the presence of "enhancers" or "promoters" that are defined by single modifications.
The authors attempt to address the potential dynamic nature of the interactions (and their function) by identifying interactions that are specific to immature ear or shoot tissue. I liked this line of investigation. However, the data was not very supportive of the conclusions. I could not discern substantial trends to support tissue specific interactions in figure 6b. The authors do show two examples in figure 6c-d but it is not surprising that a handful of examples could be found. This does not mean that the observed interactions are causative.

The last section of the results begins with essentially a list of genes with known functions that have interactions. This was not helpful in making a broader case for the role of interactions and was not all that novel. At best this section was validation of the method and was repetitive of the 4C section. The authors did proceed to a network analysis (ChIN). I liked this idea but struggled to find the details on how this was constructed. The primary interpretation of the network was based on GO terms and this was not at all convincing.

In summary, this is a descriptive work with some interesting observations. In many places I was not convinced that the assertions of the authors in reference to "promoters" or "enhancers" were accurate. In addition, there is very little evidence to suggest the function of these interacting regions. In many places the authors rely on evidence or examples from a small number of genes to suggest function but this does not provide novelty as many of these interactions were previously known. This work could be greatly strengthened by more careful description and dissection of the results as well as connections to prior functional variation (eQTL, GWAS, etc) information.

Minor comments
I am curious about the low mapping rate? Based on table S1 you seem to have a mapping rate between 5-15%. Is this due to loss of multi-mapping regions, PCR artifacts or some other reason?

Line 127 – these are not H3K27ac binding sites, they are sites which are enriched in binding of the H3K27ac antibody

I was not at all convinced by the GO analysis in figure 5C – this should be supplemental or removed.

Reviewer #2 (Remarks to the Author):

The authors applied H3K4me3 and H3K27ac ChIA-PET and ChIP-Seq experiments in two maize tissues, immature ear and shoot. They described the chromatin interaction profiles and studied the promoter/enhancer interactions and their relationship with gene transcription and agronomic traits. By integrated analysis of ChIA-PET data and other Omics datasets, e.g. RNA-Seq, ChIP-Seq and ATAC-Seq, the authors not only reconfirmed several known enhancers of genes, discovered new findings in maize, which are similar with previous findings in human and mouse, but also provided new insights for understanding the relation between chromatin interactions and gene transcription regulation. Besides, the high resolution chromatin interactions map will also serve as good resources for the community. In general, I think the manuscript is worthy of publication.

Major issue:
The manuscript is quite long. I suggest some details that do not directly correlate with the main conclusion of this paper could be moved to supplementary or even deleted. On the other hand, the method section is too brief, especially the part how to analyze/compare the data, which should be enhanced.

Minor issues:
1) Suppl table 1 shows that immature ear H3K27ac ChIA-PET library only has ~100 million uniquely mapped PETs, much less than 150 million, which is not consistent with line 101. I wonder whether this relative low sequencing depth will bias the results that based on H3K27ac-related chromatin interactions.
2) How was FDR calculated for chromatin interactions?
3) For Fig1d and suppl figure 4, it should also mark the viewpoint in 4C experiments. Why do the blue rectangles not consistent with Normalized 4C signal?
4) In line 176, it should cite the paper which the methylation data is from.
5) It's better to show the percentage of different regions (TE, downstream 2Kb of gene body, gene body and other regions) in the genome, as a control.
6) In line 226, according to the definition of classification, are there any PET peaks that neither close to TSS nor overlapped with ChIP-Seq enhancers? What's the number and percentage?
7) In Fig5a, 5b, 5d, the numbers of gene pairs or genes in each group should be indicated.
8) There is a mistake in Fig 5c.
9) The manuscript needs language editing, e.g. In line 362 ,"tissue-specific expression genes" should be "tissue-specific expressed genes". "shoot-preferred" and "shoot-specific" are not consistent. In line 380, "71% and 82% these enhancers" should be "71% and 82% of these enhancers". In line 579, "assume then that " should be "assume that ". There are many other typos/grammar errors not shown here.
10) In the "The comparison of chromatin interactions between different tissues" section, the authors used FPKM=1 as cutoff to classify genes into 3 groups (ear genes, common and shoot genes). Whether will this arbitrary cutoff affect the result? Maybe the authors could try some statistical methods, such as DESeq2, to classify the genes and check if the trend in Fig6b is more obvious and significant.

Reviewer #3 (Remarks to the Author):

The manuscript by Li et al reported H3K4me3 and H3K27ac ChIA-PET analysis for 2 maize tissues. This, as well as the other one, is the first ground breaking paper and finally showed that plants have functional distal cis-regulatory elements that could serve as enhancers. I believe it should be published in high impact journal, but I don't agree with some of their interpretation. Here is my concerns, and I am happy to see the revised draft soon.

1. The definition of super enhancer. In mammalian cells, SE is 1) first functionally characterized, 2) then the observation of strong med1 and TF oct4, sox2 and nanog binding in those regions, also 3) associated with high DNaseI-Seq (or ATAC-Seq signal), as well as high K27ac signals as well as other histone marks. But in this plant study, they skipped the most vital part 1 and 2. Only used the histone (plants only has K27ac in the distal region, no K4me1) and ATAC-Seq signal to call SE. That could be very misleading given that plant histone code is very different.

2. The example of SE in Fig 2g demonstrated why their strategy is problematic. They found a gene Zm00001d006094 associated with a broad K27ac peak in one tissue (missing label there) but not in the other, and say that is a SE. That is a MADS gene. It should express in ear only, if it is involved in floral organ identify. In many plant gene body region, K27ac should be almost the same as K4me3 and K4me1. So if they have a broad K4me3 peak there, which is common for MADS genes, then they will have a broad K27ac peak. I am sure they can use a better example maybe a distal region far away from genes, has strong K27ac and ATAC-Seq, no K4me3/K4me1, low DNA methylation, good ChIA-PET read link back to a gene promoter.

3. Without further functional validation, I think we should not rush to call these super enhancer regions. It will not hurt the novelty of this paper if they only claim that is a long enhancer-like region that has some chromatin features similar to the mammalian SE.

4. The correlation between loop and gene expression is a bit problematic (and they didn't specify how they run the analysis). We know genes close to each other are more likely to be co-expressed. So if they use the chromosome/genome as random, the p-value will always be small because the loop regions are closer than the whole genome random. A common strategy is to randomly select gene pairs with similar distance, and we normally shift that loop 1000 times in the same chromosome. That will generate lots of random pairs with the same distance. But I think the observed correlation should be correct. Just need to do better control.

5. How to define tissue-specific loop. That is tricky for the K4me3 and K27ac ChIA-PET. In mammalian cells, pol2 is often paused in the promoter, and the promoter often interacts with multiple enhancers. So one can compare the loop in 2 different cell lines/tissues. In this paper, if one tissue lost K4me3 or K27ac in the region that is involved in looping in another tissue, we won't be pulling it down in the ChIA-PET, then of course it will be called as "tissue-specific" chromatin interaction. I can argue that the loop can still exist, just the histone mark is gone. So we need to be really careful how to interpret those. I will be more confortable calling tissue-specific loop if the anchor position still have the same histone mark, but just no PET there to support the interaction. Also better do some stat testing if the read number is sufficient.

6. The discussion is a bit off in the SE part, and the method section didn't explain how they did the ChIA-PET. No plant ChIA-PET has been reported so far. That is one reason why this paper is valuable. So I think they should provide a detail method. From our experience, the most important factors for a successful ChIA-PET are the EGS fixation and sonication time. I think the author can write a better protocol and let other researchers follow their footstep to do it in other organisms/tissues. it is good for them as well, more citation.

Reviewers' comments:

Reviewer #1 (Remarks to the Author):

Li et al describe a ChIA-PET dataset describing interactions between regions containing H3K4me3 or H3K27ac in maize. The primary goal of this work, documenting interaction among promoters or promoters and distal regulatory regions, is very important and will likely yield major insights into important variation for influencing agronomic traits. However, I have some concerns about the interpretations of this dataset.

Thank you for your time and great comments.

One major concern is the interpretation of the meaning of the presence of the two chromatin marks used in the study. The authors claim that H3K4me3 and H3K27ac are “well known histone marks of promoter and enhancer, respectively”. I am not convinced that this is true and the cited works are from mammalian systems. There are numerous differences in chromatin between plants and animals and we cannot simply assume that chromatin marks that specify regions in mammals will have the same meaning in plants. Work in maize and Arabidopsis suggests that both of these marks are actually enriched at both proximal and distal regulatory regions in plants. Neither of them are specific for regions. Indeed – the authors themselves report that 70% of intrachromosomal marks are in common for the two modifications. I would encourage the authors to reduce the use of promoter and enhancer terms in this work. Instead, they can simply focus on the interactions they find that are between regions proximal (such as <2kb) to TSSs and distal region. Alternatively, they could simply refer to their interacting regions as H3K4me3 or H3K27ac interacting regions.

Taken reviewer’s suggestion, we have renamed it as “enhancer-like regulatory regions” in line 168-171. We agree that the histone marks in plant may not be exactly the same as those in animal systems, although in general, they can have high level of similarity.

Up to now, there have been some reports about the identification of enhancer in plants, including *Arabidopsis*, rice and maize. And we also cited two reviews about enhancer in plants in background section in line 55-56. They showed that enhancers in plants are generally associated with H3 and H4 acetylation. Another article in maize also confirmed that the enhancer of B1 gene have enrichment of H3K27ac and H3K9ac, but no enrichment of H3K4me1. (Oka, R. et al. Genome-wide mapping of transcriptional enhancer candidates using DNA and chromatin features in maize. Genome Biol 18, 137 (2017).). So we defined H3K27ac peaks that were 2Kb away from transcriptional start sites of genes as “enhancer-like regulatory regions” in line170-172. There is no doubt that these “enhancer-like regions” need further experimental validations. And we found that genes interacting with those enhancer-like regulatory regions had higher expression level than those without interactions.

Since both H3K4me3 and H3K27ac are associated with regulatory regions that may interacted with gene regions, interactions that directly or indirectly interacted with gene regions will be

captured in both of our modifications-mediated ChIA-PET datasets. So there were lots of overlapped PETs peaks between two modifications-mediated ChIA-PET datasets. Although H3K27ac shows some enrichment at TSS relative to the whole gene body regions, we have found about 50% of H3K27ac peak locating in other regions. The distribution of H3K27ac also displayed an enrichment at TSS relative to the whole gene body region in mouse and human.

A positive feature of this study is the use of two different tissue types. This allowed for both estimation of reproducibility of the interactions as well as identification of some tissue-specific changes. The authors go through several broad characterizations of peak number and interaction frequency within versus between chromosomes. I also appreciated the use of 4C to validate interactions. I do have a slight issue with the presentation of this data. Figures 1d and S4 are attempting to show the correspondence of the ChIA-PET data and the 4C data. However, the specific interactions are not shown in this view (there is simply PET peaks and 4C signal), the specific location of the gene is not shown well (what is the orientation and boundaries of the gene?) and it is difficult to assess the validation. I would encourage the authors to find a better visualization to represent this data and the assertions they are making in the text.

Thank for your suggestions for visualization. We have supplemented the specific location and orientation of genes in our revision. The regions interacting with viewpoints in 4C, H3K27ac-ChIA-PET and H3K4me3-ChIA-PET libraries in each tissue represented by blue and red rectangles in Figure 1d and S4, but not simply PET peaks and 4C signal. And the validations by 4C were highlighted by orange bars.

The authors focus on the H3K27ac peaks for the study of “enhancers”. They assess other chromatin marks and note increased accessibility and decreased DNA methylation for these regions. It is not unexpected for regions decorated with histone acetylation to be accessible and have reduced DNA methylation. They assessed the presence of TEs in these regions and find many examples of H3K27ac regions within TEs. They argue that these are enriched for LTR and Helitron elements. This assertion would need statistical support as these are the most common TEs in the maize genome. They need to provide statistical support for the enrichment beyond expected values to make the claim of enrichment. In addition, I am curious about the power to detect TEs in the approaches the authors are using. Given the repetitive nature of these sequences and the reliance upon unique mapping reads what are the biases in identifying TEs with enhancers?

We have revised it in line 188-190. And we agreed that our method may have some biases in identifying TEs with enhancer-like regulatory regions. As reviewer pointed out, we had used only uniquely mapped reads in our study. As we have discussed it in discussion section in line 498-499, our approach had inevitably underestimated the actual number of TEs as enhancer-like regulatory regions.

The authors then search for “super-enhancers”, regions with multiple enhancers over 10kb. They identify several hundreds of these and then attempt to use GO terms to support function of these. The use of GO enrichment analysis was not helpful for this section and not convincing.

The term and method for identification of “super-enhancer” were first reported in mouse (Whyte, W.A. et al. Master transcription factors and mediator establish super-enhancers at key cell identity genes. Cell 153, 307-19, 2013). And the researchers have found that genes associated with SEs were often key cell identity genes. Even in animal system, the only defining feature of super-enhancer is the exceptionally high degree of enrichment of transcriptional activators or chromatin marks as determined by ChIP-seq (Pott, S. & Lieb, J.D. What are super-enhancers? Nat Genet 47, 8-12 (2015)).

Our study is not intended to refer the function of SE. In revision, we used “super-enhancer-like regulatory region” instead of more confirmative “super enhancer”. Using the method of identification of SE in mouse, we identified the super-enhancer-like regulatory regions in maize. Similar as the reported SEs in mouse, we found that super-enhancer-like regulatory regions were also associated a number of gene associated with potential cell identity in maize. The GO terms of genes associated super-enhancer-like regions in maize showed that these genes may have important developmental function in immature ear and shoot of maize.

The authors then attempt to define P-P, P-E and E-E interactions based on the antibodies (see my concern above about this). I would like to know how the annotations of “promoter” agree with genome annotation. How often are H3K4me3 peaks (or PET peaks) within 1kb of annotated TSSs and how often are they further away?

We defined “promoter PET peaks” in line 223-224, which were consistent with genome annotation: PET peaks within 2Kb around TSS of gene. There were 78% of H3K4me3 peaks within 2Kb of annotated TSSs in both tissues. About 70% of H3K4me3 peaks and PET peaks located within 1Kb of annotated TSSs in both tissues.

The authors report several intriguing observations but these are primarily descriptive and they do not seem to probe these in detail. For example, the finding that 40% of H3K27ac peaks interact with a gene that is not the closest gene (ie skipping of genes). How often do these skip syntenic genes? Expressed genes? The finding of single H3K27ac regions sometimes interact with multiple H3K4me3 regions (and vice versa) is interesting. I was not convinced by the authors approach of simply naming several well-characterized maize genes with multiple H3K27ac interacting regions. I would be interested to know whether genes with multiple H3K27ac interacting regions have higher levels of tissue specificity than other genes. Similarly, for instances in which a single H3K27ac region interacts with multiple H3K4me3 peaks I would be interested in knowing whether the correlation of expression values across development is higher than for non-interacting genes. This may be shown in figureS8d but the source of data for this was not explained in the legend.

Thank you for your suggestions.

（1） We downloaded the syntenic gene list from maizeGDB (https://www.maizegdb.org). There are 24,674 syntenic genes between maize and sorghum. There were about 65% of these long-range P-E interactions skipping expressed genes, and 85% of these skipping syntenic genes, in both tissues.

（2） As you suggested, we compared the Shannon entropy of the genes interacting with different number of intergenic enhancer-like regulatory regions. The degree of tissue-specific expression of genes tended to increase as the number of intergenic enhancer-like PET peaks of these genes interacting with enlarged. The following figure has been added in Fig. 5e.

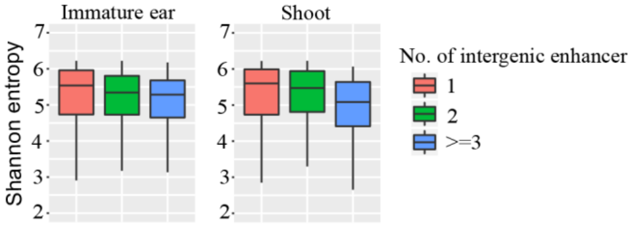

Summary the number of genes interacting with different number of intergenic enhancers

No. of E	Immature ear	Shoot
1	2,209	2,033
2	507	314
>=3	198	85

（3） As pointed in line 307-309 and Supplementary Fig. 8d, gene interacting with the same enhancer-like regulatory region had higher correlation level than randomly selected gene pairs (*p*-value < 2.2e-16, two-sided t-test).

（4） The source of data for Figure 5d and S8d were explained in the Supplementary Methods (Calculation of Shannon entropy), and we moved it to Methods section. We also added it in corresponding legends as you suggested.

The authors then proceed to focus on links between interactions and expression. The use the term co-expressed to reflect correlations in expression level within a single tissue. I am much less interested in this concept and more interested in coordinate regulation. Are these genes co-expression in existing B73 developmental co-expression networks? The authors do investigate Shannon entropy as a proxy for tissue-specificity but I am less interested in whether the simple presence of H3K27ac indicates tissue-specificity than the question of whether interacting sites are increased for co-expression correlation. The authors express some surprise at the concept that some interactions occur at silent genes. Wouldn't we expect some regulatory regions to allow for the binding of repressors? In addition, the authors are basing all conclusions upon the presence of "enhancers" or "promoters" that are defined by single modifications.

(1) We investigated the co-expressed degree of genes pairs with interactions in the collected RNA-Seq dataset in our lab, consisting of 53 different seed and 25 non-seed samples in maize (Chen, J. et al. Dynamic transcriptome landscape of maize embryo and endosperm development. Plant Physiol 166, 252-64 (2014).), not in a single tissue. The results showed that genes with chromatin interactions had higher co-expression level than those without chromatin interactions. So the method and principle of the construction of developmental co-expression networks were similar with us.

(2) We have downloaded the existing B73 development co-expression networks constructed by

Huang J *et al.* (Huang, J., Vendramin, S., Shi, L. & McGinnis, K.M. Construction and Optimization of a Large Gene Coexpression Network in Maize Using RNA-Seq Data. Plant Physiol 175, 568-583 (2017)). We found some of these gene pairs (~10%) were in existing B73 developmental co-expression networks. Gene pairs with chromatin interactions were not always the gene pairs with highest co-expression level in existing B73 developmental co-expression networks. To be note that 50% of co-expression gene pairs identified in existing B73 developmental co-expression networks were located in different chromosome. We only used intra-chromosomal interactions considering the high false positive of inter-chromosomal interactions in this paper.

(3) Based on our data, we cannot directly answer the question that whether interacting sites are increased for co-expression correlation in this current study. To fully answer this question, we think that we should have the dataset of ChIA-PET and RNA-Seq of series of tissues or different periods. Suppose that in series of tissue A, we found some gene pairs always with chromatin interactions. And at the same time, in series of tissue B, we found those gene pairs always without chromatin interactions. So we can compare the co-expression level of those gene pairs in series of tissue A and series of tissue B to investigate whether interacting sites are increased for co-expression correlation. We guess that may be true.

(4) Since we used H3K4me3 and H3K27ac enriched chromatin interactions. So these interactions at silent genes had active marks. We do not know whether these regions had other repressors. Previous studies in animal showed that these interactions were not enough for the expression of gene. The constructions of these chromatin interactions were before the activation of gene expression in the development. And the transcriptional activation of these silent genes may need other tissue-specific transcription factors.

(5) The definition of "promoters" in this paper was based on genome annotation, not by modification. We have redefined distal H3K27ac peaks as "enhancer-like regulatory regions" as you suggested.

The authors attempt to address the potential dynamic nature of the interactions (and their function) by identifying interactions that are specific to immature ear or shoot tissue. I liked this line of investigation. However, the data was not very supportive of the conclusions. I could not discern substantial trends to support tissue specific interactions in figure 6b. The authors do show two examples in figure 6c-d but it is not surprising that a handful of examples could be found. This does not mean that the observed interactions are causative.

Yes, the original Fig. 6b was not so clear in spite that being supported by significant statistical difference. Since we used materials with mixed cell types and the criterion for the identification of tissue-specific expressed gene was relatively loose, there may be much noise affecting the results. As suggested by another reviewer, we have adopted a more stringent criterion to identify tissue-specific expressed genes in our revision. If one gene had FPKM =0 in one tissue and had FPKM >=2 in the other tissue, then the gene was identified as a tissue-specific expressed gene in the latter tissue. We then identified 286 immature ear-specific expressed genes and 675 shoot-specific expressed genes. And the trend in Fig. 6b was more obvious and significant. The statistical test showed significant difference, supporting that tissue-specific expressed genes and tissue-specific interactions were indeed associated. We also give more detail description in the

revised Methods section (The analysis of tissue-specific expressed genes and chromatin interactions).

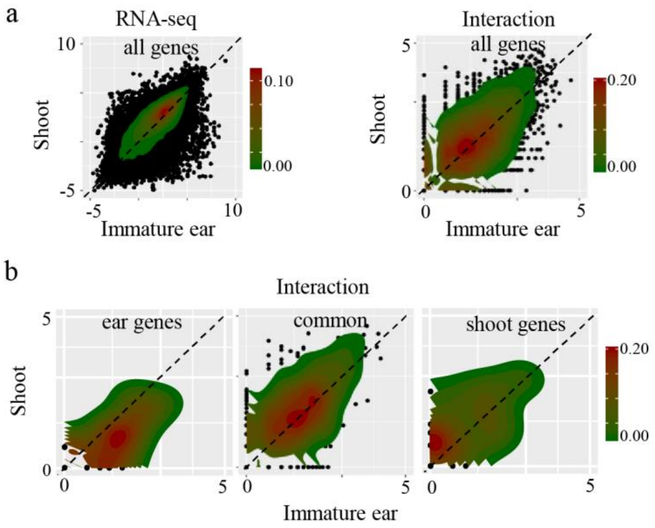

The last section of the results begins with essentially a list of genes with known functions that have interactions. This was not helpful in making a broader case for the role of interactions and was not all that novel. At best this section was validation of the method and was repetitive of the 4C section. The authors did proceed to a network analysis (ChIN). I liked this idea but struggled to find the details on how this was constructed. The primary interpretation of the network was based on GO terms and this was not at all convincing.

We have added the details for the construction of ChINs in line 707-710 in Methods (Chromatin interaction network analysis and visualization). The nodes in ChINs present PET peaks in chromatin interactions. Each edge in ChINs presents corresponding chromatin interaction. All chromatin interactions in each tissue was used to construct corresponding ChINs. Nodes that interacted directly or indirectly consist of a ChIN component.

Genes in the same chromatin network component interacted directly or indirectly and were co-expressed. So we think they may be in the same or related biological process. Before we can have other evidences, gene ontology analysis anyway is the easy validation we can have, the same analysis also has been used in human.
(Sandhu, K.S. et al. Large-scale functional organization of long-range chromatin interaction networks. Cell Rep 2, 1207-19 (2012).)

In summary, this is a descriptive work with some interesting observations. In many places I was not convinced that the assertions of the authors in reference to “promoters” or “enhancers” were accurate. In addition, there is very little evidence to suggest the function of these interacting regions. In many places the authors rely on evidence or examples from a small number of genes to suggest function but this does not provide novelty as many of these interactions were previously known. This work could be greatly strengthened by more careful description and dissection of the results as well as connections to prior functional variation (eQTL, GWAS, etc) information.

Thank you for your time and interests and great comments on our manuscript. We redefined these regions as “promoter-like” and “enhancer-like” regulatory regions.

To connect our interaction data with other eQTL and GWAS results as you suggested, we detected eGWAS signals in the enhancer regions we identified for their interacting genes, and GWAS signals in ChINs related to *Dwarf8* for plant height and flowering time. However, since the eGWAS and GWAS results are involving large amount of unpublished data (some of them even from other labs). In the same time, these results need further validation. Therefore, these results are not included in this manuscript.

Below are our integrated results for your information:

(1) By using the eGWAS data in our lab (unpublished data), we compared the *p*-values of SNPs located in corresponding interacting enhancer-like PET peaks with those of SNPs in regions not interacting with genes (background), for genes interacting enhancer-like PET peaks. We got 57,762 and 61,312 SNPs in corresponding interacting enhancer-like PET peaks for 3,071 and 2,962 genes in immature ear and shoot, respectively. And 60,000 randomly selected SNPs in background were as control in both tissues.

There were significant higher association signals in the interacting enhancer-like PET peaks for the *p*-value of SNP of genes from genome-wide expression quantitative trait loci (eQTL) analysis, compared with background, in both immature ear and shoot. This result showed that those corresponding enhancer-like PET peaks were closely associated with the expression level of genes.

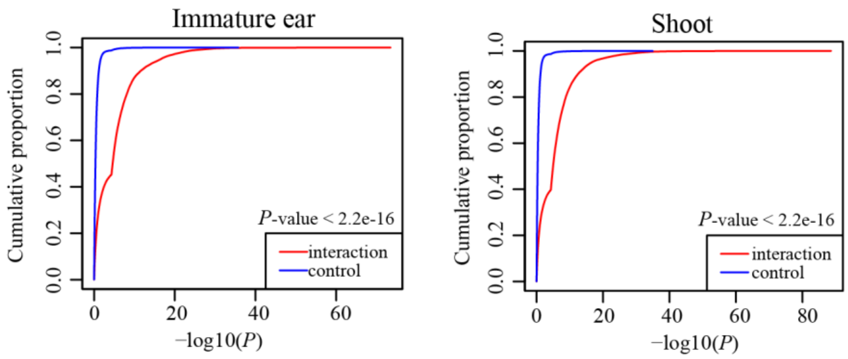

(2) We used another GWAS data (unpublished data) and Yang, N. et al. to dissect the ChIN component of *Dwarf8*. We compared the *p*-values of SNPs of plant height, days to anthesis (DA) and days to silking (DS) in the regions of the ChIN component of *Dwarf8* with those of SNPs in remaining regions not in the ChIN component of *Dwarf8* (background).

(Yang, N. et al. Genome wide association studies using a new nonparametric model reveal the genetic architecture of 17 agronomic traits in an enlarged maize association panel. PLoS Genet 10, e1004573 (2014).)

From genome-wide association study (GWAS) analyses in our lab, we got 4,703 SNPs in the ChIN component of *Dwarf8* and 5,000 randomly selected SNPs in background were as control for each trait. To be note, the genotype in our lab used in GWAS was from whole-genome resequencing, and the majority of SNPs (84%) were in intergenic regions. The *p*-value of SNPs of plant height, days to anthesis (DA) and days to silking (DS) in ChIN

component of *Dwarf8* showed significant higher association signals with corresponding traits than background, implying that this ChIN were associated with both plant height and flowering time.

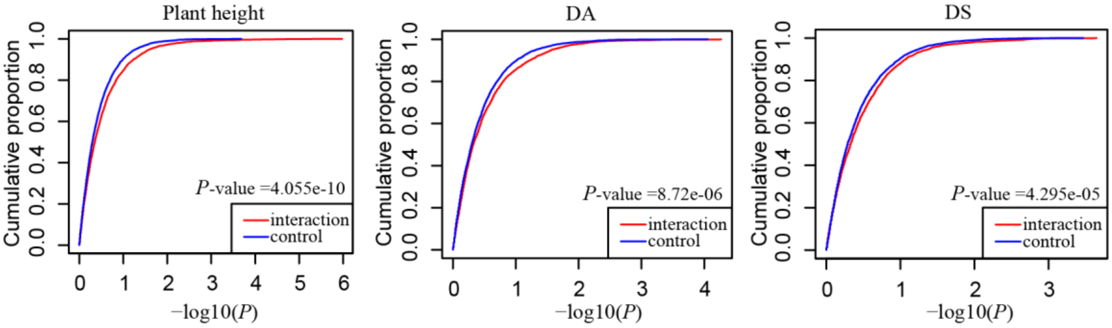

From genome-wide association study (GWAS) analyses in Yang, N. et al. (published data), we got 9,669 SNPs in the ChIN component of *Dwarf8* and 10,000 randomly selected SNPs in background were as control for each trait. To be note, the genotype in Yang, N. et al used in GWAS was from RNA-seq, so the majority of SNPs (85%) were in genic regions. The *p*-value of SNPs of plant height, silking time in ChIN component of *Dwarf8* also showed significant higher association signals with corresponding traits than background. Despite that signal of heading date was not as strong as plant height and silking time this ChIN component, it also showed association with this trait. Lack of intergenic SNPs may contribute to the relative lower signal in this ChIN component.

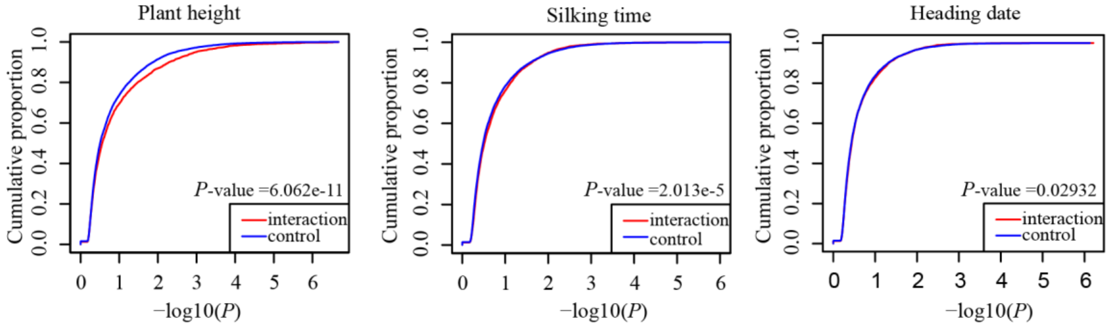

Minor comments

I am curious about the low mapping rate? Based on table S1 you seem to have a mapping rate between 5-15%. Is this due to loss of multi-mapping regions, PCR artifacts or some other reason?

The overall alignment rate was about 95%. The uniquely mapping rate was between 40-45%. Here shows the uniquely mapped PETs were after filtering the reads without bridge linkers, multi-mapped and unpaired reads. We have modified the table by adding more details.

Line 127 – these are not H3K27ac binding sites, they are sites which are enriched in binding of the H3K27ac antibody

We have corrected it in line 127-130.

I was not at all convinced by the GO analysis in figure 5C – this should be supplemental or removed.

Thank you for your suggestion. We have moved it to Supplemental Figure 9b.

Reviewer #2 (Remarks to the Author):

The authors applied H3K4me3 and H3K27ac ChIA-PET and ChIP-Seq experiments in two maize tissues, immature ear and shoot. They described the chromatin interaction profiles and studied the promoter/enhancer interactions and their relationship with gene transcription and agronomic traits. By integrated analysis of ChIA-PET data and other Omics datasets, e.g. RNA-Seq, ChIP-Seq and ATAC-Seq, the authors not only reconfirmed several known enhancers of genes, discovered new findings in maize, which are similar with previous findings in human and mouse, but also provided new insights for understanding the relation between chromatin interactions and gene transcription regulation. Besides, the high resolution chromatin interactions map will also serve as good resources for the community. In general, I think the manuscript is worthy of publication.

Major issue:

The manuscript is quite long. I suggest some details that do not directly correlate with the main conclusion of this paper could be moved to supplementary or even deleted. On the other hand, the method section is too brief, especially the part how to analyze/compare the data, which should be enhanced.

Thank you for your time and great comments on our manuscript. We have moved Fig. 5e to Supplemental Fig. 9b. And we have supplemented the methods in detail, including the detailed parameters and procedures of ChIA-PET, the identification of high-confidence chromatin interaction, comparison of data and the construction of chromatin networks.

Minor issues:

1) Suppl table 1 shows that immature ear H3K27ac ChIA-PET library only has ~100 million uniquely mapped PETs, much less than 150 million, which is not consistent with line 101. I wonder whether this relative low sequencing depth will bias the results that based on H3K27ac-related chromatin interactions.

Thank you for your suggestions. We have modified it in line 104. We understand the reviewer's concern that the relative low sequencing depth of immature ear H3K27ac ChIA-PET library may underestimated the H3K27ac-related chromatin interactions identified in immature ear. But we don't think this affect the overall conclusion in this paper. And we didn't find significantly different number of chromatin interaction between H3K27ac ChIA-PET libraries in different tissues (21,110 H3K27ac-related high-confidence interactions were identified in immature ear and 23,255 H3K27ac-related chromatin interaction were identified in shoot).

2) How was FDR calculated for chromatin interactions?

The detailed algorithm for the calculation of FDR has been supplemented in line 645-652 in Methods (Identification of chromatin interactions).

3) For Fig. 1d and Supplementary Fig. 4, it should also mark the viewpoint in 4C experiments. Why do the blue rectangles not consistent with Normalized 4C signal?

Thank you for your suggestions and thorough review. We have supplemented the specific location of genes or enhancer as viewpoints. I apologized for the mistakes I made in Fig. 1d and Supplementary Fig. 4. I plotted the normalized 4C signal using only one replicate in 4C data and the blue rectangles were identified by combined data with two replicates. The mistake has been corrected. We used the average of normalized 4C signal between replicates in 4C data. 4C interactions identified in both two replicates were showed here.

4) In line 176, it should cite the paper which the methylation data is from.

We have supplemented it in line 178 in our revised manuscript.

5) It's better to show the percentage of different regions (TE, downstream 2Kb of gene body, gene body and other regions) in the genome, as a control.

Thank you for your suggestions. We have supplemented the percentage of different regions (TE, downstream 2Kb of gene body, gene body and other regions) in the genome in Fig. 2d.

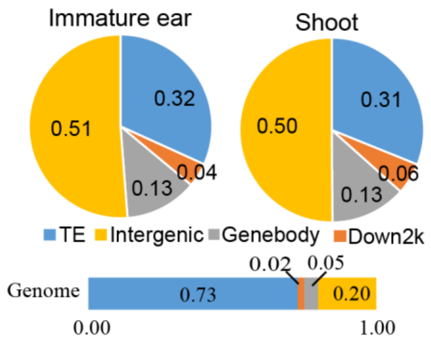

6) In line 226, according to the definition of classification, are there any PET peaks that neither close to TSS nor overlapped with ChIP-Seq enhancers? What's the number and percentage?

Yes. There were 473 and 777 PET peaks, about 2~3% of all PETs peaks, that neither close to TSS nor overlapped with ChIP-Seq enhancers we identified in immature ear and shoot, respectively.

7) In Fig. 5a, 5b, 5d, the numbers of gene pairs or genes in each group should be indicated.

Thank you for your suggestions. We have supplemented the numbers of gene pairs or genes in each group in Supplementary Table 7, which was indicated in the legend of Fig. 5.

As suggested by another reviewer, we used a better control in Fig. 5a by randomly shifting the interaction 100 times in the same chromosome to generate lots of random pairs with the same distance. The detailed method was supplemented in Methods (Comparison of expression correlation for gene pairs with chromatin interactions with control).

Supplementary Table 7. Summary of the numbers of gene pairs or genes in Figure 5.

		Immature ear	shoot
Figure 5a.	P-P	6,333	6,182
	control	37,080	41,677
Figure 5b.	with interaction	21,989	23,246
	without interaction	17,367	16,125
Figure 5d.	Gene interacting with intergenic enhancer	8,283	7,287
	Gene interacting with genic enhancer	846	1,175
	Gene with enhancer in its gene body	1,933	2,724

8) There is a mistake in Fig. 5c.

It is corrected.

9) The manuscript needs language editing, e.g. In line 362, "tissue-specific expression genes "should be "tissue-specific expressed genes". "shoot-preferred" and "shoot-specific" are not consistent. In line 380, "71% and 82% these enhancers" should be "71% and 82% of these enhancers". In line 579, "assume then that" should be "assume that". There are many other typos/grammar errors not shown here.

A consistent use of terms has been applied in line 373-403 and the legend of Fig. 6b in our revised manuscript. And the typos/grammar errors have been corrected.

10) In the "The comparison of chromatin interactions between different tissues" section, the authors used FPKM=1 as cutoff to classify genes into 3 groups (ear genes, common and shoot genes). Whether will this arbitrary cutoff affect the result? Maybe the authors could try some statistical methods, such as DESeq2, to classify the genes and check if the trend in Fig6b is more obvious and significant.

Thank you for your suggestions. We chose this cutoff to get genes with stringently different transcriptional state in different tissues: expressed gene and non-expressed gene, but not genes with different expression levels. In fact, we had tried DEGs (differently expressed genes) identified by Cuffdiff, a software similar to DESeq2, in Fig. 6b, and the trend in Fig. 6b was reduced. Note that DEGs identified by Cuffdiff or DESeq2 were expressed gene that have

significant expression level between different tissues.

We have adopted a more stringent criterion to identify tissue-specific expressed genes. If one gene had FPKM =0 in one tissue and had FPKM >=2 in the other tissue, then the gene was identified as a tissue-specific expressed gene in the latter tissue. There were 286 immature ear-specific expressed genes and 675 shoot-specific expressed genes. And the trend in Fig. 6b was more obvious and significant. The detail method for this figure was also supplemented in Methods (The analysis of tissue-specific expressed genes and chromatin interactions).

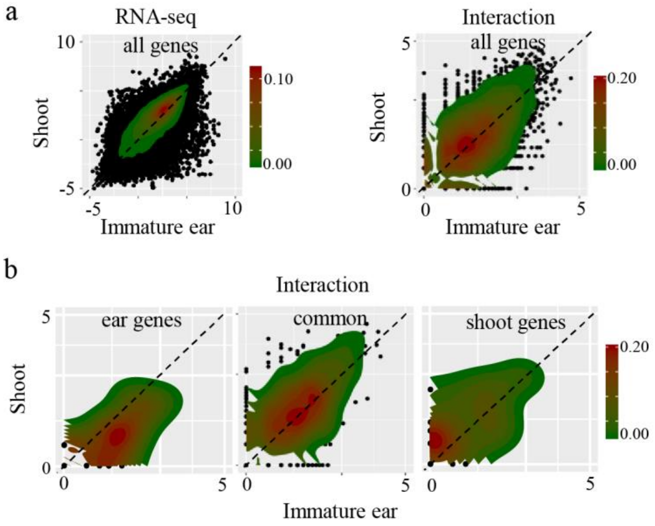

Reviewer #3 (Remarks to the Author):

The manuscript by Li et al reported H3K4me3 and H3K27ac ChIA-PET analysis for 2 maize tissues. This, as well as the other one, is the first ground breaking paper and finally showed that plants have functional distal cis-regulatory elements that could serve as enhancers. I believe it should be published in high impact journal, but I don't agree with some of their interpretation. Here is my concerns, and I am happy to see the revised draft soon.

1. The definition of super enhancer. In mammalian cells, SE is 1) first functionally characterized, 2) then the observation of strong med1 and TF oct4, sox2 and nanog binding in those regions, also 3) associated with high DNaseI-Seq (or ATAC-Seq signal), as well as high K27ac signals as well as other histone marks. But in this plant study, they skipped the most vital part 1 and 2. Only used the histone (plants only has K27ac in the distal region, no K4me1) and ATAC-Seq signal to call SE. That could be very misleading given that plant histone code is very different.

We understand the reviewer's concern about the function of SEs we identified. We have modified and termed it as "super-enhancer-like region".

The term and method for identification of "super-enhancer" were first reported in mouse (Whyte, W.A. et al. Master transcription factors and mediator establish super-enhancers at key cell identity

genes. Cell 153, 307-19 (2013)). The resulting super-enhancers by this method are not quite defined functionally. The only defining feature of super-enhancer is an exceptionally high degree of enrichment of transcriptional activators or chromatin marks as determined by ChIP-seq (Pott, S. & Lieb, J.D. What are super-enhancers? Nat Genet 47, 8-12 (2015)).

As you pointed, the author used ChIP-Seq datasets for Med1, Oct4, Sox2 and Nanog (OSN) to identified SEs in murine ESCs, since master transcription factors Oct4, Sox2 and Nanog bind enhancer elements and recruit Mediator to activate much of the gene expression program of pluripotent embryonic stem cells (ESCs). Mediator complex facilitates these transcription factors to recruit genes, and is essential for the maintenance of ESC state. ESCs are highly sensitive to reduce levels of Mediator. So authors were particularly interested in Mediator and used the enrichment of Med1 to distinguish enhancer and super-enhancer.

Results from ESC cell have compared the relative powers of ChIP-Seq data for OSN, Mediator, H3K27ac, H3K4me1, as well as DNaseI hypersensitivity data to distinguish super-enhancers from typical enhancers. They indicated that all these marks individually can pick up super-enhancers. The SEs are simply resulted from the highly clustered enhancers.

Enhancers in plants are generally associated with H3 and H4 acetylation. Another article in maize also confirmed that the enhancer of B1 gene have enrichment of H3K27ac and H3K9ac, but no enrichment of H3K4me1. So we used H3K27ac for enhancer-like regulatory regions identification. The clustered enhancer-like regions are called as super-enhancer-like regulatory regions.

2. The example of SE in Fig 2g demonstrated why their strategy is problematic. They found a gene Zm00001d006094 associated with a broad K27ac peak in one tissue (missing label there) but not in the other, and say that is a SE. That is a MADS gene. It should express in ear only, if it is involved in floral organ identify. In many plant gene body region, K27ac should be almost the same as K4me3 and K4me1. So if they have a broad K4me3 peak there, which is common for MADS genes, then they will have a broad K27ac peak. I am sure they can use a better example maybe a distal region far away from genes, has strong K27ac and ATAC-Seq, no K4me3/K4me1, low DNA methylation, good ChIA-PET read link back to a gene promoter.

To identify super-enhancer-like regulatory regions, we identified H3K27ac peaks which located 2Kb far away from TSS as enhancer-like regulatory regions. These enhancer-like regulatory regions always had strong H3K27ac and ATAC-Seq, low H3K4me3 and low DNA methylation. These enhancer-like regulatory regions within 12.5Kb were stitched together to identify super-enhancer-like regulatory regions (See Methods for details).

Fig. 2g showed unrelated (separated) example of super-enhancer and enhancer both in immature ear. The super-enhancer and enhancer displayed in Fig. 2g are located in different regions of genome. Our purpose of this figure was to display the significantly different lengths between SE and E. Since the super-enhancers tend to overlap with genes (these genes are within the SE regions) with high expression level, which typically have high H3K27ac and H3K4me3 level. The enhancer we showed here are indeed away from gene.

3. Without further functional validation, I think we should not rush to call these super enhancer regions. It will not hurt the novelty of this paper if they only claim that is a long enhancer-like regulatory region that has some chromatin features similar to the mammalian SE.

We have modified and termed it as “super-enhancer-like regulatory region”.

4. The correlation between loop and gene expression is a bit problematic (and they didn’t specify how they run the analysis). We know genes close to each other are more likely to be co-expressed. So if they use thechromosome/genome as random, the p-value will always be small because the loop regions are closer than the whole genome random.A common strategy is to randomly select gene pairs with similar distance, and we normally shift that loop 1000 times in the same chromosome. That will generate lots of random pairs with the same distance. But I think the observed correlation should be correct. Just need to do better control.

Thank you for your suggestions. We have taken you advice and added the detail in Methods. We randomly shifted the loop 100 times in the same chromosome to generate lots of random pairs with the same distance. As you expected, the observed correlations were correct.

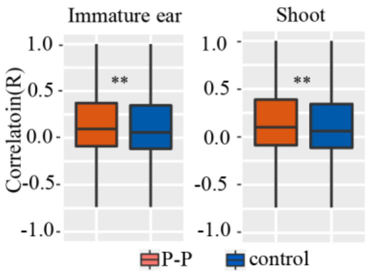

5. How to define tissue-specific loop. That is tricky for the K4me3 and K27ac ChIA-PET. In mammalian cells, pol2 is often paused in the promoter, and the promoter often interacts with multiple enhancers. So one can compare the loop in 2 different cell lines/tissues. In this paper, if one tissue lost K4me3 or K27ac in the region that is involved in looping in another tissue, we won’t be pulling it down in the ChIA-PET, then of course it will be called as “tissue-specific” chromatin interaction. I can argue that the loop can still exist, just the histone mark is gone. So we need to be really careful how to interpret those. I will be more confortable calling tissue-specific loop if the anchor position still has the same histone mark, but just no PET there to support the interaction. Also better do some stat testing if the read number is sufficient.

Thank you for your suggestions. We found that about 99% tissue-specific chromatin interactions we identified had H3K4me3 or H3K27ac anchor sites in both immature ear and shoot. In immature ear, there were 10,958 (99.5%) ear-specific chromatin interactions with H3K4me3 or H3K27ac anchor sites in both tissues. In shoot, there were 12,087 (98.9%) shoot-specific chromatin interactions with H3K4me3 or H3K27ac anchor sites in both tissues. We identified chromatin interaction in relatively strict statistic test and standards, and the identification of tissue-specific chromatin interaction was based on that.

6. The discussion is a bit off in the SE part, and the method section didn't explain how they did the ChIA-PET. No plant ChIA-PET has been reported so far. That is one reason why this paper is valuable. So I think they should provide a detail method. From our experience, the most important factors for a successful ChIA-PET are the EGS fixation and sonication time. I think the author can write a better protocol and let other researchers follow their footstep to do it in other organisms/tissues. it is good for them as well, more citation.

We have rephrased our discussion. We have complemented the details for ChIA-PET in Supplemental Methods.

Reviewers' comments:

Reviewer #1 (Remarks to the Author):

Li et al have revised the manuscript and some section have improved. This manuscript does report a very interesting and useful dataset. There are essentially three main issues I have with the work.

1. I continue to have significant issues with the authors use of the terms promoter and enhancer. These should be reduced substantially. The authors should either refer to H3K4me3 or H3K27ac interaction peaks or proximal/distal interactions rather that promoter/enhancer. My suggested terms describe exactly what has been shown while the authors terms imply a function for which they do not have experimental support. I make a number of comments on this below.

2. The key findings of this paper should be made available to the community. Many other researchers will be interested in the H3K27ac and K3k4m3 peaks and interactions. The authors need to make tables of these regions available (or bed files) that can be used by other researchers to further investigate these regions. These supplemental datasets will be very useful.

3. The writing needs substantial improvement. I noted some issues but there are many more…

Specific comments:
Line 49: Rewrite as "Enhancers, important regulatory elements, play important role sin modulating tissue specificity as well as the level of gene expression."

Line 51 – replace 'markers' with 'modifications'

Line 52: replace 'proved' with 'shown'

Line 59, make enhancer and promoter plural

Line 60: add 'a' so it reads "often form a DNA loop"

Line 66: change 'moderate' to 'moderately'

Line 66: add 'are' so it reads "and are often…"

Line 68, make interaction plural

Line 70-71:replace protein of interest with "chromatin modification of interest" since this study is monitoring a modification not a protein.

Line 76: change to "similar to animals"

Line 85: These are not shown to be typical marks of promoter and enhancer. Instead, they are chromatin modifications enriched at putative enhancers and promoters.

Line 86: These are not promoter-centered and enhancer-centered maps, they are H3K4me3 and H3K27ac centered maps and should be referred to as such

Line 89 replace 'prone to' with 'enriched for' and add genes at the end of the sentence

Line 101: The reference cited here are for animal species. Do not describe these as "well-known histone mores of active promoters and enhancer". Instead refer to these are histone modifications

that are enriched at active promoters and enhancers in other species.

Line 126: I like this phrasing – simply refer to the peaks as H3K4me3 related interaction peaks. This is much more accurate than calling them promoters (or enhancers). Indeed – this section shows the flaws in calling these promoter or enhancer peaks. A large number of them are enriched at the 3' end of genes which is not the promoter and both H3K4me3 and H3K27ac are enriched at the TTS.

Line 141 and figure 1d:Exactly where is the viewpoint in this figure? This needs to be indicated. The figure should also show the location of H3K27ac and H3K4me3 ChIP peaks.

Line 165: These lists of long range peaks (and which dataset they are found in) should be provided as a supplemental table or deposited somewhere that other researchers can access.

Line 170-171. Do not refer to these as enhancer-like. Simply refer to these as H3K27ac peaks. This is more precise and is accurate. Unless you are going to provide data to show that these regions act as enhancers, or even that a decent number have enhancer activity it is not appropriate to refer to them as a functional unit.

Line 173: Report these full sets as a supplemental table or supplemental dataset

Line 180: Add the details (CG and dataset) to the figure legend.

Line 205-219 are really dry. Simply summarize the terms that are enriched and if you want to refer to the genes put this in a table. I don't need a list of gene IDs in the text.
Lines 222-225: Again, I am not convinced that the authors should use the terms promoter and enhancer. I would simply recommend using promixal and distal. They can keep the current analyses but simply call them P and D as these refer to location, not putative functions. These lists (and classifications) should be reported in a supplemental table or dataset.

Lines 236-251 are simply summarizing numbers, many of which are better supported by the figures and I would suggest shortening this section.

Lines 253-256 seem entirely redundant with lines 259. Please simplify this text.

Lines 268-309 provide a discussion of the relationship of proximal regions to distal regions and provide some nice examples. I do not have any issues with this data but this section could be significantly shortened without losing clarity or impact.

Line 320 This data does not show co-expression, it simply shows correlation. Co-expression is usually reserved to describe patterns across samples, not correlation of levels within a sample. On a broader note, II am not convinced that this result is worth investigating. I am much more interested in the question of whether two interacting genes are tend to exhibit co-expression throughout development. I suspect that the expression levels are not actually correlated. I suspect this result is simply driven by the fact that any two genes with PET peaks are enriched for expression (5b). This means your interactions are enriched for "on" genes with random permutations find many on-off pairs.

Line 329-332 I suspect many of these genes simply do not have distal interactions, it is likely that for many maize genes the necessary regulatory elements are within several kb of the promoter.
Line 345 'against as expected' is not the correct phrasing

Line 345 What expression data is being used for this analysis? This needs to be included in the figure legends in S10 and 5.

Line 352 In figure 5d the control of genes without interactions with distal regions should be included

Line 366 I am not convinced this is true unless this is demonstrated relative to genes without PET peaks. All the authors have demonstrated currently is that genes with intergenic interactions are more tissue-specific than genes with interactions with genic regions.

Line 384-387 Are these distant interactions often supported by fewer reads that closer interactions? If so, the difference among tissues might simply be due to more difficult in supporting weaker interactions.

Line 407-428 many portions of this text are redundant with results already presented. This section should be substantially shortened.

Line 431 The ChIN should be made available to other researchers in some format. This could include depositing the edges of the network or making a browser for the network.

Line 430-473 I can understand the potential value of the network and the idea that genes within a set of interacting partners might have related function. However, this section is not written well and I struggled to find the specific points that authors are trying to make.

Line 477-478 I continue to have issues with the term enhancer. It is better to simply refer to these as distal regulatory regions. In some cases the interactions may actually be related to repression rather than activation.

Line 485: "super long distance" is probably not an appropriate term

Line 506-507 I would argue that your terminology is actually going to create confusion.

I think it is critical for the authors to provide a table with a full list of the coordinates of the PET peaks. These could indicate which peaks are found in both datasets and have different lists for each tissues. In addition, the full list of ChIP peaks should be reported. These files will be used by other researchers.

Reviewer #2 (Remarks to the Author):

I'm satisfied with the revised manuscript and suggest NC to publish.

Reviewer #3 (Remarks to the Author):

The revised manuscript is better. As I said last time, I think this should go to high impact journals. But it still has some errors when performing the analysis and interpreting the data. I list them below.

Line 133, In order to claim that high K4me3 or K27ac regions are enriched for long rang interaction, one needs to use the total reads (not just ChIA-PET cis-interaction PETs) in each peaks, plot them against the PET number. They can also plot the region's K4me3 or K27ac chip-seq signal against the PET number.

L170, I would suggest to say K27ac is a mark of active enhancer in mammalian model.

L200, 218-219, I still do not believe those are super enhancer or SE-like regions (simply based on the length of the K27ac peak). If they are so determined to claim this, please find those enhancers and

see where they are, I bet some of them are located in poorly assembled regions, they often have higher sequencing coverage, and often called as long peak in other histone ChIP-Seq experiments unrelated to K27ac. Or maybe just look at the K4me3 data, they might have the same long K4me3 marks. Also, floral MADS genes with long first introns are known to have long K27ac peaks inside their gene. Should we call all of them SE? I hope they can do some more analysis to show the feature of those so-called SE-like regions, and maybe include them into Sup Info. If they don't want to do that, fine. Please write the discussion and result more carefully, otherwise they might regret making those strong statements in the future when new evidences have emerged showing those are not SE.

L222-224. This is a wrong strategy to annotate PET. PET 2kb away could still be a promoter to promoter interaction (e.g. an unannotated gene is there). K4me3 PET might also be an interaction between a promoter (K4me3) and a distal enhancer (region without K4me3). So they should classify them using the method in the co-summitted paper, in which promoter-promoter interaction are identified as those PET with both anchor sites associated with K4me3. The promoter to distal element PET could be defined as PET with one anchor site having K4me3 and the other anchor site having open chromatin only. Enhancer-enhancer PET will be 2 anchors associated with DHS, but no K4me3.

I am afraid that this will affect most of their following analysis, and many of them need to be re-run using the correction PET classification.

L263-264. This is an important observation!

L 318. They now used a random control made of gene pairs of the same genome distance. But it is still missing a key component. The random gene pairs should be associated with the same K4me3 mark or K27ac.

L 321, Again, control should be genes associated with K4me3 or K27ac depends on the ChIA-PET antibody. The 2nd control will be genes associated with the same K4me3 or K27ac ChIP-Seq signal. Because the higher the chip-seq signal, the more likely they will be identified from ChIA-PET. And we know highly expressed genes are associated with active histone marks.

L 332, What should be used as the proper control in the GO enrichment analysis? Maybe the genes most enriched with K4me3 / K27ac genes are associated with this, not the loop genes.

L 343. Again, they should not compare enhancer-PET genes to other non-E-PET ones. They need to find control(s). Say control group 1 could be those PET free genes with K4me3, g2 is the K27ac PET free genes. One should be very careful in making such claim…Look at the ChIP-Seq signal intensity of those genes. It might be the higher ones that got the PET, so another control will be genes associated with prompter-promoter interaction.

L348-. The English from here is hard to understand. They should be careful in defining the intergenic enhancer, and only use those without K4me3 in the "intergenic anchor region".

L363, please define "genes with enhancer-like PET peaks in gene bodies". Is it gene A is associated with PET, and the PET is from gene A to the body of gene B? If that is the case, what should be used as a control? Genome random is obviously wrong as discussed above. How about gene A associated PET with distal intergenic open chromatin? How about genes with the same K4me3 peaks but not associated with PET etc…

L389. "Tissue-specific enhancer-like PET peaks showed enrichments in tissue-specific P-E interactions." It doesn't make sense. Also, please use a statistic test with proper controls for making such statement.

L430-439. Genes with higher degree of interaction might just be the genes with higher K4me3 or K27ac ChIP-Seq signal. So you wants to claim that, please do a test with proper control.

Reviewers' comments:

Reviewer #1 (Remarks to the Author):

Li et al have revised the manuscript and some section have improved. This manuscript does report a very interesting and useful dataset. There are essentially three main issues I have with the work.

Thank you for your time and great comments.

1. I continue to have significant issues with the authors use of the terms promoter and enhancer. These should be reduced substantially. The authors should either refer to H3K4me3 or H3K27ac interaction peaks or proximal/distal interactions rather that promoter/enhancer. My suggested terms describe exactly what has been shown while the authors terms imply a function for which they do not have experimental support. I make a number of comments on this below.

Thank you for your suggestion. We have taken you advice and used proximal/distal interactions in our revision.

2. The key findings of this paper should be made available to the community. Many other researchers will be interested in the H3K27ac and K3H4me3 peaks and interactions. The authors need to make tables of these regions available (or bed files) that can be used by other researchers to further investigate these regions. These supplemental datasets will be very useful.

We had provided the H3K27ac and H3K4me3 peaks and interactions in Supplemental Table 4.

3. The writing needs substantial improvement. I noted some issues but there are many more…

Thank you for your suggestion. The manuscript has been revised carefully.

Specific comments:
Line 49: Rewrite as “Enhancers, important regulatory elements, play important roles in modulating tissue specificity as well as the level of gene expression.”

Corrected.

Line 51 – replace ‘markers’ with ‘modifications’

Corrected.

Line 52: replace ‘proved’ with ‘shown’

Corrected.

Line 59, make enhancer and promoter plural

Corrected.

Line 60: add ‘a’ so it reads “often form a DNA loop”

Corrected.

Line 66: change ‘moderate’ to ‘moderately’

Corrected.

Line 66: add ‘are’ so it reads “and are often…”

Corrected.

Line 68, make interaction plural

Corrected.

Line 70-71: replace protein of interest with “chromatin modification of interest” since this study is monitoring a modification not a protein.

Corrected.

Line 76: change to “similar to animals”

Corrected.

Line 85: These are not shown to be typical marks of promoter and enhancer. Instead, they are chromatin modifications enriched at putative enhancers and promoters.

Corrected.

Line 86: These are not promoter-centered and enhancer-centered maps, they are H3K4me3 and H3K27ac centered maps and should be referred to as such

Corrected.

Line 89 replace ‘prone to’ with ‘enriched for’ and add genes at the end of the sentence

Corrected.

Line 101: The reference cited here are for animal species. Do not describe these as “well-known histone mores of active promoters and enhancer”. Instead refer to these are histone modifications that are enriched at active promoters and enhancers in other species.

Corrected.

Line 126: I like this phrasing – simply refer to the peaks as H3K4me3 related interaction peaks. This is much more accurate than calling them promoters (or enhancers). Indeed – this section shows the flaws in calling these promoter or enhancer peaks. A large number of them are enriched at the 3’ end of genes which is not the promoter and both H3K4me3 and H3K27ac are enriched at the TTS.

For the identification of proximal PET peaks, we used PETs peaks within 2Kb of TSS of annotated gene, and not simply according to H3K4me3 signal in ChIP-seq. We also agreed to refer then as proximal PET peaks. For the identification of distal PET peaks, we also refer them as distal PET peaks in our revision.

Line 141 and figure 1d: Exactly where is the viewpoint in this figure? This needs to be indicated. The figure should also show the location of H3K27ac and H3K4me3 ChIP peaks.

Thank you for your suggestions. We have supplemented the viewpoint and the H3K27ac/H3K4me3 ChIP signal in Figure 1d in our revision.

Line 165: These lists of long range peaks (and which dataset they are found in) should be provided as a supplemental table or deposited somewhere that other researchers can access.

The lists of H3K27ac and H3K4me3 peaks and interactions had been provided in Supplemental Table 4.

Line 170-171. Do not refer to these as enhancer-like. Simply refer to these as H3K27ac peaks. This is more precise and is accurate. Unless you are going to provide data to show that these regions act as enhancers, or even that a decent number have enhancer activity it is not appropriate to refer to them as a functional unit.

We have taken you advice and defined these as “distal regulatory regions”.

Line 173: Report these full sets as a supplemental table or supplemental dataset

It had been reported in Supplementary Table 6.

Line 180: Add the details (CG and dataset) to the figure legend.

We have supplemented the details (CG and dataset) in the figure legend.

Line 205-219 are really dry. Simply summarize the terms that are enriched and if you want to refer to the genes put this in a table. I don't need a list of gene IDs in the text.

Taken your earlier suggestion, we used the term distal regulatory regions to replace enhancer-like regions. Text about "super-enhancer-like regions" is therefore not presented in our revision.

Lines 222-225: Again, I am not convinced that the authors should use the terms promoter and enhancer. I would simply recommend using proximal and distal. They can keep the current analyses but simply call them P and D as these refer to location, not putative functions. These lists (and classifications) should be reported in a supplemental table or dataset.

We have taken you advice and redefined these as "proximal (P) and distal (D) PET peaks" in our revision.

Lines 236-251 are simply summarizing numbers, many of which are better supported by the figures and I would suggest shortening this section.

We have taken you advice and supplemented figures for this part (in Fig3b, Supplemental Fig. 7d).

Lines 253-256 seem entirely redundant with lines 259. Please simplify this text.

This section has been shortened.

Lines 268-309 provide a discussion of the relationship of proximal regions to distal regions and provide some nice examples. I do not have any issues with this data but this section could be significantly shortened without losing clarity or impact.

This section has been shortened.

Line 320 This data does not show co-expression, it simply shows correlation. Co-expression is usually reserved to describe patterns across samples, not correlation of levels within a sample. On a broader note, II am not convinced that this result is worth investigating. I am much more interested in the question of whether two interacting genes are tend to exhibit co-expression throughout development. I suspect that the expression levels are not actually correlated. I suspect this result is simply driven by the fact that any two genes with PET peaks are enriched for expression (5b). This means your interactions are enriched for "on" genes with random permutations find many on-off pairs.

We had performed the co-expression analyses across large number of samples, which indicated in legend of Fig. 5.

Line 329-332 I suspect many of these genes simply do not have distal interactions, it is likely that for many maize genes the necessary regulatory elements are within several kb of the promoter.

Thank you for your suggestion. We have included this possibility in our revision.

Line 345 ‘against as expected’ is not the correct phrasing

Corrected

Line 345 What expression data is being used for this analysis? This needs to be included in the figure legends in S10 and 5.

We have supplemented the source of expression dataset in the figure legends in S10 and 5.

Line 352 In figure 5d the control of genes without interactions with distal regions should be included

We have supplemented the control of gene without interactions with distal regions in figure 5d.

Line 366 I am not convinced this is true unless this is demonstrated relative to genes without PET peaks. All the authors have demonstrated currently is that genes with intergenic interactions are more tissue-specific than genes with interactions with genic regions.

We have supplemented genes without interactions with distal regions in Figure 5d.

Line 384-387 Are these distant interactions often supported by fewer reads that closer interactions? If so, the difference among tissues might simply be due to more difficult in supporting weaker interactions.

As you suggested, we compared the number of PETs for P-D interactions with different distance. The results showed that the number of PETs for P-D interactions had an obvious decline when distance was bigger than 1Mb. But we found tissue-specific P-D had enrichment in distance 100Kb-1Mb. We have included it in Supplemental Figure 11d. Although distance may have effect in the identification of interactions, tissue-specific interactions were identified in the same distance between two tissues.

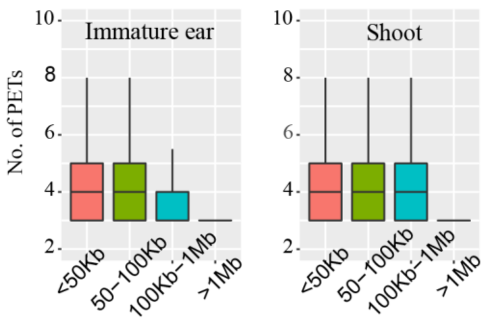

Line 407-428 many portions of this text are redundant with results already presented. This section should be substantially shortened.

This section has been shortened.

Line 431 The ChIN should be made available to other researchers in some format. This could include depositing the edges of the network or making a browser for the network.

Information about the edges of the network were shown in Supplementary Table 4 and Supplementary Table 8.

Line 430-473 I can understand the potential value of the network and the idea that genes within a set of interacting partners might have related function. However, this section is not written well and I struggled to find the specific points that authors are trying to make.

We have rewritten this section.

Line 477-478 I continue to have issues with the term enhancer. It is better to simply refer to these as distal regulatory regions. In some cases the interactions may actually be related to repression rather than activation.

We have taken you advice and revised as “distal regulatory regions (DRs)”.

Line 485: “super long distance” is probably not an appropriate term

Corrected.

Line 506-507 I would argue that your terminology is actually going to create confusion.

We have rewritten this sentence.

I think it is critical for the authors to provide a table with a full list of the coordinates of the PET peaks. These could indicate which peaks are found in both datasets and have different lists for each tissues. In addition, the full list of ChIP peaks should be reported. These files will be used by other researchers.

We had provided the full list of ChIP peaks and the coordinates of the PET peaks in Supplementary Table 4.

Reviewer #2 (Remarks to the Author):

I'm satisfied with the revised manuscript and suggest NC to publish.

Reviewer #3 (Remarks to the Author):

The revised manuscript is better. As I said last time, I think this should go to high impact journals. But it still has some errors when performing the analysis and interpreting the data. I list them below.

Line 133, In order to claim that high K4me3 or K27ac regions are enriched for long rang interaction, one needs to use the total reads (not just ChIA-PET cis-interaction PETs) in each peaks, plot them against the PET number. They can also plot the region's K4me3 or K27ac chip-seq signal against the PET number.

Here this figure used ChIA-PET both cis- and trans-interaction PETs. We compared the peak intensity from ChIP-seq between peaks with and without chromatin interactions, including both intrachr. and interchr. chromatin interactions. And we also plotted the region's K4me3 or K27ac chip-seq signal against the PET number as you suggested.

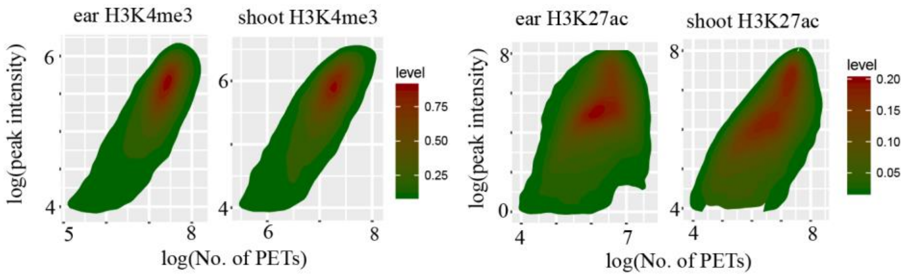

L170, I would suggest to say K27ac is a mark of active enhancer in mammalian model.

Corrected.

L200, 218-219, I still do not believe those are super enhancer or SE-like regions (simply based on the length of the K27ac peak). If they are so determined to claim this, please find those enhancers and see where they are, I bet some of them are located in poorly assembled regions, they often have higher sequencing coverage, and often called as long peak in other histone ChIP-Seq experiments unrelated to K27ac. Or maybe just look at the K4me3 data, they might have the same long K4me3 marks. Also, floral MADS genes with long first introns are known to have long K27ac peaks inside their gene. Should we call all of them SE? I hope they can do some more analysis to show the feature of those so-called SE-like regions, and maybe include them into Sup Info. If they don't want to do that, fine. Please write the discussion and result more carefully, otherwise they might regret making those strong statements in the future when new evidences have emerged showing those are not SE.

Actually, SEs identified in human and mouse were based on the signal and length of marks enriched at enhancers. Currently, many super enhancers identified by this method are not defined functionally. The SE-like regions that we identified had H3K4me3/RNA-seq signal. Since genes associated with SE were those located in the regions of SE and were highly-expressed, SEs regions are expected to overlap with highly-expressed genes.

As suggested by reviewer #1, we used the terms proximal/distal regulatory regions rather than the terms promoter/enhancer in our revision. So we have deleted the section about “SE-like regions” in our revision.

L222-224. This is a wrong strategy to annotate PET. PET 2kb away could still be a promoter to promoter interaction (e.g. an unannotated gene is there). K4me3 PET might also be an interaction between a promoter (K4me3) and a distal enhancer (region without K4me3). So they should classify them using the method in the co-summitted paper, in which promoter-promoter interaction are identified as those PET with both anchor sites associated with K4me3. The promoter to distal element PET could be defined as PET with one anchor site having K4me3 and the other anchor site having open chromatin only. Enhancer-enhancer PET will be 2 anchors associated with DHS, but no K4me3.

I am afraid that this will affect most of their following analysis, and many of them need to be re-run using the correction PET classification.

For the identification of proximal PET peaks, we used PET peaks within 2Kb of TSS of gene, consistent with genome annotation. One reason is that it was more reliable and accurate, the other reason was that there were about 16% P associating with chromatin interactions which were without H3K4me3 peaks.

For the identification of distal PET peaks, we used PET peaks 2Kb away from TSS of gene. We had considered this issue about D including those D with H3K37ac and H3K4me3 peaks. There were about 18% D with both H3K37ac and H3K4me3 peaks, 70% D only with H3K27ac peaks and remaining without both, for those D associating with P-D. Based on the analysis in our manuscript, we had found no significant differences. And we thought these regions may also be important regulatory regions associated with the regulation of gene expression. To future usage of our data, the original chromatin interaction data and ChIP-seq data as were provided in Supplemental Table 4.

For clarification, we used the terms proximal (P) and distal (D) PET peaks rather than the terms promoter-like and enhancer-like PET peaks in our revision, which are more accurate as suggested by reviewer #1.

L263-264. This is an important observation!

L 318. They now used a random control made of gene pairs of the same genome distance. But it is still missing a key component. The random gene pairs should be associated with the same K4me3 mark or K27ac.

We have answered this in next comment together.

L 321, Again, control should be genes associated with K4me3 or K27ac depends on the ChIA-PET antibody. The 2nd control will be genes associated with the same K4me3 or K27ac

ChIP-Seq signal. Because the higher the chip-seq signal, the more likely they will be identified from ChIA-PET. And we know highly expressed genes are associated with active histone marks.

Thank you for your suggestion. We have taken you advice and added the detail in Methods. We randomly shifted the loop 100 times in the same chromosome to generate lots of random pairs with the same distance. And then we filtered random pairs without H3K4me3/H3K27ac peaks or with low H3K4me3/H3K27ac ChIP-Seq signal.

L 332, What should be used as the proper control in the GO enrichment analysis? Maybe the genes most enriched with K4me3 / K27ac genes are associated with this, not the loop genes.

Thank you for your suggestion. We have taken you advice and used genes enriched with H3K4me3 / H3K27ac as control.

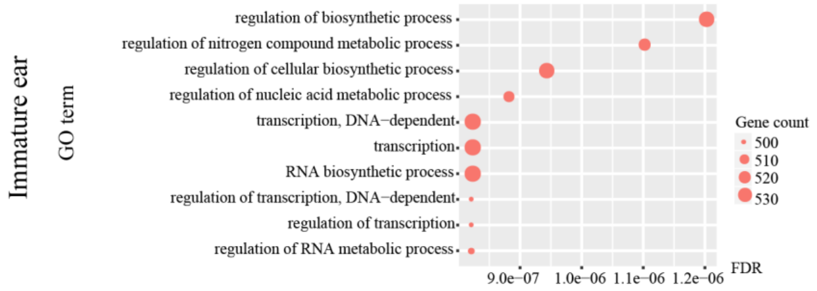

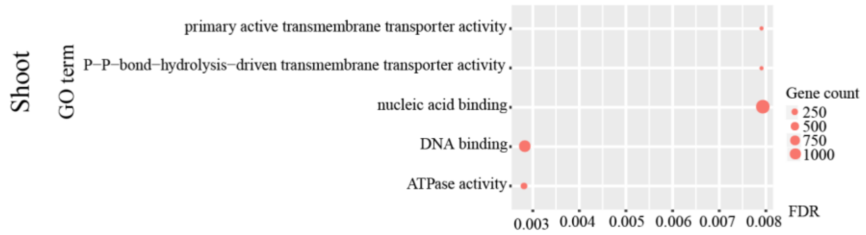

L 343. Again, they should not compare enhancer-PET genes to other non-E-PET ones. They need to find control(s). Say control group 1 could be those PET free genes with K4me3, g2 is the K27ac PET free genes. One should be very careful in making such claim…Look at the ChIP-Seq signal intensity of those genes. It might be the higher ones that got the PET, so another control will be genes associated with prompter-promoter interaction.

Thank you for your suggestion. We have taken you advice and used genes no interacting with D and enriched with H3K4me3 / H3K27ac, genes associated with prompter-promoter interactions as controls.

L348-. The English from here is hard to understand. They should be careful in defining the intergenic enhancer, and only use those without K4me3 in the “intergenic anchor region”.

As suggested by reviewer #1, we have redefined it as “intergenic D”. Here “D” refers to “distal PET peaks”.

L363, please define “genes with enhancer-like PET peaks in gene bodies”. Is it gene A is associated with PET, and the PET is from gene A to the body of gene B? If that is the case, what should be used as a control? Genome random is obviously wrong as discussed above. How about gene A associated PET with distal intergenic open chromatin? How about genes with the same K4me3 peaks but not associated with PET etc…

Here “genes with enhancer-like PET peaks in gene bodies” refers to genes have D in their gene bodies, regardless having chromatin interactions or not. We have defined it in line355-356.

We had compared the Shannon entropy of gene interacting with intergenic D, gene interacting with genic D, and gene with D in its gene body, in Fig5d. And they served as controls of each other.

L389. “Tissue-specific enhancer-like PET peaks showed enrichments in tissue-specific P-E interactions.” It doesn’t make sense. Also, please use a statistic test with proper controls for making such statement.

Thank you for your suggestion. We have taken you advice and done a statistic test with all identified chromatin interactions as control. The results showed that tissue-specific D showed enrichments in tissue-specific P-D interactions, compared with all identified chromatin interactions.

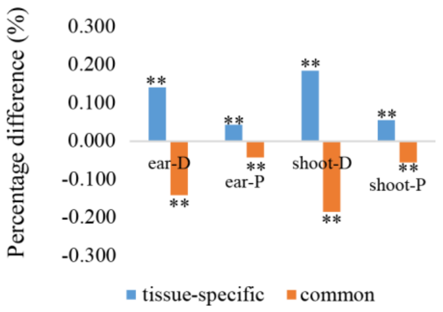

L430-439. Genes with higher degree of interaction might just be the genes with higher K4me3 or K27ac ChIP-Seq signal. So you want to claim that, please do a test with proper control.

Here we used genes with the lowest degree of interaction, which also with H3K4me3/H3K27ac signal, as control in Supplemental Figure 15d.

Reviewers' comments:

Reviewer #1 (Remarks to the Author):

The revisions by Li et al have addressed the majority of my concerns. I will just point out several remaining concerns I have in which the presented results do not fully support interpretations of the work and a couple minor issues. I remain convinced that some of the long sections with examples of specific genes or GO terms are not all that helpful but I do not think these sections are wrong and if the authors feel strongly about encouraging them that is fine.

Lines 88-93 are highly redundant (and in some cases identical) to statements in the abstract. I'd edit to make this less repetitive.

Lines 176-177. I think this math is wrong here somehow – if there are a total of 29,764 ear DRs and 25,434 of these are in both tissues and 4,957 are only in ear (these add up to 30,391). Please correct these numbers.

Lines 277-280: This forms the basis of the claim of enriched co-expression for genes with P-P interactions that is shown in the abstract. I remain a little confused about this analysis and suggest a more clear presentation. Based on the text and figure I think this is simply a comparison of two tissues which is not really co-expression but the methods seem to imply that this is based on 53 tissues. I can't really tell which it is and this should be clarified.

Further, I would encourage the authors to break this into two different questions. First, are genes with P-P interactions more likely to be expressed than other genes? Second, among expressed genes are those with P-P interactions more likely to be expressed at similar levels than other expressed genes? I am concerned that the current study is simply finding that genes with interactions are more likely to be expressed rather than finding that when genes are expressed the levels are similar. This is an interpretation that is made strongly in the abstract and I think the authors should carefully show that this is due to expression levels, not the probability of being expressed.

Reviewer #3 (Remarks to the Author):

Just a few minor things:
L281, ",and" ?
L283, what do you mean by "enriched"? Do you mean you "observed that ..."
L289, FPKM >=1 is seldom considered as "high expression". For example, ~10 FPKM equals to ~ 1 mRNA per cell.
L294, GO-term enrichment analysis
L312, I think this is impossible: gene interact with genic D in its own gene body. That means the gene will be long enough for ChIA-PET to find 2 peaks in it, and you also need sufficient PET to call these 2 peaks to be interacting with each other! Or it is self-interacting PET? Those should be discarded as noise.
L331, Why call the FPKM >=2 genes "expressed", while in the previous result section you called genes with >=1 FPKM as "highly expressed"

by the way, I found the use of acronym extremely confusing.

Reviewers' comments:

Reviewer #1 (Remarks to the Author):

The revisions by Li et al have addressed the majority of my concerns. I will just point out several remaining concerns I have in which the presented results do not fully support interpretations of the work and a couple minor issues. I remain convinced that some of the long sections with examples of specific genes or GO terms are not all that helpful but I do not think these sections are wrong and if the authors feel strongly about encouraging them that is fine.

Thank you very much for your constructive comments. Regarding the text for the examples, they may be helpful for some readers to better understand the content. We therefore prefer to keep it.

Lines 88-93 are highly redundant (and in some cases identical) to statements in the abstract. I'd edit to make this less repetitive.

We have deleted the repetition and rephrased correspondingly in our revision.

Lines 176-177. I think this math is wrong here somehow – if there are a total of 29,764 ear DRs and 25,434 of these are in both tissues and 4,957 are only in ear (these add up to 30,391). Please correct these numbers.

The distal regulatory regions (DRs) identified in immature ear and shoot are not always completely overlapped, with small portions (about 2%) of them splitting into two in the other tissue. We have clarified in our revision.

Lines 277-280: This forms the basis of the claim of enriched co-expression for genes with P-P interactions that is shown in the abstract. I remain a little confused about this analysis and suggest a more clear presentation. Based on the text and figure I think this is simply a comparison of two tissues which is not really co-expression but the methods seem to imply that this is based on 53 tissues. I can't really tell which it is and this should be clarified.

We have clarified it in our revision. It is indeed the co-expression level based on a RNA-seq dataset of 53 different seed and 25 non-seed samples.

Further, I would encourage the authors to break this into two different questions. First, are genes with P-P interactions more likely to be expressed than other genes? Second, among expressed genes are those with P-P interactions more likely to be expressed at similar levels than other expressed genes? I am concerned that the current study is simply finding that genes with interactions are more likely to be expressed rather than finding that when genes are expressed the levels are similar. This is an interpretation

that is made strongly in the abstract and I think the authors should carefully show that this is due to expression levels, not the probability of being expressed.

What we meant co-expression of the gene pairs with P-P interactions are actually “transcriptionally coordinated” that they are often transcriptionally active concurrently and with similar expression level. We have clarified this in our revision.

Reviewer #3 (Remarks to the Author):

Just a few minor things:
L281, “,and” ?

Corrected.

L283, what do you mean by “enriched”? Do you mean you "observed that ..."

Thanks for pointing this out. We have rephrased this sentence.

L289, FPKM >=1 is seldom considered as “high expression”. For example, ~10 FPKM equals to ~ 1 mRNA per cell.

We have modified it by specifically using “genes with FPKM >=1”.

L294, GO-term enrichment analysis

Corrected.

L312, I think this is impossible: gene interact with genic D in its own gene body. That means the gene will be long enough for ChIA-PET to find 2 peaks in it, and you also need sufficient PET to call these 2 peaks to be interacting with each other! Or it is self-interacting PET? Those should be discarded as noise.

We had already discarded self-interacting PETs (See Methods). So “gene interacting with genic D” are referred to gene interacting with genic D in other genes, not in its own gene body. Although it can be a bit of confusing, “gene interacting with genic D” and “gene with genic D in its own gene body” are not the same.

L331, Why call the FPKM >=2 genes “expressed”, while in the previous result section you called genes with >=1 FPKM as “highly expressed”

As suggested by reviewer#2 early, here we used a stricter standard to reduce noise for the identification of tissue-specific expressed genes between immature ear and shoot. We had

captured this in Methods.

by the way, I found the use of acronym extremely confusing.

I totally agree with you. We are not particularly satisfied with the acronym used here, but we cannot come up with much better alternative. The abbreviations (using D instead of E) were adopted as suggested by reviewer#1 earlier.

REVIEWERS' COMMENTS:

Reviewer #1 (Remarks to the Author):

The authors have done a nice job of addressing my remaining concerns. I think this version of the manuscript provides a clear description of the data and the impacts of this resource. I do not have further concerns.

Reviewer #3 (Remarks to the Author):

I have no further comments.
